# Binding affinities of the farnesoid X receptor in the D3R Grand Challenge 2 estimated by free-energy perturbation and docking

**DOI:** 10.1007/s10822-017-0056-z

**Published:** 2017-09-06

**Authors:** Martin A. Olsson, Alfonso T. García-Sosa, Ulf Ryde

**Affiliations:** 1 Department of Theoretical Chemistry, Chemical Centre, Lund University, P. O. Box 124, 221 00 Lund, Sweden; 2 Institute of Chemistry, University of Tartu, Ravila 14a, 50411 Tartu, Estonia

**Keywords:** Ligand binding, Docking, Quantum-polarised ligand docking, Free-energy perturbation, Bennett acceptance ratio, Periodic boundary conditions, Charge transformations, Drug design data resource, D3R Grand Challenge 2016

## Abstract

We have studied the binding of 102 ligands to the farnesoid X receptor within the D3R Grand Challenge 2016 blind-prediction competition. First, we employed docking with five different docking software and scoring functions. The selected docked poses gave an average root-mean-squared deviation of 4.2 Å. Consensus scoring gave decent results with a Kendall’s τ of 0.26 ± 0.06 and a Spearman’s ρ of 0.41 ± 0.08. For a subset of 33 ligands, we calculated relative binding free energies with free-energy perturbation. Five transformations between the ligands involved a change of the net charge and we implemented and benchmarked a semi-analytic correction (Rocklin et al., J Chem Phys 139:184103, 2013) for artifacts caused by the periodic boundary conditions and Ewald summation. The results gave a mean absolute deviation of 7.5 kJ/mol compared to the experimental estimates and a correlation coefficient of *R* ^2^ = 0.1. These results were among the four best in this competition out of 22 submissions. The charge corrections were significant (7–8 kJ/mol) and always improved the results. By employing 23 intermediate states in the free-energy perturbation, there was a proper overlap between all states and the precision was 0.1–0.7 kJ/mol. However, thermodynamic cycles indicate that the sampling was insufficient in some of the perturbations.

**Electronic supplementary material:**

The online version of this article (doi:10.1007/s10822-017-0056-z) contains supplementary material, which is available to authorized users.

## Introduction

The increase in computer power and advances in protein crystallography and drug discovery during the latest decades have nourished the dream that drugs one day may be developed by computational methods [1]. One of the most important properties of a drug candidate is its binding affinity to the receptor molecule and many computational approaches are available to calculate binding affinities [2]. One of the best is alchemical free-energy perturbation (FEP) [2–4], calculating the energies by exponential averaging, thermodynamic integration, Bennett acceptance ration (BAR), multi-state BAR (MBAR) or similar methods [5–8]. Being based on strict statistical-mechanics grounds, the primary limitations of FEP are the force-field employed and the sampling of the conformational space. Several recent large-scale retrospective benchmark studies have indicated that relative binding free energies of drug-like molecules to protein targets can be calculated by FEP with a mean absolute deviation (MAD) from experimental affinities of 4–6 kJ/mol [9–12]. A similar accuracy has also been reported for prospective calculations of binding affinities in host–guest systems [13, 14]. However, for protein systems, prospective predictions have typically been quite poor with MADs of 4–16 kJ/mol [15, 16], probably owing to uncertainties and variations in the binding mode.

Large-scale studies of FEP-calculated relative binding affinities have in general been restricted to charge-preserving transformations [9–16]. The reason for this is that perturbations of the net charge suffer from known artifacts in the treatment of electrostatics during molecular simulations with periodic boundary conditions and Ewald summation [17, 18], with the effect that the results depend on the size of the simulated periodic box and the software employed [19, 20]. In addition, a change in the net charge of the ligand gives rise to large and long-ranged electrostatic effects of the surrounding protein that may be hard to estimate accurately [20]. Many schemes have been suggested to correct FEP calculations for artifacts caused by the periodicity and the Ewald summation [17, 21–23]. However, they have been primarily directed towards solvation free energies of simple ions, often providing complicated and software-specific corrections. Recently, Rocklin et al. [24] and Reif and Oostenbrink [25] independently suggested general procedures to correct FEP predictions of relative binding free energies. Considering that many drug-design projects involve molecules with a varying net charge, it is important to test and calibrate methods that can handle such ligand series.

In this paper, we study the binding of 102 inhibitors to the farnesoid X receptor (FXR) [26] from the blind-prediction drug-design data resource (D3R) Grand Challenge 2016 (GC2) [27]. FXR, also known as the bile-acid receptor or nuclear receptor 1H4, has recently appeared as an interesting drug-discovery target, providing an alternative to surgical treatment of obesity [28]. The binding site of FXR is located between two flexible α-helices, such that the ligands are typically pinched between residues His-298 and Met-294 [26, 29]. This flexible binding makes FXR a challenging target for computational approaches. Moreover, the inhibitors have a varying net charge, 0 or −1. We have studied these inhibitors with two set of methods. First, we have tried to estimate the binding mode and binding affinities for all 102 ligands with five different docking and scoring methods. Second, for a subset of 33 ligands, we have tried to provide more accurate relative binding affinities by employing FEP methods. To this end, we have implemented the approach of Rocklin et al. [24] in combination with the AMBER software [30] to provide corrections for ligand transformations that involve a change in the net charge of the ligand. Thereby, we obtain a prospective benchmark test of this approach in a real drug-design problem. Furthermore, we thoroughly asses the results in terms of overlap criteria and thermodynamic cycles.

## Methods

### Protein setup

Three crystal structures were employed in our calculations. The starting structure for the docking calculations was the 3OMK structure [29], because it had the highest resolution among the available crystal structures, 1.9 Å. Moreover, it contained a benzimidazole ligand that resembled some of the challenge ligands and the binding site was large enough to accommodate all the ligands in the set. For the FEP simulations, we employed crystal structures of FXR complexed with ligands **12** and **17**, provided by the GC2 organisers in the second stage of the challenge. All structures were prepared and hydrogen atoms were added using the protein preparation wizard in the Schrödinger Maestro software [31], assuming a pH of 7.4, employed in the binding assay [27]. We also analysed possible hydrogen-bond interactions, the solvent exposure and the local surroundings of the histidine residues by local software [32] and visual inspection. Based on this analysis, we concluded that His-317, 426, 449 and 450 are protonated on the ND1 atom, whereas the other six His residues (two of which are in the ligand-binding site, His-298 and 451) are protonated on the NE2 atom. His-449 and 450 were flipped (i.e. the C and N atoms in the imidazole ring were interchanged). All water molecules were kept in the calculations.

### Docking and scoring

Before the docking, the 3OMK structure without the ligand was solvated in an octahedral box of TIP4P-Ew water molecules [33] extending at least 10 Å from the solute and was equilibrated by molecular dynamics (MD) for 10 ns. The distance between residues His-298 and Met-294 was monitored (Fig. S1) and the snapshot with the largest distance was selected for the docking (giving the most open binding site), because initial docking calculations suggested that some of the ligands were too large for the binding site.

Five docking approaches were used: Schrödinger quantum-polarised ligand docking (QPLD [34], v. 2016), Glide SP (single precision), Glide XP (extended precision) [35], AutoDock4 [36] and AutoDock Vina (Vina, version 1.12) [37], which employ different algorithms and/or scoring functions. Ligand conformational libraries were generated using LigPrep [38]. Preparation for docking with Vina was done using MGLTools [39] with ligand files from LigPrep. We employed a larger than default exhaustiveness of global search (exhaustiveness = 12, rather than 8).

For the final scoring, two different methods were used. In the first, a consensus score (CS) was employed involving the average of the five scores from QPLD, Glide SP, Glide XP, AutoDock 4 and Vina. This was done in order to hedge predictions from unreasonably high or low values. In the second, the same scoring functions were used, but the average of the ranks was used, instead of the scores (CR, consensus rank).

### Free-energy simulations

**Fig. 1:**
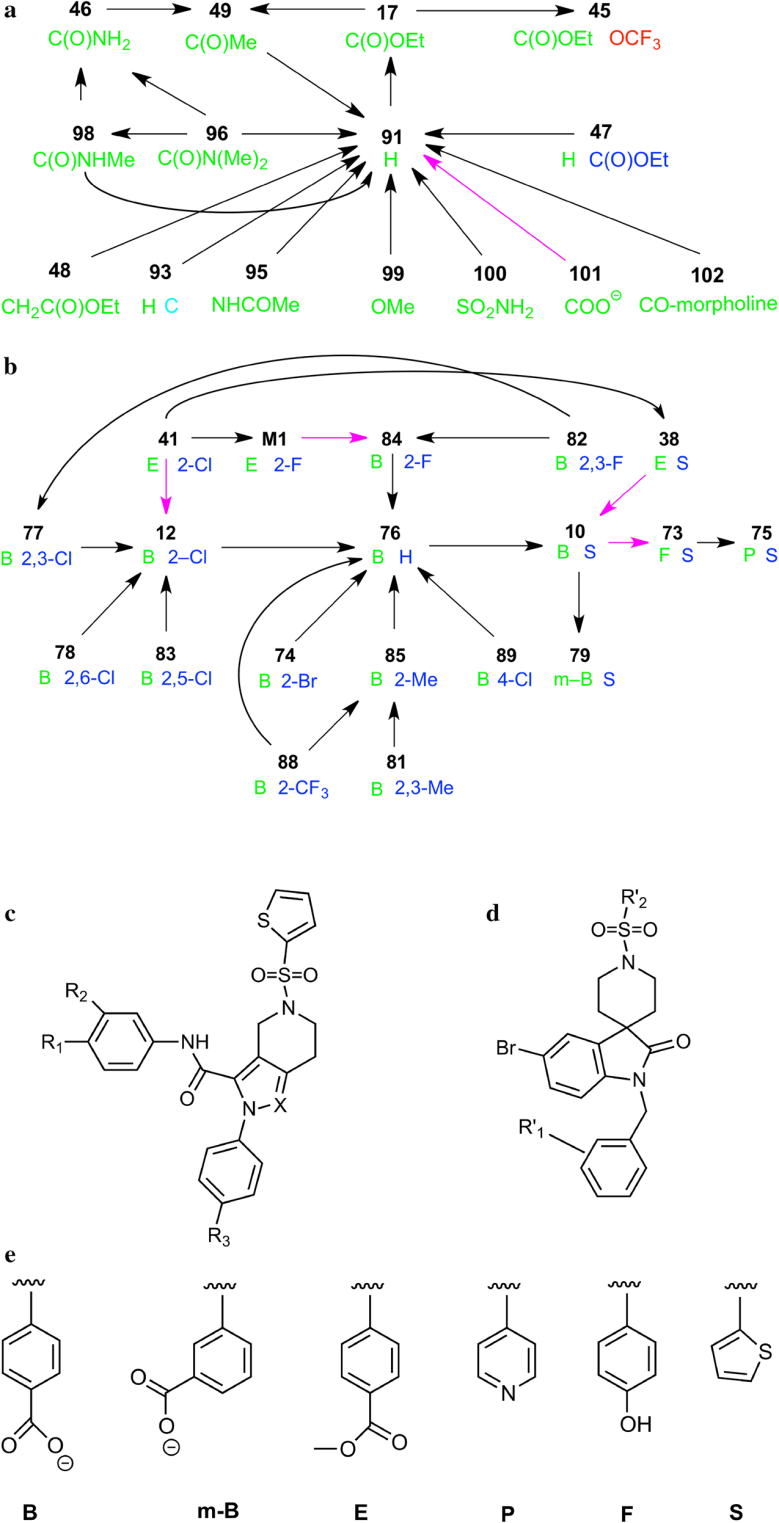
Ligands transformations studied for FEP sets 1 (**a**) and 2 (**b**) (*arrows* in* magenta* indicate perturbations modifying the net charge of the ligand). **c** and **d** show the general scaffold of the ligands in sets 1 and 2, respectively. In **a**, substituents R_1_, R_2_ and R_3_, as well as the varying atom X are shown in *green, blue, red and cyan*, respectively. Only R_1_ is shown for all ligands, whereas the other three are shown only if they differ from –H, –H and N, respectively. In **b**, the R’_1_ and R’_2_ substituents are shown in *green* (*left*) and *blue* (*right*), respectively. The former is shown in* one-letter codes*, explained in **e**, whereas the latter is shown either with S, indicating a thiophene group, also shown in **e**, or a substituted benzene ring, in which case only the substituents are shown, with the numbering starting from the position connected to the remainder of the molecule

Two sets of ligands for FEP calculations (FEP sets 1 and 2) were included in the GC2, involving 33 ligands in total. We use the numerical names of the ligands, suggested by the GC2 organisers, which are shown in Fig. 1. The FEP simulations were started from crystal structures of FXR with ligands **12** and **17** for sets 1 and 2, respectively. The other ligands were built inside the active site, based on these structures using Avogadro software [40] and the geometry was optimised with the UFF force field [41].

The ligands were manually mapped for the FEP simulations, minimising the difference between the ligands and the number of perturbations changing the net charge of the ligand. The transformations are also shown in Fig. 1. In order to assess the convergence of the binding energies, cycles were introduced when possible without introducing larger perturbations than for the other transformations (five for FEP set 1 and four for set 2). In one case, this involved the addition of an extra ligand, **M1**, also shown in Fig. 1. Five of the perturbations involved a change in the net charge of the ligand and therefore required corrections to the calculated binding free energies, viz. **101**→**91, 88**→**79, 75**→**88, 41**→**12, 88**→**73** and **M1**→**84**.

All FEP simulations were performed with the AMBER 14 and 16 software [30] with the ff14SB force-field [42] for FXR and the GAFF force field [43] for the ligands. Charges for the ligands were derived by first geometry optimising the ligands at the AM1 [44] level, followed by a calculation of the electrostatic potential at the HF/6-31G* [45] level of theory at points sampled according to the Merz–Kollman scheme [46]. These calculations were performed with the Gaussian09 [47] software. Finally, restrained electrostatic-potential charges [48] were fitted to the electrostatic potential using the antechamber program in the AMBER software [30]. The Seminario approach [49] implemented in the Hess2FF program [50] was used to obtain missing torsion parameters of the ligands, based on frequency calculations performed at the BLYP/def2-SVP level of theory [51–53]. Added parameters are listed in Table S1 in the SI.

For the FEP simulations, FXR and the ligands were solvated in a truncated octahedral box of TIP3P water molecules [54], extending at least 9 Å from the solute using the leap program in the AMBER suite, so that ~8000 water molecules were surrounding the solute (for perturbations modifying the net charge of the ligand, cubic boxes were used instead, see below). TIP3P water molecules were used for the binding affinities, because they have been shown to give the best energies [55], whereas TIP4P-Ew gave better dynamical properties [56].

The FEP simulations were run with the pmemd module of AMBER, using the dual topology scheme with both ligands in the topology file [57]. Each ligand transformation was divided into steps 25 steps, employing a linear transformation of the force-field potentials with the coupling parameter λ = 0.0, 0.025, 0.050, 0.075, 0.10, 0.15, 0.20, …, 0.80, 0.85, 0.90, 0.925, 0.95, 0.975 and 1.0. Electrostatic and van der Waals interactions were perturbed concomitantly, using soft-core potentials for both types of interactions [58, 59]. Soft-core potentials were used not only for atoms differing between the two ligands, but also for all atoms in the ligand ring systems neighbouring the perturbed group to allow for larger differences in the dynamics of the perturbed groups (atoms without soft-core potentials have identical coordinates in the perturbations).

For each λ value, 100 steps of minimisation were performed with the heavy atoms of the protein and ligand restrained towards the starting structure with a force constant of 418 kJ/mol/Å^2^. This was followed by 20 ps constant-volume equilibration with the same restraints and 2 ns constant-pressure equilibration without any restraints. Finally, a 2 ns production simulation was run for each of the 25 λ values, during which structures and energies were sampled every 2 ps.

In all the MD and FEP simulations, bonds involving hydrogen atoms were constrained with the SHAKE algorithm [60], allowing for a time-step of 2 fs. The temperature was kept constant at 300 K using Langevin dynamics [61] with a collision frequency of 2 ps^−1^, and the pressure was kept constant at 1 atm using a weak-coupling isotropic algorithm [62] with a relaxation time of 1 ps. Long-range electrostatics were handled by particle-mesh Ewald summation [63] with a fourth-order B spline interpolation and a tolerance of 10^−5^. The cut-off for Lennard-Jones interactions was set to 8 Å. No counter-ions were used in the calculations.

Relative binding free energies between two ligands, L_0_ and L_1_ (∆∆*G*_bind_), were estimated using a thermodynamic cycle that relates ∆∆*G*_bind_ to the free energy of alchemically transforming L_0_ into L_1_ when they were either bound to the protein, ∆∆*G*_bound_, or were free in solution, ∆∆*G*_free_ [64]:

1 $$\Delta \Delta {G_{{\text{bind}}}}={\text{ }}\Delta {G_{{\text{bind}}}}\left( {{{\text{L}}_{\text{1}}}} \right)-\Delta {G_{{\text{bind}}}}\left( {{{\text{L}}_0}} \right)=\Delta \Delta {G_{{\text{bound}}}}-\Delta \Delta {G_{{\text{free}}}}$$

∆∆*G*_bound_ and ∆∆*G*_free_ were estimated by the multi-state Bennett acceptance-ratio (MBAR) method [8], using the pymbar software [8], including only statistically non-correlated energies in the calculations. For comparison, BAR energies were also employed, calculated with the same software.

### Charge-transformation corrections

In this study, raw ∆∆*G*_bind_ estimates for ligand transformations that modified the net charge of the ligand were corrected for errors caused by the use of periodic boundary-conditions and Ewald summations in the FEP simulations, giving corrected binding free-energies (∆∆*G*_bind, corr_) that are independent of simulated box size. We have employed the semi-analytic correction suggested by Rocklin et al. [24]. It requires that the FEP calculations are run in a cubic periodic box with a constant volume. The free energies can then be corrected by calculating the residual integrated potential (RIP) for three non-periodic systems by numerically solving the Poisson–Boltzmann equation. All three calculations involve the protein–ligand complex with all water molecules removed. In the first calculation, the protein atoms have full charges (taken from the MM force field), whereas the ligand charges were zeroed. The other two calculations have full ligand charges but zeroed protein charges. They differ in the value of the dielectric constant of the solvent: in the first calculation (as well as in the calculation with zeroed ligand charges), the solvent dielectric constant was that of the bulk solvent (ε_s_ = 97 for TIP3P water [24, 54]). In the second calculation, the solvent dielectric constant was the same as the internal dielectric constant, which was unity in all calculations. The resulting RIPs from these three calculations will be denoted *I*_P_, *I*_L_ and *I*_L,hom_ below.

Based on these RIPs, five corrections to ∆∆*G*_bind_ were calculated, as was detailed by Rocklin et al. [24]: a correction for periodicity-induced net-charge interactions (∆*G*_NET_), a correction for periodicity-induced net-charge undersolvation (∆*G*_USV_), a correction for RIP effects (∆*G*_RIP_), an empirical correction to reproduce the exact analytical result in the special case of a single point charge at the centre of a spherical cavity (Δ*G*_EMP_) and a correction for discrete solvent effects (Δ*G*_DSC_). These five terms were calculated in the following way [24]:

2 $$\Delta {G_{{\text{NET}}}}+\Delta {G_{{\text{USV}}}}= - \frac{{{\xi _{{\text{LS}}}}}}{{8\pi { \in _0}}}\frac{{\left( {{{\left( {{Q_{\text{P}}}+{Q_{\text{L}}}} \right)}^2} - Q_{{\text{P}}}^{{\text{2}}}} \right)}}{{{ \in _s}L}}$$

3 $$\Delta {G_{{\text{RIP}}}}=\frac{{\left( {{I_P}+{I_L}} \right)\left( {{Q_P}+{Q_L}} \right) - {I_P}{Q_P}}}{{{L^3}}}$$

4 $$\Delta {G_{EMP}}= - \frac{1}{{8\pi { \in _0}}}\frac{{16{\pi ^2}}}{{45}}\left( {1 - \frac{1}{{{ \in _s}}}} \right)\left[ {{{\left( {{Q_P}+{Q_L}} \right)}^2} - Q_{P}^{2}} \right]\frac{{R_{L}^{5}}}{{{L^6}}}$$

5 $$\Delta {G_{DSC}}= - \frac{{{\gamma _s}{Q_L}{N_s}}}{{6{ \in _0}{L^3}}}$$

In these equations, *Q*_L_ and *Q*_P_ are the net charge of the ligand and the protein, respectively (−1 and −10 in the present calculations), *L* is the side length of the cubic periodic box (~7.9 nm), *N*_s_ is the number of solvent molecules in the periodic box (~14000), ε_0_ is the permittivity of vacuum, ξ_LS_ is the cubic lattice-sum (Wiegner) integration constant (–2.837), ε_s_ is the static relative dielectric permittivity of the solvent (ε_s_ = 97 for TIP3P water [24, 54]), γ_s_ is the quadrupole-moment trace of the solvent model relative to its single van der Waals interaction site, which for TIP3P is 0.00764 *e* nm^2^ (note that ref. 24 gives a 10 times too large value) and the effective solvation radius is calculated from

6 $${R_L}=\sqrt {\frac{{{I_L} - {I_{L,\hom }}}}{{\frac{1}{{8\pi { \in _0}}}\frac{{4\pi }}{3}\left( {1 - \frac{1}{{{ \in _s}}}} \right)\left| {{Q_L}} \right|}}}$$

Two sets of calculations were needed, one for the protein–ligand simulation and one for the free ligand in water solution. In the latter case, only *I*_L_ and *I*_L,hom_ can be calculated, whereas *I*_P_ = *Q*_P_ = 0 in Eqs. 2–4. Corrections are needed only for the charged ligand (the terms vanish for *Q*_L_ = 0). The final corrected binding energy was then calculated as the sum of the original binding free energy (obtained from the simulations with periodic boundary conditions and Ewald summation) and these five correction terms (taken as the difference between the corrections obtained for the protein–ligand complex and for the free ligand):

7 $$\Delta \Delta {G_{{\text{bind,corr}}}}=\Delta \Delta {G_{{\text{bind}}}}+{\text{ }}\Delta \Delta {G_{{\text{NET}}}}+{\text{ }}\Delta \Delta {G_{{\text{USV}}}}+{\text{ }}\Delta \Delta {G_{{\text{RIP}}}}+{\text{ }}\Delta \Delta {G_{{\text{EMP}}}}+{\text{ }}\Delta \Delta {G_{{\text{DSC}}~}}$$

The Poisson–Boltzmann calculations were run by the APBS software [65], using PARSE [66] radii for all atoms. A cubic grid of 257^3^ points were employed with a side length of ~80 Å for the protein–ligand complex and ~39 Å for the ligand. To ensure that the estimates are stable, the Poisson–Boltzmann calculations were performed for eight snapshots from the simulations, also allowing for an estimate of the uncertainty of the calculations. The RIPs were calculated from the APBS output by Python scripts provided by the authors of ref. [24]. We have designed a semi-automatic procedure to perform all the needed calculations, based on the AMBER FEP simulation files. The procedure and the needed files can be found in http://signe.teokem.lu.se/~ulf/Methods/ChargedFEPCorrections.html.

### Uncertainties and convergence measures

All reported uncertainties are standard errors of the mean (standard deviations divided by the square root of the number of samples). The uncertainty of the MBAR free energies calculated at each *λ* value was estimated by bootstrapping using the pymbar software [8] and the total uncertainty was obtained by error propagation (the total variance was the sum of the individual variances).

The performance of the free-energy estimates was quantified by the mean absolute deviation (MAD), the correlation coefficient (*R* ^2^), Kendall’s rank correlation coefficient (τ) and Spearman’s rank correlation coefficient (ρ) compared to the experimental data from GC2 [27]. For the FEP calculations, τ was calculated only for the transformations that were explicitly studied, not for all combinations that can be formed from these transformations (τ_r_). Moreover, it was also evaluated considering only differences (both experimental and calculated) that are statistically significant at the 95% level (τ_r,95_) [67]. It should be noted that *R* ^2^ depends on the direction of the FEP perturbation (i.e. whether **12→41** or **41→12** was considered, which is arbitrary). This was solved by considering both directions when *R* ^2^ was calculated. The standard deviation of the quality measures was obtained by a simple simulation approach [68]: for each transformation, a ∆∆*G*_bind_ result was sampled as a random number from a Gaussian distribution with the mean and standard error obtained from the MBAR calculations. The quality measures were then calculated and the procedure was repeated 1000 times. The standard error of these estimates is reported as the uncertainty. Since no uncertainty in the experimental affinities was reported [27], we assumed a typical uncertainty of 2.4 (=1.7 √2) kJ/mol [69] for these values when estimating the precision of the quality measures.

To assess the convergence of the various FEP calculations, seven overlap measures were employed [10]: the Bhattacharyya coefficient for the energy distribution overlap (Ω), the Wu & Kofke overlap measures of the energy probability distributions (*K*_AB_), as well as their bias metrics (Π), the weight of the maximum term in the exponential average (*w*_max_), the difference between the forward and backward exponential average estimate (ΔΔ*G*_EA_), the difference between the MBAR and BAR estimates (ΔΔ*G*_BAR_) and the standard deviation of the energies (σ) [10, 70–72]. Moreover, the reliability of the free-energy estimates was assessed by adding cycles among the FEP transformation, as is shown in Fig. 1. The cycle-closure hystereses give an estimate of the errors from incomplete sampling of the phase space.

## Results and discussion

As a part of the D3R Grand Challenge 2016, we have performed a prospective study of the binding of 102 inhibitors to FXR. We employed two sets of calculations: docking and scoring with five different software or scoring functions, and FEP calculations for the two FEP subsets, involving semi-analytic corrections [24] for the change in the net charge of some ligand pairs. The results of these calculations are described in separate sections.

### Docking results

102 rather diverse ligands, most of them belonging to four chemical motifs, benzimidazole, isoxazole, spiro and sulfonamides, were docked to FXR. As mentioned in the "Methods" section, we employed five different docking approaches: QPLD, Glide SP, Glide XP, AutoDock 4 and AutoDock Vina. The submitted poses were those with the lowest energy from QPLD, because we expected that this method would give the most accurate results [34, 73] (the ligand charges are polarised by the surrounding protein). In six cases, QPLD did not provide any acceptable pose (a pose that fitted into the binding site). In those cases, we used instead either the Glide XP pose if acceptable (**16**) or Vina poses (**65, 79, 80, 97** and **101**).

**Fig. 2:**
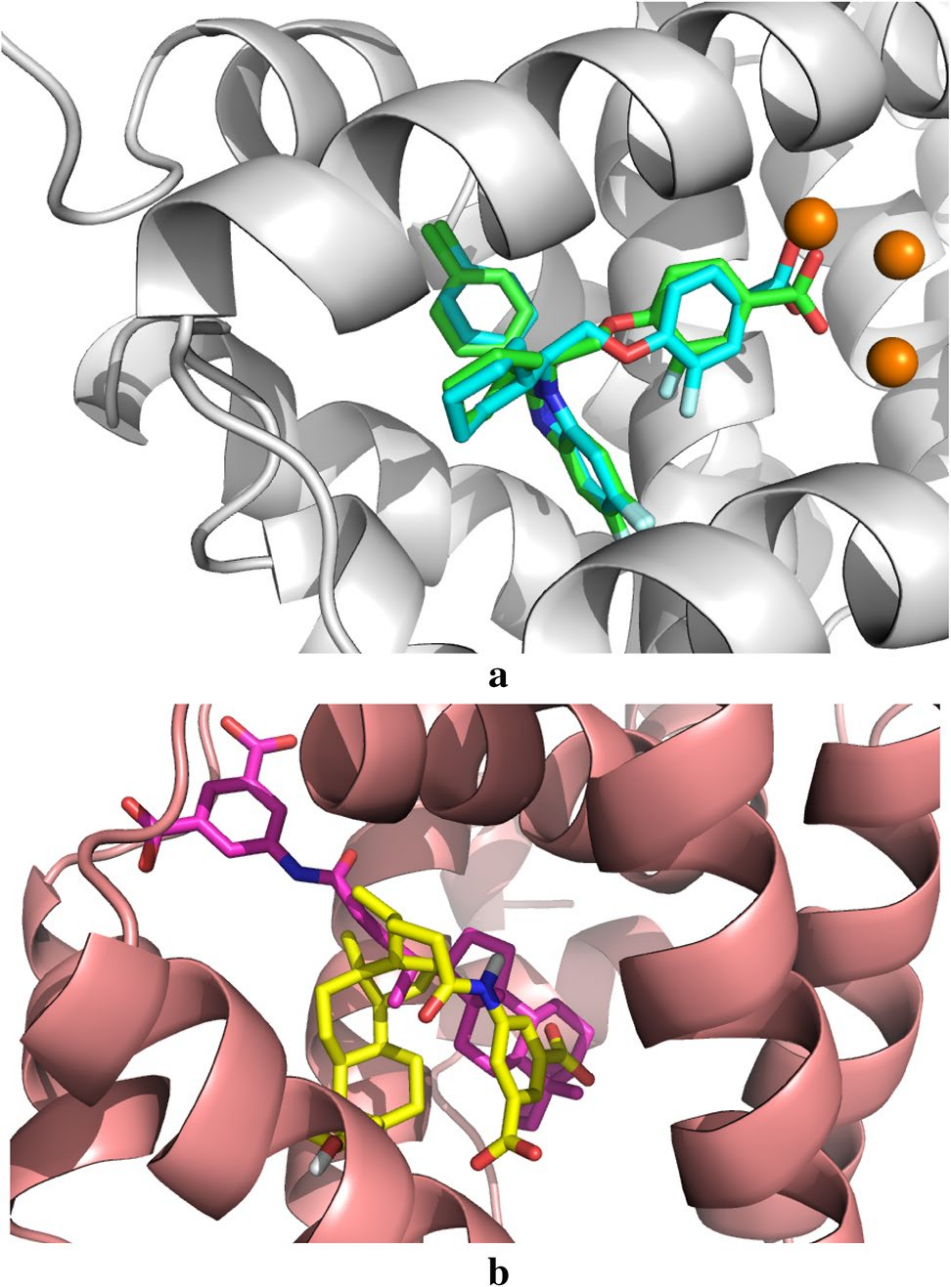
The docked poses for (**a**) compound **28** (*cyan*), which gave the lowest RMSD (1.1 Å) among our results, compared to the crystal structure (protein in *white*, ligand in *green*, water molecules in *orange*) and (**b**) for ligand **34** (*yellow*), which gave the highest RMSD (9.6 Å) among our submissions, compared to the crystal structure (protein in* salmon*, ligand in* magenta*)

After the results were submitted, crystal structures of FXR with 35 of the ligands were revealed. Our docked ligand binding poses were in line with those of the other submissions. It should be noted that we submitted only a single pose for each complex, whereas most other submissions involved more than one predicted binding pose. In several cases, reasonable poses were obtained, as can be seen in Table 1 (last column). Predictions with a root-mean-squared deviation (RMSD) from the crystal structures of 2 Å or less were obtained for 16 of the ligands (46%; **7, 13, 19, 20, 21, 22, 24, 25, 26, 27, 28, 29, 30, 31, 32** and **36**). The average RMSD for all structure predictions was 4.2 Å, which puts our results at position 22 among the 51 complete submissions for pose predictions. The best result (RMSD = 1.1 Å) was obtained for ligand **28**, which is shown in Fig. 2a. The largest RMSD was 9.6 Å for **34**, shown in Fig. 2b, for which the docking failed to reproduce the extended conformation of the ligand in the crystal structure.

**Table 1:** Results of the docking calculations with five software and scoring functions (Glide XP, Glide SP, AutoDock 4, AutoDock Vina and QPLD)

Ligand	XP	SP	AD4	Vina	QPLD	CS	CS rank	CR rank	RMSD
**1**	−35.6	−32.6	−38.2	−36.8	−38.7	−36.4	53	70	7.7
**2**	−24.0	−33.3	−36.9	−37.7	−37.0	−33.8	71	79	7.4
**3**	−35.8	−33.7	−42.6	−45.2	−41.2	−39.7	46	50	6.4
**4**	−37.1	−26.8	−45.0	−39.7	−40.9	−37.9	50	54	7.0
**5**	−37.5	−31.7	−33.6	−35.1	−38.6	−35.3	59	73	7.3
**6**	−49.2	−43.7	−48.2	−50.2	−49.0	−48.1	14	13	6.7
**7**	−55.9	−49.0	−53.5	−55.6	−58.6	−54.5	1	1	1.2
**8**	−47.5	−42.6	−42.8	−51.5	−39.0	−44.7	29	35	6.5
**9**	−36.1	−41.2	−49.1	−54.8	−40.0	−44.2	30	33	6.9
**10**	−33.8	−33.6	−25.0	−26.8	−38.6	−31.6	80	92	3.3
**11**	−26.3	−31.6	−26.0	−22.2	−37.6	−28.7	89	99	9.4
**12**	−10.0	−35.7	−26.3	−24.7	−41.0	−27.5	92	83	3.4
**13**	−41.8	−45.4	−52.5	−58.6	−41.7	−48.0	15	11	1.3
**14**	−36.9	−42.8	−47.2	−53.1	−38.8	−43.8	35	40	6.5
**15**	−43.3	−26.8	−42.3	−38.9	−40.6	−38.4	48	52	6.3
**16**	−29.9	−26.5	−32.3	−29.3	−16.7	−26.9	95	95	6.0
**17**	−39.4	−37.2	−32.4	−24.7	−41.6	−35.1	63	59	6.2
**18**	−51.0	−36.9	−39.2	−44.8	−41.5	−42.7	41	38	9.3
**19**	−51.0	−42.9	−48.1	−51.5	−54.6	−49.6	10	12	1.3
**20**	−56.1	−44.2	−49.0	−50.2	−59.1	−51.7	5	6	1.2
**21**	−54.7	−44.0	−46.9	−55.2	−56.6	−51.5	6	5	1.2
**22**	−52.2	−41.6	−45.9	−50.2	−54.2	−48.8	11	14	1.7
**23**	−46.7	−38.5	−47.1	−42.3	−41.7	−43.3	39	30	4.5
**24**	−52.8	−46.7	−46.4	−57.3	−56.6	−52.0	4	3	1.6
**25**	−56.2	−41.3	−49.9	−56.5	−59.2	−52.6	2	2	1.2
**26**	−54.1	−39.3	−50.0	−53.6	−56.3	−50.7	9	9	1.4
**27**	−54.9	−52.0	−39.7	−51.5	−40.3	−47.7	18	18	1.3
**28**	−55.9	−50.2	−42.8	−54.4	−40.3	−48.7	12	10	1.1
**29**	−55.4	−48.6	−42.0	−53.6	−40.4	−48.0	16	15	1.4
**30**	−38.7	−45.4	−34.5	−44.8	−40.0	−40.7	45	49	2.0
**31**	−51.5	−48.3	−45.7	−49.4	−41.6	−47.3	19	15	1.8
**32**	−56.7	−43.0	−38.8	−40.2	−56.1	−47.0	21	20	1.4
**33**	−35.5	−16.7	−39.4	−23.8	−40.7	−31.2	82	71	
**34**	−16.7	−17.4	−22.6	−25.9	−16.7	−19.9	101	101	9.6
**35**	−40.4	−47.1	−34.4	−43.5	−41.1	−41.3	44	43	2.5
**36**	−55.8	−53.8	−20.1	−42.7	−40.0	−42.5	42	41	1.8
**37**	−41.1	−41.0	−41.7	−43.5	−41.0	−41.7	43	42	
**38**	−37.2	−23.8	−34.7	−26.8	−41.8	−32.8	75	62	
**39**	−45.5	−43.8	−47.3	−54.0	−39.2	−46.0	24	23	
**40**	−49.0	−40.8	−43.8	−53.1	−38.1	−45.0	28	37	
**41**	−40.3	−35.4	−29.9	−23.4	−39.0	−33.6	73	77	
**42**	−47.9	−40.3	−37.6	−53.1	−41.8	−44.1	31	26	
**43**	−36.7	−23.2	−41.2	−37.2	−39.8	−35.6	58	60	
**44**	−52.1	−41.6	−42.4	−41.8	−41.0	−43.8	33	29	
**45**	−16.7	−16.7	−33.1	−15.9	−36.4	−23.8	98	100	
**46**	−38.7	−37.7	−38.7	−23.8	−41.0	−36.0	55	56	
**47**	−39.6	−36.6	−40.1	−31.4	−41.8	−37.9	51	48	
**48**	−43.8	−36.7	−32.4	−25.1	−41.0	−35.8	57	57	
**49**	−40.0	−35.8	−38.5	−21.8	−38.0	−34.8	64	72	
**50**	−52.0	−41.5	−42.0	−48.1	−51.8	−47.1	20	21	
**51**	−55.4	−39.9	−49.3	−52.3	−59.6	−51.3	7	8	
**52**	−53.3	−49.2	−48.7	−51.5	−58.3	−52.2	3	4	
**53**	−49.7	−50.3	−37.4	−52.3	−40.0	−45.9	25	27	
**54**	−53.4	−47.3	−45.4	−54.0	−53.7	−50.8	8	7	
**55**	−51.6	−43.1	−47.1	−51.0	−39.7	−46.5	22	22	
**56**	−58.5	−41.5	41.4	−32.2	−62.9	−30.7	84	39	
**57**	−47.4	−41.0	−44.6	−48.5	−16.7	−39.7	47	46	
**58**	−57.6	−51.4	−37.9	−52.7	−16.7	−43.3	37	28	
**59**	−48.2	−44.9	−29.2	−47.7	−16.7	−37.3	52	53	
**60**	−56.5	−49.5	−42.8	−52.7	−39.5	−48.2	13	17	
**61**	−43.6	−49.3	−40.9	−53.1	−38.5	−45.1	27	33	
**62**	−43.5	−49.0	−39.4	−51.0	−56.2	−47.8	17	19	
**63**	−44.1	−49.6	−33.5	−52.3	−40.8	−44.0	32	31	
**64**	−49.9	−47.2	−34.4	−45.6	−39.2	−43.3	37	44	
**65**	−9.2	−16.7	−42.2	−24.7	−16.7	−21.9	100	93	
**66**	−50.7	−43.3	−37.9	−44.8	−40.7	−43.5	36	36	
**67**	−42.2	−43.0	−11.8	−35.1	−41.0	−34.6	65	55	
**68**	−49.9	−47.2	−33.2	−45.6	−39.2	−43.0	40	45	
**69**	−40.9	−51.6	−15.8	−40.6	−41.3	−38.0	49	47	
**70**	−52.0	−39.0	−49.5	−52.3	−39.7	−46.5	23	23	
**71**	−43.9	−44.2	−39.2	−49.8	−41.8	−43.8	34	25	
**72**	−55.4	−50.0	−36.9	−48.1	−39.2	−45.9	26	32	
**73**	−34.0	−36.6	−36.3	−28.0	−40.5	−35.1	62	65	
**74**	−38.1	−39.2	−26.7	−21.8	−39.0	−33.0	74	75	
**75**	−24.8	−33.3	−35.8	−29.3	−37.8	−32.2	79	87	
**76**	−39.2	−35.9	−28.4	−27.6	−41.2	−34.4	67	60	
**77**	−32.5	−16.7	−24.5	−25.9	−38.9	−27.7	91	97	
**78**	−34.9	−37.1	−20.3	−14.2	−36.4	−28.6	90	96	
**79**	−21.7	−37.2	−29.5	−28.5	−16.7	−26.7	96	90	
**80**	−41.4	−33.6	−31.1	−26.4	−16.7	−29.8	86	79	
**81**	−37.0	−24.8	−26.0	−24.3	−39.7	−30.4	85	89	
**82**	−40.9	−38.5	−23.0	−26.4	−41.8	−34.1	68	57	
**83**	−27.3	−39.1	−27.2	−20.5	−39.7	−30.8	83	86	
**84**	−36.2	−38.1	−25.6	−28.0	−40.9	−33.8	72	66	
**85**	−14.6	−36.1	−29.9	−27.6	−40.4	−29.7	88	76	
**86**	−16.7	−16.7	−13.2	−16.7	−33.6	−19.4	102	102	
**87**	−34.6	−27.4	−29.7	−30.1	−39.4	−32.2	77	83	
**88**	−14.5	−35.5	−24.8	−18.4	−38.5	−26.4	97	98	
**89**	−40.0	−36.9	−23.6	−20.9	−41.6	−32.6	76	68	
**90**	−38.7	−22.0	−42.8	−36.4	−41.8	−36.3	54	51	
**91**	−35.8	−35.1	−39.0	−30.1	−39.7	−35.9	56	63	
**92**	−33.7	−32.3	−35.8	−33.5	−37.6	−34.6	66	82	
**93**	−33.3	−34.5	−36.9	−29.7	−34.9	−33.8	70	79	
**94**	−32.7	−36.3	−39.2	−29.7	−38.4	−35.3	61	69	
**95**	−33.4	−34.0	−19.5	−20.5	−41.7	−29.8	87	85	
**96**	−42.0	−37.0	−20.5	−15.5	−41.8	−31.3	81	66	
**97**	−32.5	−28.0	−31.1	−28.9	−16.7	−27.5	93	94	
**98**	−40.3	−35.2	−22.3	−23.0	−40.2	−32.2	78	77	
**99**	−36.0	−30.8	−37.0	−26.4	−39.4	−33.9	69	74	
**100**	−38.7	−37.5	−38.0	−22.6	−39.6	−35.3	60	64	
**101**	−22.1	−35.5	−36.3	−25.1	−16.7	−27.1	94	91	
**102**	−45.0	−16.7	4.0	−11.7	−40.8	−22.0	99	87	

The docking scores are in kJ/mol

*CS* consensus score, *CR* consensus rank, *RMSD* root-mean-quared deviation from the crystal structures in Å [27]

Our two scoring functions, CS and CR, (submitted before the crystal structures were revealed) gave nearly identical results compared to the experimental affinities [27]: Kendall’s τ was 0.26 ± 0.06 for both, whereas the Spearman’s ρ was 0.40 ± 0.09 and 0.41 ± 0.08, respectively, as calculated by the GC2 organisers. These results were in the middle among the submissions, at positions 34 and 35, respectively, out of 59 submissions.

For simplicity, we submitted only one docking pose, the one with the lowest score. Different methods can be devised to use more than one pose, e.g. by combining the scores from several poses or by providing several poses with varying scores. Given that our procedure also included several docking programs and their different algorithms and scoring functions, we decided to use the consensus ranks and scores. Other protein crystal structures may have been used, but we found the structure chosen suitable for the task. The flexibility and dynamics of the binding site and ligands may also have been explored, but the given time was not enough for a deeper study.

### FEP results

We have estimated the relative binding affinities of 33 ligands of FXR by FEP calculations with the AMBER software. The ligands were divided by the organiser into two sets: FEP set 1, involving 18 sulfonamide ligands, and FEP set 2, with 15 spiro ligands. We set up two networks involving 19 and 20 transformations for the two sets, respectively, to obtain relative affinities of all the ligands and also to check the convergence with some thermodynamic cycles, as is shown in Fig. 1. The transformations were selected to minimise the difference between the ligands and they involve changes ranging from single-atom transformations (e.g. H→F/Cl/Br) to the introduction of a –CO–morpholine group. Five of the transformations involved a change in the net charge of the ligand and therefore required correction terms when simulated under periodic boundary-conditions with Ewald summation. We have therefore implemented the procedure suggested by Rocklin et al. [24] in connection with FEP free energies calculated with the AMBER software.

**Fig. 3:**
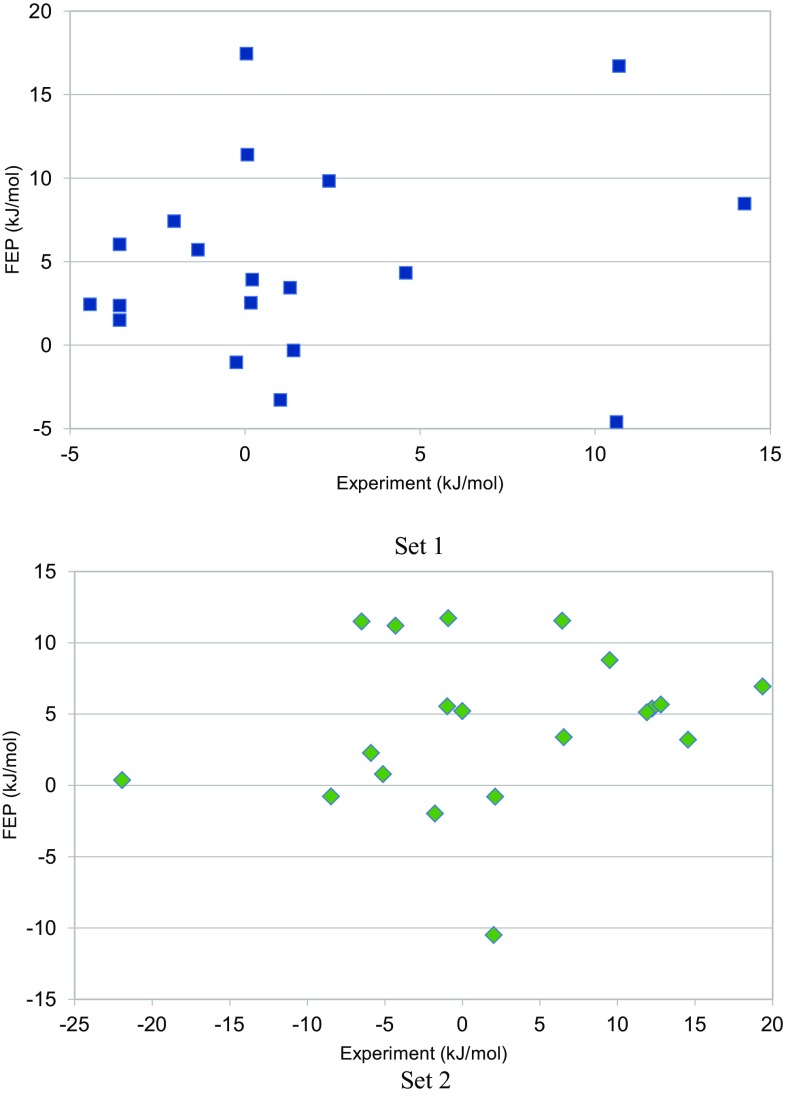
Comparison between the experimental [27] and calculated binding free energies for the two FEP sets

The results of the various FEP calculations are presented in Table 2. Compared to the experimental results [27], we obtained mean absolute deviation (MAD) of 7.5 ± 0.4 kJ/mol. This is slightly worse than in previous retrospective studies (4–6 kJ/mol) [9–12], but better than in the previous D3R Grand Challenge 2015 (4–16 kJ/mol) [15, 16]. The MAD was somewhat lower for set 1 (6.4 ± 0.5 kJ/mol) than for set 2 (8.6 ± 0.5 kJ/mol). The correlation between the calculated and experimental results was low, *R* ^2^ = 0.08 ± 0.02. It was similar for the two sets, as can also be seen in Fig. 3. The τ_r_ was also poor, 0.05 ± 0.11, but it improved if relative affinities (both computed and experimental) were considered only if they were significantly different from zero at the 95% level (τ_95_ = 0.29 ± 0.04) [67]. This reflects that there are many experimental relative affinities with a small magnitude and therefore an uncertain sign (cf. Table 2). It seems more reasonable to exclude these in the calculations of τ.

**Table 2:** Calculated (with and without charge correction) and experimental [27] relative binding free energies (kJ/mol) for the two FEP sets

Perturbation	∆Δ*G*_bind_		∆Δ*G*_CC_		∆Δ*G*_bind,corr_		∆Δ*G*_exp_
FEP Set 1							
**17→45**	−4.6	±0.2			−4.6	±0.2	10.6
**17→49**	8.5	±0.2			8.5	±0.2	14.3
**17→91**	16.7	±0.3			16.7	±0.3	10.7
**45→91**	11.4	±0.4			11.4	±0.4	0.1
**46→49**	−0.3	±0.1			−0.3	±0.1	1.4
**47→91**	−3.3	±0.3			−3.3	±0.3	1.0
**48→91**	6.0	±0.4			6.0	±0.4	−3.6
**49→91**	1.5	±0.3			1.5	±0.3	−3.6
**93→91**	5.7	±0.1			5.7	±0.1	−1.3
**95→91**	−1.0	±0.3			−1.0	±0.3	−0.2
**96→46**	2.5	±0.3			2.5	±0.3	0.2
**96→91**	7.4	±0.3			7.4	±0.3	−2.0
**96→98**	2.5	±0.2			2.5	±0.2	−4.4
**98→46**	4.3	±0.2			4.3	±0.2	4.6
**98→91**	9.8	±0.3			9.8	±0.3	2.4
**99→91**	2.4	±0.2			2.4	±0.2	−3.6
**100→91**	3.4	±0.4			3.4	±0.4	1.3
**101→91**	−4.5	±0.6	8.4	±0.3	3.9	±0.7	0.2
**102→91**	17.4	±0.4			17.4	±0.4	0.0
FEP Set 2							
**10→73**	−17.4	±0.5	6.9	±0.2	−10.5	±0.6	2.0
**10→79**	11.7	±0.5			11.7	±0.5	−0.9
**12→76**	6.9	±0.2			6.9	±0.2	19.4
**38→10**	7.1	±0.4	−7.9	±0.3	−0.8	±0.5	−8.5
**41→12**	7.2	±0.4	−6.9	±0.2	0.4	±0.5	−21.9
**41→38**	5.2	±0.2			5.2	±0.2	0.0
**41→M1**	2.1	±0.2			2.1	±0.2	
**73→75**	11.5	±0.2			11.5	±0.2	6.4
**74→76**	5.4	±0.2			5.4	±0.2	12.2
**76→10**	2.3	±0.2			2.3	±0.2	−5.9
**77→12**	11.2	±0.1			11.2	±0.1	−4.3
**77→82**	5.5	±0.2			5.5	±0.2	−1.0
**78→12**	−0.8	±0.2			−0.8	±0.2	2.1
**81→85**	11.5	±0.3			11.5	±0.3	−6.5
**82→84**	8.8	±0.2			8.8	±0.2	9.5
**83→12**	0.8	±0.2			0.8	±0.2	−5.1
**84→76**	3.4	±0.1			3.4	±0.1	6.5
**85→76**	3.2	±0.1			3.2	±0.1	14.6
**88→76**	5.7	±0.2			5.7	±0.2	12.8
**88→85**	−2.0	±0.3			−2.0	±0.3	−1.8
**89→76**	5.1	±0.2			5.1	±0.2	11.9
**M1→84**	9.7	±0.4	−6.8	±0.2	2.9	±0.5	

As mentioned above, five of the studied transformations involved a change in the net charge of the ligand and for these we employed the semi-analytic correction suggested by Rocklin et al. [24]. As can be seen from Table 2, this correction amounted to 7–8 kJ/mol in all cases, with a positive sign if the starting ligand was charged and a negative sign if the final ligand was charged (the net charge of the ligands was either 0 or −1). The individual terms are shown in Table S3. The charge-correction calculations took only ~5 min/snapshot and can easily be automatised. For the four transformations with experimental data available, the charge correction always led to a reduced error and in two of the cases, it also corrected the sign of the result. Thus, it improved all quality measures (without the correction MAD = 8.1 ± 0.4 kJ/mol, *R* ^2^ = 0.03 ± 0.01 and τ_r_ = −0.05 ± 0.10). Thus, the charge correction seems to be reliable and significantly improves the results. Excluding the four charge perturbations from the evaluation gave slightly better quality measures (MAD = 7.1 ± 0.4 kJ/mol, *R* ^2^ = 0.11 ± 0.02 and τ_r_ = 0.06 ± 0.10) than if they were included, but the improvements are small and none of them is statistically significant.

Still, the largest deviation was observed for the **41→12** transformation in set 2 (22 kJ/mol), which involves the transformation of a benzoate group to the corresponding methyl ester, i.e., a charge perturbation. On the other hand, the other three charge transformations had smaller errors, 4–13 kJ/mol, and there was no correlation between the sign of the charge correction and the error. The other four transformations with an error larger than 15 kJ/mol involved the largest perturbation (**102→91**, i.e. –CO-morpholine→H), the introduction of a –OCF_3_ group (**17→45**) and two simple H→Cl transformations (**77→12** and **81→85**). From this, it is hard to suggest a general explanation of the poor results for many of the transformations.

The precision of the calculated affinities is also given in Table 2. It can be seen that it was small for all transformations, 0.1–0.7 kJ/mol. The charge correction added an extra term with an uncertainty of 0.2–0.3 kJ/mol, so these transformations always gave the higher uncertainties (0.5–0.7 kJ/mol, compared to 0.1–0.5 kJ/mol for the other transformations). However, the charge perturbations gave a high uncertainty already without the charge corrections (0.4–0.4 kJ/mol), reflecting that a change of the net charge of the ligand gives rise to large fluctuations of the electrostatic interaction with the surrounding protein. Still, it is clear that the rather poor results (e.g. MAD = 7.5 kJ/mol) are not caused by a too low precision (0.1–0.7 kJ/mol).

Likewise, there is no indication of any poor overlap in any of the studied transformations. On the contrary, the seven overlap measures listed in Table S2 all indicate proper overlap throughout the transformations. In fact, we first run some of the transformations with only 13 λ values, but the overlap measures sometimes indicated poor overlap. Therefore, we decided to use 25 λ values for all transformations.

On the other hand, the thermodynamic cycles indicate an appreciably poorer convergence of the results, as can be seen in Table 3. Of the nine studied cycles, only two gave a vanishing result, within the statistical precision, both in FEP set 2, one of which involves two charge-perturbation steps and the extra **M1** ligand (**76→12→41→M1→84→76**, 1.2 ± 0.7 kJ/mol; also **76→12→77→82→84→76**, −0.4 ± 0.4 kJ/mol). The other six cycles gave larger hystereses, 4–10 kJ/mol. The one with the largest hysteresis (**91→17→45→91**) involves the two perturbations (**45→91** and **17→45**) for which BAR and MBAR gave results that differ significantly (by 1.5 and 4.6 kJ/mol; cf. ∆∆*G*_BAR_ in Table S2), whereas for all the other transformations, the difference was less than 1.2 kJ/mol (0.5 kJ/mol on average). They involve the introduction of –OCF_3_ and –COOEt groups. Large cycle-closure errors indicate that sampling of the phase space has been incomplete. This may be caused by a change of the binding mode of the ligands. However, we have not been able to identify such problems by overlaying the structures. The problems could perhaps have been solved by longer simulations or enhanced-sampling techniques. Alternatively, several independent perturbations could have been run, which often give a better estimate of the true uncertainty and a more effective sampling of the phase space [13, 74, 75]. In fact, test calculations indicated that ∆∆*G*_bind_ from independent repeats varies by ~2 kJ/mol.

**Table 3:** Thermodynamic cycles and the cycle hysteresis (kJ/mol)

Cycle	Hysteresis
**91→49→17→91**	6.7 ± 0.4
**91→17→45→91**	−9.9 ± 0.6
**91→96→46→49→91**	−3.7 ± 0.5
**96→98→46→96**	4.2 ± 0.4
**91→96→98→91**	4.9 ± 0.5
**76→12→41→M1→84→76**	1.2 ± 0.7
**76→12→77→82→84→76**	−0.4 ± 0.4
**76→12→41→38→10→76**	−5.1 ± 0.8
**76→85→88→76**	4.5 ± 0.4

In the GC2 evaluation, the relative binding affinities were recalculated to absolute affinities, by employing **10** and **17** as reference ligands for sets 1 and 2, respectively. This makes the results dependent on the selected reference ligand (ligands **76** and **91** would have been more natural, based on our perturbation networks, shown in Fig. 1, whereas ligand **10** is very peripheral) and make the uncertainties more varying, as they depend on the number of perturbations needed to reach the various ligands from the selected reference. Still, this is necessary to enable a comparison between the various methods.

In the evaluation of the various submissions (22 for both FEP sets, although only 18 and 19 involved all ligands for set 1 and 2, respectively), our results gave τ = 0.02 ± 0.22, ρ = 0.12 ± 0.27, *R* = 0.34 ± 0.27 and RMSD = 6.3 ± 1.3 kJ/mol for set 1 and τ = 0.48 ± 0.14, ρ = 0.66 ± 0.14, *R* = 0.58 ± 0.13 and RMSD = 6.3 ± 0.8 kJ/mol for set 2. *R* for set 1 was the second best among all submissions, whereas most of the other entries ranked number five, except τ and ρ for set 2 (12–16). However, our method gave relatively accurate results for both sets and also comparable results for all measures, whereas most other methods gave more varying results. Therefore, our method was among the four submissions that gave the best results for both FEP sets. Two of the other top submissions also employed FEP, using the Schrödinger software and the OPLS3 force field (submissions pyxiv and x2j7p by Cournia group and submissions ck8kc and 81n55 by an anonymous group). Both gave the same average RMSD as our submission, 6 ± 1 kJ/mol. The third submission (3idpo and rvm67 by Camacho group), used instead the “quasi-exact” scoring approach, which actually gave the lowest RMSD for set 1, 4.9 kJ/mol, but worse average τ, ρ and *R* results than our submission. None of the four quality estimates showed any statistically significant differences for any of the two FEP sets between our results and those of the other three top submissions. FEP calculations by the Michel group also gave low RMSD, but they had problems with the charge perturbations and the best results were obtained when those perturbations were excluded. The FEP calculations with the Schrödinger software employed only neutralised ligands. Other approaches, including MM/GBSA, MM/PBSA, multi-site lambda dynamics and also one set of FEP calculations gave clearly worse results.

Our FEP results can also be compared to those obtained with the consensus score (CS) from our docking calculations. To this end, we took the difference of the CS results for the two ligands involved in the same perturbations studied by FEP (Table 2). Interestingly, CS gave results of nearly the same quality as FEP: the MAD was slightly lower for FEP set 1 (5.5 ± 0.8 kJ/mol compared to 6.4 ± 0.5 kJ/mol), but slightly higher for set 2 (10.0 ± 0.9 kJ/mol, compared to 8.6 ± 0.5 kJ/mol; standard errors for CS were estimated from the standard deviation over the ∆∆*G*_bind_ results for each of the five scoring methods and it was much higher than for FEP, 1–9 kJ/mol). On the other hand, the correlation was worse for both sets, *R* ^2^ = 0.04 ± 0.04 and −0.46 ± 0.10 (i.e. an anticorrelation), compared to 0.09 ± 0.02 and 0.08 ± 0.02. τ_r_ was slightly better for set 1, but appreciably worse for set 2, 0.16 ± 0.21 and − 0.47 ± 0.19, compared to 0.05 ± 0.11 and 0.05 ± 0.12. The poor τ results, compared to those calculated for all 102 ligands (0.26 ± 0.06), indicates that the binding affinities in the FEP sets were harder to estimate than the those of the other ligands.

## Conclusions

In this investigation, we have studied the binding of 102 ligands to FXR from the blind-prediction D3R Grand Challenge 2016 with five different docking and scoring methods. Considering that we only provided a single pose for each ligand, the results were decent, in the middle among the GC2 submissions, and comparable to some FEP results. The scoring gave fairly good results with a τ of 0.26 ± 0.06 and a ρ of 0.41 ± 0.08, especially considering that only one protein structure was used for all ligands. Better results may perhaps have been obtained with more relevant crystal structures or considering more flexibility of the binding site, fixing parts of the ligand, demanding certain protein–ligand interactions to be fulfilled for the docking programs, using more than one binding pose for scoring or using even higher exhaustiveness settings.

Moreover, we have employed a FEP protocol to calculate relative binding free energies for the 33 ligands in the FEP set. In particular, we have implemented and benchmarked the approach of Rocklin et al. [24] to correct for artefact caused by the periodic simulations with Ewald summation for transformations that changed the net charge of the ligand. The accuracy is slightly worse than in retrospective large-scale tests of FEP methods [9–12] (MAD = 7.5 kJ/mol, *R* ^2^ = 0.1 and τ_r,95_ = 0.3), but better than in the D3R Grand Challenge 2015 [15, 16]. The charge corrections are significant (7–8 kJ/mol) and always improve the results. The precision of the estimated binding affinities is good (0.1–0.7 kJ/mol) and our measures indicate that the overlap throughout the transformations is excellent, owing to the use of 25 λ values. However, the thermodynamic cycles indicate that the sampling in several cases has been unsatisfactory. This could have been resolved by more simulations (although the time was limited). Moreover, it is possible that we have employed incorrect structures or that the binding mode changes for the various ligands (only three crystal structures are available for the studied ligands), which may explain the rather poor results.

Interestingly, FEP calculations with the Schrödinger FEP software and the latest force field OPLS3 [12] did not give any significantly better results, although they involved longer simulations (5 ns), enhanced-sampling methods and automatic mapping of the ligands. The reason for this may be that they did not employ any charge corrections, but instead supposed that all ligands were neutral when binding. The prime conclusion of this prospective study is that the charge corrections are large (7–8 kJ/mol) and significantly improve the results. The correction employed in this investigation [24] is easy to implement and does not increase the computational load significantly.

## Electronic supplementary material

Below is the link to the electronic supplementary material.

Supplementary material 1 (PDF 253 KB)
