# Supplementary material for: Binding affinities of the farnesoid X receptor in the D3R Grand Challenge 2 estimated by free-energy perturbation and docking
Source: J Comput Aided Mol Des. 2017 Sep 6;32(1):211–24. doi: 10.1007/s10822-017-0056-z (PMC5767205; doi:10.1007/s10822-017-0056-z)
Supplement: Supplementary file 1 — Supplementary material 1 (PDF 253 KB) [file 10822_2017_56_MOESM1_ESM.pdf]

Supporting information

Relative binding affinities of the farnesoid X receptor in the D3R Grand Challenge 2 estimated by free-energy perturbation

Martin A. Olsson[1], Alfonso T. García-Sosa[2], Ulf Ryde [1,*]

[1] Department of Theoretical Chemistry, Lund University, Chemical Centre, P. O. Box 124, SE-221 00 Lund, Sweden

[2] Institute of Chemistry, University of Tartu, Ravila 14a, Tartu 50411, Estonia

Correspondence to Ulf Ryde, E-mail: Ulf.Ryde@teokem.lu.se,
Tel: +46 – 46 2224502, Fax: +46 – 46 2228648

2017-06-01

Figure S1. Distance between residues His-298 and Met-332 in a MD simulation of ligand-free FXA. These two residues are known to interact with and pinch the FXR ligand, located in a pocket formed by two flexible α-helices. We selected the structure with the largest distance (15.4 Å at 0.75 ns).

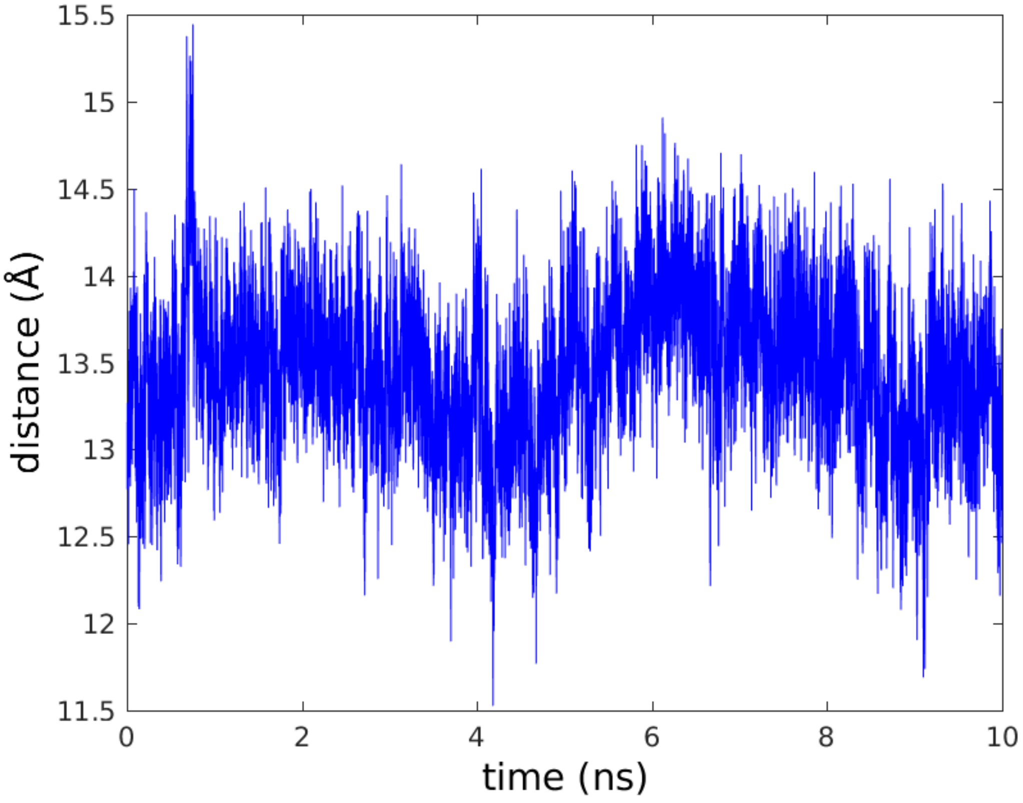

Table S1. Added angle and dihedral parameters for the ligands. The first column give the involved GAFF atom types. k_a and k_d are the force constants (in kcal/mol), α_0 is the equilibrium angle (in degrees), n is the periodicity and φ is the phase (in degrees).

Angle		k_a	α_0
na-cc-c		178.174	128.924
Dihedral	n	k_d	φ
cd-cc-ss-cc	2	19.011	180
c-ss-cc-h4	2	9.391	180
o-sy-cc-cd	6	0.717	0
o-sy-cc-ss	6	0.526	0
sy-cc-ss-cc	2	21.128	180
n3-sy-cc-cd	6	0.809	0
n3-sy-cc-ss	6	0.603	0
cd-cc-ca-ca	2	10.395	0
n-cc-ca-ca	2	9.303	0

Table S2. Convergence metrics for all free-energy perturbations of FE set 1 & 2.
The overlap measures are described in the main text. They are the maximum or minimum value over the 25 λ values and simulations of both the protein–complex and the ligand free in solution. None of the values violate any of the suggested overlap criteria ($\Omega > 0.85$, $|K_{AB} - 1| < 0.25$, $\Pi > 0.5$, $w_{max} < 0.5$ and $\Delta\Delta G_{EA} < 2$ kJ/mol [M. A. Olsson, U. Ryde (2017) J. Chem. Theory Comput., 13, 2245-2253]).

Perturbation	Ω	K_{AB}	Π	w_{max}	$\Delta\Delta G_{EA}$	$\Delta\Delta G_{BAR}$	σ
FEP Set 1							
**17→45**	0.98	0.91	1.6	0.03	1.6	-1.5	1.0
**17→49**	0.97	0.91	1.7	0.01	1.1	0.1	1.0
**17→91**	0.97	0.92	1.3	0.02	2.0	-0.4	1.1
**45→91**	0.98	0.91	1.4	0.15	3.7	4.6	2.8
**46→49**	0.97	0.89	1.9	0.01	0.7	-0.7	0.9
**47→91**	0.98	0.94	1.7	0.04	2.2	-0.2	1.6
**48→91**	0.98	0.92	1.7	0.05	1.3	0.7	1.5
**49→91**	0.98	0.95	1.7	0.02	1.3	0.8	1.0
**93→91**	0.98	0.90	1.3	0.01	0.7	0.3	0.5
**95→91**	0.98	0.91	1.4	0.03	2.3	0.7	1.5
**96→46**	0.97	0.87	1.6	0.02	1.5	0.0	1.3
**96→91**	0.98	0.91	1.6	0.05	1.5	-0.5	1.5
**96→98**	0.98	0.95	1.6	0.02	1.4	-0.6	1.3
**98→46**	0.98	0.97	1.2	0.03	1.5	-0.5	1.4
**98→91**	0.98	0.93	2.0	0.03	1.8	0.0	1.4
**99→91**	0.98	0.94	1.7	0.02	2.1	0.4	1.2
**100→91**	0.98	0.90	1.9	0.03	2.4	0.8	1.4
**101→91**	0.97	0.96	1.5	0.02	2.0	0.9	1.9
**102→91**	0.98	0.96	1.6	0.05	2.0	-0.4	1.7
FEP Set 2							
**10→73**	0.98	0.97	1.5	0.04	2.9	0.4	2.0
**10→79**	0.98	0.92	1.8	0.06	3.2	-0.3	2.4
**12→76**	0.98	0.95	2.0	0.01	0.8	0.4	0.7
**38→10**	0.98	0.95	1.6	0.03	2.3	-0.4	2.1
**41→12**	0.98	0.96	1.8	0.08	1.2	0.1	1.9
**41→38**	0.98	0.92	2.0	0.01	0.9	0.1	1.0
**41→L1**	0.97	0.94	2.1	0.01	0.7	0.1	1.1
**73→75**	0.98	0.96	1.9	0.02	1.4	0.3	1.2
**74→76**	0.97	0.93	2.2	0.03	0.6	0.1	1.0
**76→10**	0.98	0.86	1.7	0.01	0.7	1.2	0.8
**77→12**	0.98	0.96	1.5	0.01	0.7	0.6	0.6
**77→82**	0.97	0.93	1.9	0.03	1.5	-0.5	1.2
**78→12**	0.98	0.92	2.1	0.02	0.6	0.0	1.2
**81→85**	0.98	0.91	1.9	0.03	1.0	0.0	1.4
**82→84**	0.98	0.92	1.8	0.01	0.7	0.2	0.6
**83→12**	0.98	0.94	1.8	0.01	0.7	0.7	1.2
**84→76**	0.97	0.97	1.8	0.01	0.4	-0.1	0.5
**85→76**	0.97	0.87	2.2	0.01	0.7	-0.2	0.7
**88→76**	0.97	0.98	1.9	0.03	1.0	0.4	1.0
**88→85**	0.98	0.95	1.9	0.02	0.8	-0.2	1.0
**89→76**	0.98	0.93	1.8	0.01	1.5	-0.5	0.7
**L1→84**	0.97	0.92	1.5	0.02	1.2	0.5	1.9

Table S3. Terms in the charge corrections for the various charge perturbations. The term are explained in the Methods section. Lengths are in nm, RIPS in kJ nm^3/mol/*e* and energies in kJ/mol. Uncertainties are standard errors over eight snapshots.

	**10→73**	**38→10**	**41→12**	**M1→84**	**101→91**
Q_P	-10	-10	-10	-10	-10
Q_L	-1	-1	-1	-1	-1
Protein–ligand simulation					
N_s	14458	14424	14458	14458	13685
L	7.9 ±0.0	7.9 ±0.0	7.9 ±0.0	7.9 ±0.0	7.7 ±0.0
I_L	-169.6 ±6.0	-173.7 ±8.6	-180.4 ±7.0	-182.3 ±6.9	-232.3 ±6.9
$I_{L,hom}$	206.2 ±3.5	183.4 ±3.1	182.3 ±1.9	219.5 ±3.7	175.8 ±4.8
I_P	275.3 ±21.3	446.2 ±35.2	310.5 ±20.6	372.3 ±35.3	313.7 ±18.6
$I_{P,hom}$	19773 ±270	25360 ±565	20545 ±229	20602 ±607	18475 ±599
R_L	1.1 ±0.0	1.1 ±0.0	1.1 ±0.0	1.2 ±0.0	1.2 ±0.0
ΔG_{NET}	522.0 ±0.1	522.1 ±0.1	522.1 ±0.1	521.8 ±0.1	531.5 ±0.1
ΔG_{USV}	-516.6 ±0.1	-516.7 ±0.1	-516.7 ±0.1	-516.4 ±0.1	-526.1 ±0.1
ΔG_{RIP}	3.2 ±0.1	3.1 ±0.3	3.4 ±0.2	3.3 ±0.1	4.7 ±0.1
ΔG_{EMP}	0.0 ±0.0	0.0 ±0.0	0.0 ±0.0	0.0 ±0.0	-0.1 ±0.0
ΔG_{DSC}	64.5 ±0.1	64.4 ±0.1	64.5 ±0.0	64.4 ±0.0	64.4 ±0.0
ΔG_{corr}	73.0 ±0.1	72.8 ±0.3	73.2 ±0.1	73.0 ±0.2	74.6 ±0.2
Free ligand simulation					
N_s	1447	1759	1762	1583	1583
L	3.7 ±0.0	3.9 ±0.0	3.9 ±0.0	3.8 ±0.0	3.8 ±0.0
I_L	-7.3 ±0.1	-8.6 ±0.1	-8.5 ±0.1	-8.5 ±0.1	-8.5 ±0.1
$I_{L,hom}$	244.4 ±4.4	162.0 ±6.3	176.0 ±8.6	163.0 ±4.4	163.0 ±4.4
R_L	0.9 ±0.0	0.8 ±0.0	0.8 ±0.0	0.8 ±0.0	0.8 ±0.0
ΔG_{NET}	53.8 ±0.0	50.1 ±0.0	50.4 ±0.0	52.2 ±0.1	52.2 ±0.1
ΔG_{USV}	-53.3 ±0.0	-49.6 ±0.0	-49.9 ±0.0	-51.7 ±0.1	-51.7 ±0.1
ΔG_{RIP}	0.1 ±0.0	0.1 ±0.0	0.1 ±0.0	0.2 ±0.0	0.2 ±0.0
ΔG_{EMP}	-0.1 ±0.0	0.0 ±0.0	0.0 ±0.0	0.0 ±0.0	0.0 ±0.0
ΔG_{DSC}	65.5 ±0.2	64.3 ±0.1	65.7 ±0.1	65.5 ±0.2	65.5 ±0.2
ΔG_{corr}	66.1 ±0.2	65.0 ±0.1	66.3 ±0.1	66.2 ±0.2	66.2 ±0.2
Difference					
$\Delta\Delta G_{corr}$	6.9 ±0.2	7.9 ±0.3	6.9 ±0.2	6.8 ±0.2	8.4 ±0.3

Table S4. AMBER topology files for all the ligands, including atom types in the third column and RESP charges in the last column.

l10.in

```
    0    0    2

This is a remark line
molecule.res
L10   INT  0
CORRECT     OMIT DU   BEG
  0.0000
   1  DUMM  DU    M    0  -1  -2     0.000      .0        .0      .00000
   2  DUMM  DU    M    1   0  -1     1.449      .0        .0      .00000
   3  DUMM  DU    M    2   1   0     1.523   111.21       .0      .00000
   4  O1    o     M    3   2   1     1.540   111.208  -180.000 -0.799127
   5  C12   c     M    4   3   2     1.264   159.272   -59.651  0.821694
   6  O2    o     E    5   4   3     1.263   124.511     1.830 -0.799127
   7  C11   ca    M    5   4   3     1.523   117.689  -178.249 -0.024094
   8  C10   ca    B    7   5   4     1.399   120.491   179.341 -0.129285
   9  C9    ca    S    8   7   5     1.393   120.496   179.755 -0.238137
  10  H7    ha    E    9   8   7     1.099   120.154  -179.546  0.133050
  11  H8    ha    E    8   7   5     1.102   118.223     0.048  0.134937
  12  C8    ca    M    7   5   4     1.398   120.320    -0.769 -0.129285
  13  H6    ha    E   12   7   5     1.102   118.192    -0.034  0.134937
  14  C7    ca    M   12   7   5     1.393   120.507   179.957 -0.238137
  15  H5    ha    E   14  12   7     1.100   119.902   179.640  0.133050
  16  C6    ca    M   14  12   7     1.399   120.123     0.305  0.153221
  17  C5    c3    M   16  14  12     1.497   119.860   177.499 -0.166750
  18  H3    h1    E   17  16  14     1.131   109.404    -6.928  0.095767
  19  H4    h1    E   17  16  14     1.131   109.031  -125.187  0.095767
  20  N1    n     M   17  16  14     1.443   115.966   114.465 -0.282586
  21  C16   c     S   20  17  16     1.410   124.754   116.135  0.659490
  22  O3    o     E   21  20  17     1.237   124.438     5.609 -0.568148
  23  C4    ca    M   20  17  16     1.406   125.345   -56.800  0.265995
  24  C3    ca    M   23  20  17     1.396   129.398    -4.173 -0.250319
  25  H2    ha    E   24  23  20     1.101   120.793     3.753  0.160230
  26  C2    ca    M   24  23  20     1.397   118.314  -178.176  0.009175
  27  H1    ha    E   26  24  23     1.102   118.462  -179.502  0.128204
  28  C1    ca    M   26  24  23     1.396   121.377    -0.009 -0.155475
  29  Br1   br    E   28  26  24     1.874   120.001   179.375 -0.100931
  30  C13   ca    M   28  26  24     1.405   120.673    -0.879 -0.026861
  31  H9    ha    E   30  28  26     1.101   120.930  -178.991  0.140751
  32  C14   ca    M   30  28  26     1.379   118.669     0.792 -0.238133
  33  C15   c3    M   32  30  28     1.510   130.111  -179.295  0.134408
  34  C20   c3    3   33  32  30     1.523   111.312    62.519 -0.203555
  35  C19   c3    B   34  33  32     1.528   111.892  -175.618  0.066223
  36  H14   h1    E   35  34  33     1.131   108.031   -70.379  0.038061
  37  H15   h1    E   35  34  33     1.128   108.484   171.787  0.038061
  38  H16   hc    E   34  33  32     1.121   110.065    63.885  0.091187
  39  H17   hc    E   34  33  32     1.122   108.316   -53.927  0.091187
  40  C17   c3    M   33  32  30     1.522   111.668   -61.885 -0.203555
  41  H10   hc    E   40  33  32     1.121   108.403    54.063  0.091187
  42  H11   hc    E   40  33  32     1.120   110.110   -63.881  0.091187
  43  C18   c3    M   40  33  32     1.530   111.756   175.752  0.066223
  44  H12   h1    E   43  40  33     1.129   108.562  -172.925  0.038061
  45  H13   h1    E   43  40  33     1.132   107.850    69.373  0.038061
  46  N2    n3    M   43  40  33     1.442   113.649   -51.446 -0.225269
  47  S1    sy    M   46  43  40     1.622   123.115  -132.472  1.013730
  48  O4    o     E   47  46  43     1.413   110.507   144.270 -0.559084
  49  O5    o     E   47  46  43     1.415   109.593    15.560 -0.559084
  50  C21   cc    M   47  46  43     1.671   103.748  -100.265 -0.120400
  51  C22   cd    M   50  47  46     1.403   120.126  -110.308 -0.019187
  52  H18   ha    E   51  50  47     1.096   123.529     0.787  0.156828
  53  C23   cd    M   51  50  47     1.413   112.228  -178.752 -0.294942
  54  H19   ha    E   53  51  50     1.091   124.025  -179.928  0.184665
  55  C24   cc    M   53  51  50     1.391   110.744    -0.130 -0.105594
  56  H20   h4    E   55  53  51     1.090   124.290  -179.717  0.192697
  57  S2    ss    M   55  53  51     1.637   111.674     0.180  0.039032


LOOP
   C6   C9
  C15  C16
  C14   C4
   N2  C19
   S2  C21

IMPROPER
  C11   O1  C12   O2
  C12   C8  C11  C10
  C11   C9  C10   H8
   C6  C10   C9   H7
   C7  C11   C8   H6
   C8   C6   C7   H5
   C5   C7   C6   C9
  C16   C5   N1   C4
  C15   N1  C16   O3
   C3  C14   C4   N1
   C4   C2   C3   H2
   C3   C1   C2   H1
  Br1   C2   C1  C13
   C1  C14  C13   H9
  C15   C4  C14  C13
  C22   S2  C21   S1
  C21  C23  C22  H18
  C24  C22  C23  H19
  C23  H20  C24   S2

DONE
```

```
STOP
```

l12.in

```
    0    0    2

This is a remark line
molecule.res
L12   INT  0
CORRECT     OMIT DU   BEG
  0.0000
   1  DUMM  DU    M    0  -1  -2     0.000      .0        .0      .00000
   2  DUMM  DU    M    1   0  -1     1.449      .0        .0      .00000
   3  DUMM  DU    M    2   1   0     1.523   111.21       .0      .00000
   4  O4    o     M    3   2   1     1.540   111.208  -180.000 -0.799417
   5  C12   c     M    4   3   2     1.263     5.171   -38.581  0.820703
   6  O5    o     E    5   4   3     1.263   124.465    97.753 -0.799417
   7  C11   ca    M    5   4   3     1.524   117.763   -82.352 -0.026518
   8  C10   ca    B    7   5   4     1.399   120.485  -178.785 -0.124624
   9  C9    ca    S    8   7   5     1.392   120.518   179.858 -0.252181
  10  H7    ha    E    9   8   7     1.101   120.434  -179.617  0.138514
  11  H8    ha    E    8   7   5     1.102   118.147    -0.030  0.133784
  12  C8    ca    M    7   5   4     1.399   120.363     1.157 -0.124624
  13  H6    ha    E   12   7   5     1.103   118.159    -0.083  0.133784
  14  C7    ca    M   12   7   5     1.393   120.547   179.966 -0.252181
  15  H5    ha    E   14  12   7     1.099   120.010   179.948  0.138514
  16  C6    ca    M   14  12   7     1.399   120.092     0.251  0.162012
  17  C5    c3    M   16  14  12     1.496   120.397   178.027 -0.175420
  18  H3    h1    E   17  16  14     1.128   109.767   -12.889  0.104899
  19  H4    h1    E   17  16  14     1.131   108.951  -131.822  0.104899
  20  N1    n     M   17  16  14     1.444   115.840   108.505 -0.353619
  21  C16   c     S   20  17  16     1.414   124.098    89.846  0.677930
  22  O1    o     E   21  20  17     1.237   124.349    15.215 -0.560486
  23  C4    ca    M   20  17  16     1.408   124.351   -70.907  0.339885
  24  C3    ca    M   23  20  17     1.396   129.343   -14.185 -0.315369
  25  H2    ha    E   24  23  20     1.100   120.974     4.647  0.173737
  26  C2    ca    M   24  23  20     1.397   118.419  -176.825  0.035585
  27  H1    ha    E   26  24  23     1.103   118.461  -179.720  0.122112
  28  C1    ca    M   26  24  23     1.395   121.345    -0.117 -0.177263
  29  Br1   br    E   28  26  24     1.874   119.963   179.446 -0.096490
  30  C13   ca    M   28  26  24     1.405   120.703    -0.820 -0.006053
  31  H9    ha    E   30  28  26     1.101   120.932  -178.857  0.139921
  32  C14   ca    M   30  28  26     1.379   118.660     0.811 -0.256249
  33  C15   c3    M   32  30  28     1.509   130.082  -179.266  0.182014
  34  C20   c3    3   33  32  30     1.523   111.108    62.863 -0.266832
  35  C19   c3    B   34  33  32     1.529   111.880  -175.066  0.123949
  36  H14   h1    E   35  34  33     1.132   108.150   -69.808  0.035501
  37  H15   h1    E   35  34  33     1.127   108.580   172.193  0.035501
  38  H16   hc    E   34  33  32     1.121   110.078    64.525  0.102204
  39  H17   hc    E   34  33  32     1.121   108.406   -53.340  0.102204
  40  C17   c3    M   33  32  30     1.522   111.724   -61.786 -0.266832
  41  H10   hc    E   40  33  32     1.121   108.410    53.223  0.102204
  42  H11   hc    E   40  33  32     1.121   110.105   -64.682  0.102204
  43  C18   c3    M   40  33  32     1.530   111.814   175.054  0.123949
  44  H12   h1    E   43  40  33     1.129   108.894  -173.307  0.035501
  45  H13   h1    E   43  40  33     1.131   108.105    68.801  0.035501
  46  N2    n3    M   43  40  33     1.443   113.351   -51.769 -0.283152
  47  S1    sy    M   46  43  40     1.614   123.654  -126.938  1.059963
  48  O2    o     E   47  46  43     1.416   110.560   135.848 -0.582353
  49  O3    o     E   47  46  43     1.411   111.077     7.240 -0.582353
  50  C21   ca    M   47  46  43     1.714   103.657  -110.444  0.009944
  51  C22   ca    M   50  47  46     1.404   117.056  -114.792 -0.145324
  52  H18   ha    E   51  50  47     1.107   118.905     1.196  0.177001
  53  C23   ca    M   51  50  47     1.391   121.686  -178.797 -0.157070
  54  H19   ha    E   53  51  50     1.100   120.012  -179.799  0.145491
  55  C24   ca    M   53  51  50     1.395   119.790    -0.064 -0.103708
  56  H20   ha    E   55  53  51     1.101   120.267  -179.686  0.143630
  57  C26   ca    M   55  53  51     1.392   119.901     0.033 -0.106287
  58  H21   ha    E   57  55  53     1.101   120.174  -179.764  0.134829
  59  C25   ca    M   57  55  53     1.399   119.703    -0.054  0.031165
  60  Cl1   cl    M   59  57  55     1.700   117.744  -179.731 -0.095211


LOOP
   C6   C9
  C15  C16
  C14   C4
   N2  C19
  C25  C21

IMPROPER
  C11   O4  C12   O5
  C12   C8  C11  C10
  C11   C9  C10   H8
   C6  C10   C9   H7
   C7  C11   C8   H6
   C8   C6   C7   H5
   C5   C7   C6   C9
  C16   C5   N1   C4
  C15   N1  C16   O1
   C3  C14   C4   N1
   C4   C2   C3   H2
   C3   C1   C2   H1
  Br1   C2   C1  C13
   C1  C14  C13   H9
  C15   C4  C14  C13
  C22  C25  C21   S1
  C21  C23  C22  H18
  C22  C24  C23  H19
  C23  C26  C24  H20
  C24  C25  C26  H21
  C21  C26  C25  Cl1
```

```
DONE
STOP
```

l17.in

```
    0    0    2

This is a remark line
molecule.res
L17   INT  0
CORRECT     OMIT DU   BEG
  0.0000
   1  DUMM  DU    M    0  -1  -2     0.000      .0        .0      .00000
   2  DUMM  DU    M    1   0  -1     1.449      .0        .0      .00000
   3  DUMM  DU    M    2   1   0     1.523   111.21       .0      .00000
   4  C23   c3    M    3   2   1     1.540   111.208  -180.000 -0.348124
   5  H16   hc    E    4   3   2     1.116   102.576  -174.944  0.086719
   6  H17   hc    E    4   3   2     1.117   148.270     2.147  0.086719
   7  H18   hc    E    4   3   2     1.117    57.620   -71.065  0.086719
   8  C22   c3    M    4   3   2     1.508    59.350    78.352  0.453529
   9  H14   h1    E    8   4   3     1.121   112.024   151.176 -0.017110
  10  H15   h1    E    8   4   3     1.121   112.022   -85.919 -0.017110
  11  O5    os    M    8   4   3     1.439   106.212    32.639 -0.535070
  12  C7    c     M   11   8   4     1.371   116.187  -179.727  0.867770
  13  O4    o     E   12  11   8     1.235   117.804    -0.211 -0.630709
  14  C11   ca    M   12  11   8     1.467   114.261   179.791 -0.256484
  15  C17   ca    B   14  12  11     1.400   118.779  -179.558 -0.005771
  16  C19   ca    S   15  14  12     1.390   120.772  -179.939 -0.340940
  17  H11   ha    E   16  15  14     1.103   118.963  -179.808  0.187698
  18  H9    ha    E   15  14  12     1.103   119.306     0.095  0.138308
  19  C16   ca    M   14  12  11     1.399   121.709     0.394 -0.005771
  20  H8    ha    E   19  14  12     1.103   119.577    -0.107  0.138308
  21  C18   ca    M   19  14  12     1.387   120.435   179.903 -0.340940
  22  H10   ha    E   21  19  14     1.101   119.004   179.894  0.187698
  23  C15   ca    M   21  19  14     1.420   120.491     0.056  0.439734
  24  N4    n     M   23  21  19     1.402   118.011  -179.582 -0.704839
  25  H3    hn    E   24  23  21     0.998   115.570     0.352  0.327156
  26  C3    c     M   24  23  21     1.388   127.277  -176.657  1.016763
  27  O3    o     E   26  24  23     1.242   124.155    -0.806 -0.590629
  28  C1    cc    M   26  24  23     1.485   114.985   177.860 -0.542642
  29  N1    na    B   28  26  24     1.416   125.670    55.409  0.306150
  30  N2    nc    E   29  28  26     1.347   111.640  -179.371 -0.557865
  31  C9    ca    S   29  28  26     1.428   125.011    10.890  0.227430
  32  C20   ca    B   31  29  28     1.409   120.336    34.068 -0.242189
  33  C25   ca    B   32  31  29     1.392   119.655  -179.198 -0.083007
  34  C26   ca    B   33  32  31     1.395   120.570    -0.815 -0.184676
  35  C24   ca    B   34  33  32     1.393   119.799    -0.050 -0.083007
  36  C21   ca    S   35  34  33     1.393   120.685     0.445 -0.242189
  37  H13   ha    E   36  35  34     1.102   119.747  -179.336  0.156935
  38  H19   ha    E   35  34  33     1.101   120.014  -179.477  0.140064
  39  H21   ha    E   34  33  32     1.100   120.052  -179.799  0.146526
  40  H20   ha    E   33  32  31     1.101   119.369   179.582  0.140064
  41  H12   ha    E   32  31  29     1.103   120.638     0.915  0.156935
  42  C2    cd    M   28  26  24     1.397   127.974  -122.304  0.205777
  43  C6    c3    B   42  28  26     1.480   132.348    -2.081 -0.350153
  44  H1    h1    E   43  42  28     1.132   108.163   -69.953  0.142998
  45  H2    h1    E   43  42  28     1.132   109.685    47.567  0.142998
  46  C4    cd    M   42  28  26     1.455   104.984   178.517  0.294551
  47  C8    c3    M   46  42  28     1.478   121.137  -178.406 -0.130254
  48  H4    hc    E   47  46  42     1.123   109.344   103.819  0.096623
  49  H5    hc    E   47  46  42     1.121   111.240  -137.258  0.096623
  50  C10   c3    M   47  46  42     1.538   109.826   -17.188 -0.036401
  51  H6    h1    E   50  47  46     1.128   107.903   165.905  0.065640
  52  H7    h1    E   50  47  46     1.128   108.003   -77.111  0.065640
  53  N3    n3    M   50  47  46     1.442   114.833    44.369 -0.158458
  54  S1    sy    M   53  50  47     1.636   122.747   118.079  1.065353
  55  O1    o     E   54  53  50     1.411   109.608  -142.389 -0.553937
  56  O2    o     E   54  53  50     1.412   108.938   -13.753 -0.553937
  57  C5    cc    M   54  53  50     1.663   103.537   102.036 -0.123280
  58  C12   cd    M   57  54  53     1.405   120.216   101.537 -0.033056
  59  H22   ha    E   58  57  54     1.097   123.560    -0.688  0.154049
  60  C14   cd    M   58  57  54     1.413   112.273   178.270 -0.259150
  61  H24   ha    E   60  58  57     1.092   124.048  -179.918  0.185916
  62  C13   cc    M   60  58  57     1.392   110.743     0.027 -0.136712
  63  H23   h4    E   62  60  58     1.091   124.231  -179.950  0.207149
  64  S2    ss    M   62  60  58     1.637   111.714    -0.275  0.049866


LOOP
  C15  C19
   C4   N2
  C21   C9
   N3   C6
   S2   C5

IMPROPER
  C11   O4   C7   O5
   C7  C17  C11  C16
  C11  C19  C17   H9
  C17  C15  C19  H11
  C11  C18  C16   H8
  C16  C15  C18  H10
  C19  C18  C15   N4
   C3  C15   N4   H3
   C1   N4   C3   O3
   C3   C2   C1   N1
   C9   C1   N1   N2
  C20  C21   C9   N1
   C9  C25  C20  H12
  C20  C26  C25  H20
  C25  C24  C26  H21
  C26  C21  C24  H19
   C9  C24  C21  H13
```

```
   C6   C1   C2   C4
   C8   C2   C4   N2
  C12   S2   C5   S1
   C5  C14  C12  H22
  C13  C12  C14  H24
  C14  H23  C13   S2

DONE
STOP
```

l38.in

```
    0    0    2

This is a remark line
molecule.res
L38   INT  0
CORRECT     OMIT DU   BEG
  0.0000
   1  DUMM  DU    M    0  -1  -2     0.000      .0        .0      .00000
   2  DUMM  DU    M    1   0  -1     1.449      .0        .0      .00000
   3  DUMM  DU    M    2   1   0     1.523   111.21       .0      .00000
   4  O4    o     M    3   2   1     1.540   111.208  -180.000 -0.798993
   5  C12   c     M    4   3   2     1.263    76.212   121.837  0.821226
   6  O5    o     E    5   4   3     1.262   124.576    -0.670 -0.798993
   7  C11   ca    M    5   4   3     1.524   117.714   179.379 -0.023670
   8  C10   ca    B    7   5   4     1.398   120.534    -0.703 -0.129539
   9  C9    ca    S    8   7   5     1.394   120.531   179.794 -0.237670
  10  H7    ha    E    9   8   7     1.099   120.150  -179.501  0.132938
  11  H8    ha    E    8   7   5     1.102   118.267     0.008  0.134987
  12  C8    ca    M    7   5   4     1.399   120.319   179.203 -0.129539
  13  H6    ha    E   12   7   5     1.102   118.148    -0.055  0.134987
  14  C7    ca    M   12   7   5     1.392   120.551   179.956 -0.237670
  15  H5    ha    E   14  12   7     1.100   119.919   179.634  0.132938
  16  C6    ca    M   14  12   7     1.400   120.132     0.291  0.152406
  17  C5    c3    M   16  14  12     1.497   119.865   177.477 -0.166121
  18  H3    h1    E   17  16  14     1.131   108.996  -125.216  0.095677
  19  H4    h1    E   17  16  14     1.130   109.404    -6.926  0.095677
  20  N1    n     M   17  16  14     1.443   115.897   114.473 -0.282701
  21  C16   c     S   20  17  16     1.410   124.709   116.272  0.659685
  22  O1    o     E   21  20  17     1.237   124.511     5.466 -0.568252
  23  C4    ca    M   20  17  16     1.406   125.373   -56.815  0.266085
  24  C3    ca    M   23  20  17     1.396   129.476    -4.050 -0.250707
  25  H2    ha    E   24  23  20     1.103   120.711     3.662  0.160377
  26  C2    ca    M   24  23  20     1.397   118.389  -178.183  0.009660
  27  H1    ha    E   26  24  23     1.103   118.388  -179.539  0.128076
  28  C1    ca    M   26  24  23     1.395   121.352     0.020 -0.155908
  29  Br1   br    E   28  26  24     1.874   119.986   179.416 -0.100832
  30  C13   ca    M   28  26  24     1.405   120.691    -0.977 -0.026938
  31  H9    ha    E   30  28  26     1.101   120.894  -178.927  0.140800
  32  C14   ca    M   30  28  26     1.378   118.715     0.896 -0.237566
  33  C15   c3    M   32  30  28     1.510   130.162  -179.354  0.133786
  34  C20   c3    3   33  32  30     1.522   111.308    62.522 -0.204166
  35  C19   c3    B   34  33  32     1.529   111.891  -175.654  0.066765
  36  H14   h1    E   35  34  33     1.128   108.495   171.753  0.037940
  37  H15   h1    E   35  34  33     1.131   108.016   -70.384  0.037940
  38  H16   hc    E   34  33  32     1.122   110.078    63.886  0.091345
  39  H17   hc    E   34  33  32     1.122   108.345   -53.909  0.091345
  40  C17   c3    M   33  32  30     1.521   111.666   -61.913 -0.204166
  41  H10   hc    E   40  33  32     1.122   108.362    54.082  0.091345
  42  H11   hc    E   40  33  32     1.120   110.166   -63.847  0.091345
  43  C18   c3    M   40  33  32     1.531   111.765   175.740  0.066765
  44  H12   h1    E   43  40  33     1.128   108.536  -172.917  0.037940
  45  H13   h1    E   43  40  33     1.132   107.786    69.425  0.037940
  46  N2    n3    M   43  40  33     1.442   113.629   -51.405 -0.224701
  47  S1    sy    M   46  43  40     1.621   123.096  -132.514  1.012436
  48  O2    o     E   47  46  43     1.412   110.519   144.273 -0.558830
  49  O3    o     E   47  46  43     1.416   109.599    15.594 -0.558830
  50  C21   cc    M   47  46  43     1.671   103.723  -100.232 -0.120139
  51  C22   cd    M   50  47  46     1.404   120.094  -110.499 -0.018273
  52  H18   ha    E   51  50  47     1.097   123.513     0.881  0.156516
  53  C23   cd    M   51  50  47     1.414   112.204  -178.744 -0.295962
  54  H19   ha    E   53  51  50     1.092   123.973  -179.830  0.185006
  55  C24   cc    M   53  51  50     1.391   110.768    -0.049 -0.105705
  56  H20   h4    E   55  53  51     1.090   124.319  -179.678  0.192913
  57  S2    ss    M   55  53  51     1.637   111.689     0.104  0.039026


LOOP
   C6   C9
  C15  C16
  C14   C4
   N2  C19
   S2  C21

IMPROPER
  C11   O4  C12   O5
  C12   C8  C11  C10
  C11   C9  C10   H8
   C6  C10   C9   H7
   C7  C11   C8   H6
   C8   C6   C7   H5
   C5   C7   C6   C9
  C16   C5   N1   C4
  C15   N1  C16   O1
   C3  C14   C4   N1
   C4   C2   C3   H2
   C3   C1   C2   H1
  Br1   C2   C1  C13
   C1  C14  C13   H9
  C15   C4  C14  C13
  C22   S2  C21   S1
  C21  C23  C22  H18
```

```
  C24  C22  C23  H19
  C23  H20  C24   S2

DONE
STOP
```

l41.in

```
    0    0    2

This is a remark line
molecule.res
L41   INT  0
CORRECT     OMIT DU   BEG
  0.0000
   1  DUMM  DU    M    0  -1  -2     0.000      .0        .0      .00000
   2  DUMM  DU    M    1   0  -1     1.449      .0        .0      .00000
   3  DUMM  DU    M    2   1   0     1.523   111.21       .0      .00000
   4  C27   c3    M    3   2   1     1.540   111.208  -180.000 -0.053651
   5  H22   h1    E    4   3   2     1.117    89.795   -22.707  0.094070
   6  H23   h1    E    4   3   2     1.117   155.641  -174.772  0.094070
   7  H24   h1    E    4   3   2     1.117    69.069    88.847  0.094070
   8  O5    os    M    4   3   2     1.428    56.228  -137.116 -0.437933
   9  C12   c     M    8   4   3     1.371   116.430    15.032  0.889394
  10  O4    o     E    9   8   4     1.235   117.647     0.002 -0.602400
  11  C11   ca    M    9   8   4     1.469   114.369  -179.955 -0.271276
  12  C10   ca    B   11   9   8     1.403   118.462  -179.547 -0.026872
  13  C9    ca    S   12  11   9     1.392   119.811   179.992 -0.239882
  14  H7    ha    E   13  12  11     1.103   120.322  -179.934  0.154792
  15  H8    ha    E   12  11   9     1.102   119.422     0.071  0.128007
  16  C8    ca    M   11   9   8     1.400   121.426     0.555 -0.026872
  17  H6    ha    E   16  11   9     1.101   119.752    -0.041  0.128007
  18  C7    ca    M   16  11   9     1.394   119.791   179.944 -0.239882
  19  H5    ha    E   18  16  11     1.101   119.636  -179.816  0.154792
  20  C6    ca    M   18  16  11     1.397   120.206     0.143  0.247249
  21  C5    c3    M   20  18  16     1.504   120.416   178.208 -0.341516
  22  H3    h1    E   21  20  18     1.129   109.501   -12.272  0.163885
  23  H4    h1    E   21  20  18     1.132   108.140  -130.494  0.163885
  24  N1    n     M   21  20  18     1.435   115.307   109.774 -0.242433
  25  C16   c     S   24  21  20     1.416   124.641    85.134  0.626632
  26  O1    o     E   25  24  21     1.236   123.515     6.536 -0.530226
  27  C4    ca    M   24  21  20     1.408   125.023   -84.054  0.268884
  28  C3    ca    M   27  24  21     1.394   129.515    -7.360 -0.321157
  29  H2    ha    E   28  27  24     1.098   121.438     1.838  0.168566
  30  C2    ca    M   28  27  24     1.399   118.311  -178.379  0.043896
  31  H1    ha    E   30  28  27     1.103   118.439   179.881  0.120363
  32  C1    ca    M   30  28  27     1.395   121.381    -0.169 -0.177012
  33  Br1   br    E   32  30  28     1.871   119.983   179.981 -0.067035
  34  C13   ca    M   32  30  28     1.406   120.597    -0.098 -0.007922
  35  H9    ha    E   34  32  30     1.102   120.856  -179.823  0.146735
  36  C14   ca    M   34  32  30     1.378   118.807     0.125 -0.223494
  37  C15   c3    M   36  34  32     1.511   130.089  -179.992  0.150092
  38  C20   c3    3   37  36  34     1.524   111.333    61.517 -0.232162
  39  C19   c3    B   38  37  36     1.530   111.979  -174.087  0.184866
  40  H14   h1    E   39  38  37     1.131   108.259   -69.929  0.011895
  41  H15   h1    E   39  38  37     1.127   108.409   172.192  0.011895
  42  H16   hc    E   38  37  36     1.120   110.107    65.283  0.095291
  43  H17   hc    E   38  37  36     1.122   108.251   -52.472  0.095291
  44  C17   c3    M   37  36  34     1.524   111.336   -63.208 -0.232162
  45  H10   hc    E   44  37  36     1.122   108.248    52.627  0.095291
  46  H11   hc    E   44  37  36     1.121   110.060   -65.072  0.095291
  47  C18   c3    M   44  37  36     1.531   111.901   174.375  0.184866
  48  H12   h1    E   47  44  37     1.129   108.718  -173.204  0.011895
  49  H13   h1    E   47  44  37     1.131   108.145    69.033  0.011895
  50  N2    n3    M   47  44  37     1.441   113.294   -51.650 -0.347429
  51  S1    sy    M   50  47  44     1.623   123.616  -122.896  1.100484
  52  O2    o     E   51  50  47     1.414   109.696   136.064 -0.579870
  53  O3    o     E   51  50  47     1.408   110.510     7.642 -0.579870
  54  C21   ca    M   51  50  47     1.709   103.721  -110.130 -0.002153
  55  C22   ca    M   54  51  50     1.405   116.945  -116.905 -0.138299
  56  H18   ha    E   55  54  51     1.107   119.009     1.695  0.174009
  57  C23   ca    M   55  54  51     1.391   121.658  -177.847 -0.160796
  58  H19   ha    E   57  55  54     1.100   120.021   179.833  0.149264
  59  C24   ca    M   57  55  54     1.395   119.801    -0.143 -0.095633
  60  H20   ha    E   59  57  55     1.101   120.216   179.924  0.146759
  61  C26   ca    M   59  57  55     1.392   119.908    -0.004 -0.109167
  62  H21   ha    E   61  59  57     1.101   120.146   179.952  0.137973
  63  C25   ca    M   61  59  57     1.399   119.684     0.023  0.030954
  64  Cl1   cl    M   63  61  59     1.699   117.747  -179.932 -0.088209


LOOP
   C6   C9
  C15  C16
  C14   C4
   N2  C19
  C25  C21

IMPROPER
  C11   O4  C12   O5
  C12  C10  C11   C8
  C11   C9  C10   H8
  C10   C6   C9   H7
  C11   C7   C8   H6
   C8   C6   C7   H5
   C5   C9   C6   C7
  C16   C5   N1   C4
  C15   N1  C16   O1
   C3  C14   C4   N1
   C4   C2   C3   H2
   C3   C1   C2   H1
  Br1   C2   C1  C13
   C1  C14  C13   H9
```

```
  C15   C4  C14  C13
  C22  C25  C21   S1
  C21  C23  C22  H18
  C22  C24  C23  H19
  C23  C26  C24  H20
  C24  C25  C26  H21
  C21  C26  C25  C11

DONE
STOP
```

l45.in

```
    0    0    2

This is a remark line
molecule.res
L45   INT  0
CORRECT     OMIT DU   BEG
  0.0000
   1  DUMM  DU    M    0  -1  -2     0.000      .0        .0       .00000
   2  DUMM  DU    M    1   0  -1     1.449      .0        .0       .00000
   3  DUMM  DU    M    2   1   0     1.523   111.21       .0       .00000
   4  C26   c3    M    3   2   1     1.540   111.208  -180.000 -0.409566
   5  H21   hc    E    4   3   2     1.116   108.870  -127.228  0.105985
   6  H22   hc    E    4   3   2     1.117    33.616   -30.630  0.105985
   7  H23   hc    E    4   3   2     1.117    78.060   127.300  0.105985
   8  C25   c3    M    4   3   2     1.511   133.978    20.235  0.482658
   9  H16   h1    E    8   4   3     1.121   111.463   -88.715 -0.043832
  10  H17   h1    E    8   4   3     1.122   111.375    33.764 -0.043832
  11  O5    os    M    8   4   3     1.434   106.230   152.233 -0.491327
  12  C7    c     M   11   8   4     1.373   117.410   174.308  0.822762
  13  O4    o     E   12  11   8     1.232   112.343  -175.974 -0.586673
  14  C11   ca    M   12  11   8     1.475   120.815     4.037 -0.230244
  15  C17   ca    B   14  12  11     1.396   120.785    71.896 -0.054897
  16  C19   ca    S   15  14  12     1.391   120.813   177.678 -0.349466
  17  H11   ha    E   16  15  14     1.102   118.970  -179.814  0.194193
  18  H9    ha    E   15  14  12     1.102   119.940    -1.702  0.161897
  19  C16   ca    M   14  12  11     1.399   119.613  -111.074 -0.054897
  20  H8    ha    E   19  14  12     1.102   119.892     1.541  0.161897
  21  C18   ca    M   19  14  12     1.388   120.437  -177.981 -0.349466
  22  H10   ha    E   21  19  14     1.102   119.024  -179.921  0.194193
  23  C15   ca    M   21  19  14     1.419   120.393     0.562  0.493533
  24  N4    n     M   23  21  19     1.403   117.986  -179.920 -0.764979
  25  H3    hn    E   24  23  21     1.000   115.627     0.843  0.386523
  26  C3    c     M   24  23  21     1.387   127.337  -176.240  0.958668
  27  O3    o     E   26  24  23     1.243   124.162    -3.048 -0.558699
  28  C1    cc    M   26  24  23     1.485   113.792   175.819 -0.543972
  29  C2    cd    M   28  26  24     1.400   127.944   -57.320  0.123713
  30  C6    c3    3   29  28  26     1.480   132.837    -2.716 -0.124519
  31  N3    n3    B   30  29  28     1.446   111.989   164.385 -0.327707
  32  S1    sy    3   31  30  29     1.638   121.388  -128.476  1.127761
  33  C5    cc    S   32  31  30     1.659   103.609   -86.216 -0.144771
  34  C12   cd    B   33  32  31     1.406   120.271   101.413 -0.057787
  35  C14   cd    B   34  33  32     1.412   112.277   178.314 -0.222787
  36  C13   cc    B   35  34  33     1.392   110.736    -0.097 -0.157723
  37  S2    ss    E   36  35  34     1.636   111.782    -0.218  0.073726
  38  H19   h4    E   36  35  34     1.091   124.193  -179.795  0.212797
  39  H20   ha    E   35  34  33     1.092   124.064  -179.884  0.179400
  40  H18   ha    E   34  33  32     1.096   123.551    -0.715  0.153399
  41  O1    o     E   32  31  30     1.414   108.951    29.209 -0.559356
  42  O2    o     E   32  31  30     1.411   109.060   157.697 -0.559356
  43  C10   c3    3   31  30  29     1.441   115.073    45.018  0.003201
  44  C8    c3    B   43  31  30     1.539   115.031   -58.806 -0.119858
  45  H4    hc    E   44  43  31     1.123   109.960   -80.351  0.093950
  46  H5    hc    E   44  43  31     1.122   108.418   162.068  0.093950
  47  H6    h1    E   43  31  30     1.129   108.691  -179.897  0.067948
  48  H7    h1    E   43  31  30     1.129   108.573    62.417  0.067948
  49  H1    h1    E   30  29  28     1.132   108.399   -75.048  0.097249
  50  H2    h1    E   30  29  28     1.131   110.331    43.026  0.097249
  51  C4    cd    M   29  28  26     1.452   105.117   179.554  0.292435
  52  N2    nc    M   51  29  28     1.360   109.952     0.114 -0.570278
  53  N1    na    M   52  51  29     1.344   106.963     0.740  0.395402
  54  C9    ca    M   53  52  51     1.430   121.863  -176.583  0.069978
  55  C21   ca    B   54  53  52     1.408   119.754    47.637 -0.165759
  56  C22   ca    S   55  54  53     1.392   120.051  -179.217 -0.235138
  57  H14   ha    E   56  55  54     1.100   119.958   179.902  0.179509
  58  H13   ha    E   55  54  53     1.103   120.876     0.443  0.176309
  59  C20   ca    M   54  53  52     1.411   120.258  -130.557 -0.165759
  60  H12   ha    E   59  54  53     1.105   120.609    -1.545  0.176309
  61  C23   ca    M   59  54  53     1.389   120.037   179.238 -0.235138
  62  H15   ha    E   61  59  54     1.100   120.798   179.445  0.179509
  63  C24   ca    M   61  59  54     1.404   119.283    -0.348  0.321648
  64  O6    os    M   63  61  59     1.391   114.191   177.095 -0.383961
  65  C27   c3    M   64  63  61     1.403   119.921   160.419  0.792306
  66  F2    f     E   65  64  63     1.357   111.459    73.287 -0.212742
  67  F3    f     E   65  64  63     1.356   112.984   -49.983 -0.212742
  68  F1    f     M   65  64  63     1.355   104.493  -168.949 -0.212742


LOOP
  C15  C19
   N1   C1
   S2   C5
   C4   C8
  C24  C22

IMPROPER
  C11   O4   C7   O5
   C7  C17  C11  C16
  C11  C19  C17   H9
```

```
  C17  C15  C19  H11
  C11  C18  C16   H8
  C16  C15  C18  H10
  C19  C18  C15   N4
   C3  C15   N4   H3
   C1   N4   C3   O3
   C3   C2   C1   N1
   C6   C1   C2   C4
  C12   S2   C5   S1
   C5  C14  C12  H18
  C13  C12  C14  H20
  C14  H19  C13   S2
   C8   C2   C4   N2
   C9   C1   N1   N2
  C21  C20   C9   N1
   C9  C22  C21  H13
  C21  C24  C22  H14
   C9  C23  C20  H12
  C20  C24  C23  H15
  C22  C23  C24   O6

DONE
STOP

l46.in
    0    0    2

This is a remark line
molecule.res
L46   INT  0
CORRECT     OMIT DU   BEG
  0.0000
   1  DUMM  DU    M    0  -1  -2     0.000      .0        .0      .00000
   2  DUMM  DU    M    1   0  -1     1.449      .0        .0      .00000
   3  DUMM  DU    M    2   1   0     1.523   111.21       .0      .00000
   4  O4    o     M    3   2   1     1.540   111.208  -180.000 -0.631381
   5  C7    c     M    4   3   2     1.249    61.614  -104.542  0.924072
   6  N5    n     B    5   4   3     1.376   119.687   129.863 -1.099464
   7  H17   hn    E    6   5   4     0.986   121.019  -173.009  0.444101
   8  H18   hn    E    6   5   4     0.989   119.196    -2.826  0.444101
   9  C11   ca    M    5   4   3     1.488   121.925   -49.865 -0.267580
  10  C17   ca    B    9   5   4     1.398   118.360    33.032 -0.010085
  11  C19   ca    S   10   9   5     1.391   120.958  -179.730 -0.325034
  12  H11   ha    E   11  10   9     1.103   118.986  -179.483  0.185256
  13  H9    ha    E   10   9   5     1.103   119.165    -0.236  0.125256
  14  C16   ca    M    9   5   4     1.399   122.338  -145.587 -0.010085
  15  H8    ha    E   14   9   5     1.101   120.661     0.374  0.125256
  16  C18   ca    M   14   9   5     1.388   120.505   179.307 -0.325034
  17  H10   ha    E   16  14   9     1.101   118.939  -179.786  0.185256
  18  C15   ca    M   16  14   9     1.418   120.570     0.093  0.408017
  19  N4    n     M   18  16  14     1.403   118.117  -179.738 -0.700937
  20  H3    hn    E   19  18  16     0.998   115.605     3.106  0.319871
  21  C3    c     M   19  18  16     1.388   127.115  -173.010  1.041458
  22  O3    o     E   21  19  18     1.243   124.164    -1.003 -0.591919
  23  C1    cc    M   21  19  18     1.484   114.966   177.610 -0.566196
  24  N1    na    B   23  21  19     1.417   125.677    56.025  0.327793
  25  N2    nc    E   24  23  21     1.346   111.668  -179.234 -0.567456
  26  C9    ca    S   24  23  21     1.428   124.960    10.858  0.218298
  27  C20   ca    B   26  24  23     1.409   120.301    33.880 -0.237804
  28  C23   ca    B   27  26  24     1.393   119.603  -179.195 -0.082939
  29  C24   ca    B   28  27  26     1.395   120.583    -0.780 -0.186265
  30  C22   ca    B   29  28  27     1.395   119.801    -0.111 -0.082939
  31  C21   ca    S   30  29  28     1.393   120.656     0.498 -0.237804
  32  H13   ha    E   31  30  29     1.102   119.719  -179.396  0.154742
  33  H14   ha    E   30  29  28     1.100   119.999  -179.466  0.140285
  34  H16   ha    E   29  28  27     1.100   120.084  -179.814  0.146445
  35  H15   ha    E   28  27  26     1.101   119.355   179.463  0.140285
  36  H12   ha    E   27  26  24     1.103   120.744     0.937  0.154742
  37  C2    cd    M   23  21  19     1.398   128.008  -121.511  0.201274
  38  C6    c3    B   37  23  21     1.480   132.273    -2.299 -0.336590
  39  H1    h1    E   38  37  23     1.132   108.179   -70.275  0.137312
  40  H2    h1    E   38  37  23     1.131   109.738    47.426  0.137312
  41  C4    cd    M   37  23  21     1.454   104.996   178.384  0.297291
  42  C8    c3    M   41  37  23     1.478   121.161  -178.370 -0.116580
  43  H4    hc    E   42  41  37     1.123   109.321   103.923  0.093816
  44  H5    hc    E   42  41  37     1.122   111.247  -137.109  0.093816
  45  C10   c3    M   42  41  37     1.538   109.830   -17.048 -0.062836
  46  H6    h1    E   45  42  41     1.128   107.917   165.765  0.072722
  47  H7    h1    E   45  42  41     1.129   107.948   -77.304  0.072722
  48  N3    n3    M   45  42  41     1.442   114.890    44.130 -0.152935
  49  S1    sy    M   48  45  42     1.636   122.732   118.208  1.064543
  50  O1    o     E   49  48  45     1.411   109.601  -143.018 -0.554292
  51  O2    o     E   49  48  45     1.413   108.976   -14.403 -0.554292
  52  C5    cc    M   49  48  45     1.662   103.522   101.400 -0.123664
  53  C12   cd    M   52  49  48     1.405   120.219   101.212 -0.034979
  54  H19   ha    E   53  52  49     1.096   123.599    -0.710  0.154681
  55  C14   cd    M   53  52  49     1.413   112.273   178.285 -0.256760
  56  H21   ha    E   55  53  52     1.092   124.055  -179.936  0.185040
  57  C13   cc    M   55  53  52     1.392   110.733    -0.072 -0.137724
  58  H20   h4    E   57  55  53     1.091   124.255  -179.985  0.207338
  59  S2    ss    M   57  55  53     1.636   111.727    -0.189  0.050472


LOOP
  C15  C19
   C4   N2
  C21   C9
   N3   C6
   S2   C5

IMPROPER
  C11   N5   C7   O4
```

```
   C7  H17   N5  H18
   C7  C16  C11  C17
  C19  C11  C17   H9
  C15  C17  C19  H11
  C18  C11  C16   H8
  C16  C15  C18  H10
  C19  C18  C15   N4
   C3  C15   N4   H3
   C1   N4   C3   O3
   C3   C2   C1   N1
   C9   C1   N1   N2
  C20  C21   C9   N1
   C9  C23  C20  H12
  C20  C24  C23  H15
  C23  C22  C24  H16
  C24  C21  C22  H14
   C9  C22  C21  H13
   C6   C1   C2   C4
   C8   C2   C4   N2
  C12   S2   C5   S1
   C5  C14  C12  H19
  C13  C12  C14  H21
  C14  H20  C13   S2

DONE
STOP
```

l47.in

```
    0    0    2

This is a remark line
molecule.res
L47   INT  0
CORRECT     OMIT DU   BEG
  0.0000
   1  DUMM  DU    M    0  -1  -2     0.000      .0        .0      .00000
   2  DUMM  DU    M    1   0  -1     1.449      .0        .0      .00000
   3  DUMM  DU    M    2   1   0     1.523   111.21       .0      .00000
   4  C26   c3    M    3   2   1     1.540   111.208  -180.000  0.067631
   5  H19   h1    E    4   3   2     1.120   112.911    99.316  0.050350
   6  H20   h1    E    4   3   2     1.118    23.757     9.588  0.050350
   7  H21   h1    E    4   3   2     1.118   126.815   -42.872  0.050350
   8  O5    os    M    4   3   2     1.423    88.107  -156.500 -0.417542
   9  C25   c     M    8   4   3     1.372   118.062    55.482  0.810020
  10  O4    o     E    9   8   4     1.232   112.197  -178.734 -0.615561
  11  C24   c3    M    9   8   4     1.502   121.784     1.774 -0.317534
  12  H17   hc    E   11   9   8     1.122   109.862    37.733  0.111276
  13  H18   hc    E   11   9   8     1.127   107.569   154.496  0.111276
  14  C15   ca    M   11   9   8     1.490   111.813   -85.890  0.264545
  15  C17   ca    S   14  11   9     1.395   119.428   -54.594 -0.437052
  16  H9    ha    E   15  14  11     1.103   118.492     1.594  0.206036
  17  C10   ca    M   14  11   9     1.398   120.385   126.694 -0.300695
  18  H16   ha    E   17  14  11     1.100   120.311    -1.085  0.146292
  19  C16   ca    M   17  14  11     1.393   119.523   178.506 -0.044290
  20  H8    ha    E   19  17  14     1.101   119.737  -179.608  0.136540
  21  C18   ca    M   19  17  14     1.392   121.151     0.209 -0.373981
  22  H10   ha    E   21  19  17     1.102   119.284   179.871  0.230404
  23  C14   ca    M   21  19  17     1.412   119.964     0.089  0.461785
  24  N4    n     M   23  21  19     1.405   123.309  -179.462 -0.709882
  25  H3    hn    E   24  23  21     0.999   115.241   170.780  0.351682
  26  C3    c     M   24  23  21     1.386   126.748   -13.731  0.936655
  27  O3    o     E   26  24  23     1.246   123.410     2.264 -0.612627
  28  C1    cc    M   26  24  23     1.481   116.822   179.695 -0.460378
  29  N1    na    B   28  26  24     1.417   127.848    34.578  0.217289
  30  N2    nc    E   29  28  26     1.344   111.819  -178.482 -0.547325
  31  C8    ca    S   29  28  26     1.429   125.477     9.853  0.285329
  32  C19   ca    B   31  29  28     1.409   120.318    42.970 -0.262595
  33  C22   ca    B   32  31  29     1.392   119.576  -179.333 -0.091456
  34  C23   ca    B   33  32  31     1.395   120.551    -0.801 -0.144073
  35  C21   ca    B   34  33  32     1.394   119.854    -0.257 -0.091456
  36  C20   ca    S   35  34  33     1.393   120.630     0.563 -0.262595
  37  H12   ha    E   36  35  34     1.102   119.828  -179.299  0.170223
  38  H13   ha    E   35  34  33     1.101   119.985  -179.627  0.139410
  39  H15   ha    E   34  33  32     1.101   119.995   179.503  0.149962
  40  H14   ha    E   33  32  31     1.101   119.456   179.325  0.139410
  41  H11   ha    E   32  31  29     1.101   120.600     0.604  0.170223
  42  C2    cd    M   28  26  24     1.401   126.088  -142.738  0.167696
  43  C6    c3    B   42  28  26     1.479   131.928    -2.991 -0.293700
  44  H1    h1    E   43  42  28     1.133   108.055   -69.241  0.138615
  45  H2    h1    E   43  42  28     1.132   109.605    47.936  0.138615
  46  C4    cd    M   42  28  26     1.453   105.125   177.852  0.318000
  47  C7    c3    M   46  42  28     1.478   121.020  -178.398 -0.161125
  48  H4    hc    E   47  46  42     1.123   109.342   103.309  0.102135
  49  H5    hc    E   47  46  42     1.122   111.270  -137.770  0.102135
  50  C9    c3    M   47  46  42     1.539   109.784   -17.654  0.001065
  51  H6    h1    E   50  47  46     1.129   107.843   166.162  0.055269
  52  H7    h1    E   50  47  46     1.130   107.891   -76.994  0.055269
  53  N3    n3    M   50  47  46     1.441   114.968    44.525 -0.193397
  54  S1    sy    M   53  50  47     1.634   122.681   119.418  1.083060
  55  O1    o     E   54  53  50     1.410   109.812  -143.768 -0.557819
  56  O2    o     E   54  53  50     1.413   109.048   -14.922 -0.557819
  57  C5    cc    M   54  53  50     1.665   103.505   100.669 -0.124170
  58  C11   cd    M   57  54  53     1.404   120.204    99.467 -0.047194
  59  H22   ha    E   58  57  54     1.097   123.602    -0.742  0.157837
  60  C13   cd    M   58  57  54     1.414   112.230   178.307 -0.250696
  61  H24   ha    E   60  58  57     1.092   123.993  -179.985  0.179924
  62  C12   cc    M   60  58  57     1.392   110.731    -0.040 -0.128966
  63  H23   h4    E   62  60  58     1.090   124.214  -179.882  0.201283
  64  S2    ss    M   62  60  58     1.637   111.724    -0.271  0.045987


LOOP
```

```
  C14  C17
   C4   N2
  C20   C8
   N3   C6
   S2   C5

IMPROPER
  C24   O4  C25   O5
  C24  C17  C15  C10
  C15  C14  C17   H9
  C15  C16  C10  H16
  C10  C18  C16   H8
  C16  C14  C18  H10
  C17  C18  C14   N4
   C3  C14   N4   H3
   C1   N4   C3   O3
   C3   C2   C1   N1
   C8   C1   N1   N2
  C19  C20   C8   N1
   C8  C22  C19  H11
  C19  C23  C22  H14
  C22  C21  C23  H15
  C23  C20  C21  H13
   C8  C21  C20  H12
   C6   C1   C2   C4
   C7   C2   C4   N2
  C11   S2   C5   S1
   C5  C13  C11  H22
  C12  C11  C13  H24
  C13  H23  C12   S2

DONE
STOP
```

l48.in

```
    0    0    2

This is a remark line
molecule.res
L48   INT  0
CORRECT     OMIT DU   BEG
  0.0000
   1  DUMM  DU    M    0  -1  -2     0.000      .0        .0      .00000
   2  DUMM  DU    M    1   0  -1     1.449      .0        .0      .00000
   3  DUMM  DU    M    2   1   0     1.523   111.21       .0      .00000
   4  C26   c3    M    3   2   1     1.540   111.208  -180.000 -0.376000
   5  H22   hc    E    4   3   2     1.117    71.313    51.907  0.095143
   6  H23   hc    E    4   3   2     1.115    76.729   167.821  0.095143
   7  H24   hc    E    4   3   2     1.116    62.163   -71.565  0.095143
   8  C25   c3    M    4   3   2     1.511   172.217   -52.346  0.520621
   9  H20   h1    E    8   4   3     1.122   111.375   161.130 -0.064602
  10  H21   h1    E    8   4   3     1.122   111.428   -76.462 -0.064602
  11  O5    os    M    8   4   3     1.434   106.277    42.628 -0.497083
  12  C24   c     M   11   8   4     1.376   117.468  -174.589  0.805782
  13  O4    o     E   12  11   8     1.240   112.395   175.681 -0.585076
  14  C10   ca    M   12  11   8     1.476   120.654    -4.272 -0.204205
  15  C16   ca    B   14  12  11     1.397   119.811   110.758 -0.062188
  16  C18   ca    S   15  14  12     1.392   120.761   178.228 -0.369342
  17  H10   ha    E   16  15  14     1.102   118.925   179.907  0.200116
  18  H8    ha    E   15  14  12     1.101   119.853    -1.339  0.168331
  19  C15   ca    M   14  12  11     1.399   120.615   -72.177 -0.062188
  20  H19   ha    E   19  14  12     1.101   119.914     1.483  0.168331
  21  C17   ca    M   19  14  12     1.388   120.454  -177.862 -0.369342
  22  H9    ha    E   21  19  14     1.101   118.967   179.619  0.200116
  23  C14   ca    M   21  19  14     1.419   120.486    -0.017  0.548375
  24  N4    n     M   23  21  19     1.403   118.096  -179.274 -0.882091
  25  H3    hn    E   24  23  21     0.998   115.683    -0.357  0.399391
  26  C3    c     M   24  23  21     1.388   127.244  -175.857  1.120112
  27  O3    o     E   26  24  23     1.241   124.346    -2.246 -0.601397
  28  C1    cc    M   26  24  23     1.487   114.221   176.817 -0.637579
  29  N1    na    B   28  26  24     1.413   125.269    80.361  0.359130
  30  N2    nc    E   29  28  26     1.346   111.684  -179.666 -0.545711
  31  C8    ca    S   29  28  26     1.428   125.438     6.239  0.166374
  32  C19   ca    B   31  29  28     1.409   120.269    38.098 -0.232554
  33  C22   ca    B   32  31  29     1.393   119.565  -179.422 -0.075796
  34  C23   ca    B   33  32  31     1.394   120.609    -0.798 -0.183471
  35  C21   ca    B   34  33  32     1.394   119.823    -0.032 -0.075796
  36  C20   ca    S   35  34  33     1.392   120.655     0.456 -0.232554
  37  H12   ha    E   36  35  34     1.102   119.756  -179.455  0.159510
  38  H13   ha    E   35  34  33     1.101   120.019  -179.566  0.135250
  39  H15   ha    E   34  33  32     1.101   120.093  -179.675  0.143270
  40  H14   ha    E   33  32  31     1.101   119.277   179.711  0.135250
  41  H11   ha    E   32  31  29     1.103   120.739     0.631  0.159510
  42  C2    cd    M   28  26  24     1.398   128.307   -98.348  0.278308
  43  C6    c3    B   42  28  26     1.480   132.493    -1.508 -0.384470
  44  H1    h1    E   43  42  28     1.132   108.295   -70.794  0.150073
  45  H2    h1    E   43  42  28     1.132   109.764    47.026  0.150073
  46  C4    cd    M   42  28  26     1.454   104.910   179.192  0.251379
  47  C7    c3    M   46  42  28     1.478   121.248  -178.584 -0.145848
  48  H4    hc    E   47  46  42     1.123   109.303   104.436  0.102174
  49  H5    hc    E   47  46  42     1.122   111.277  -136.641  0.102174
  50  C9    c3    M   47  46  42     1.538   109.894   -16.508 -0.006934
  51  H6    h1    E   50  47  46     1.128   108.006   165.462  0.061665
  52  H7    h1    E   50  47  46     1.129   108.039   -77.533  0.061665
  53  N3    n3    M   50  47  46     1.442   114.823    43.843 -0.181915
  54  S1    sy    M   53  50  47     1.636   122.879   116.411  1.071330
  55  O1    o     E   54  53  50     1.411   109.553  -140.734 -0.554926
  56  O2    o     E   54  53  50     1.411   108.942   -12.162 -0.554926
  57  C5    cc    M   54  53  50     1.662   103.478   103.777 -0.125736
  58  C11   cd    M   57  54  53     1.405   120.193   104.833 -0.021490
  59  H17   ha    E   58  57  54     1.096   123.592    -0.815  0.148471
  60  C13   cd    M   58  57  54     1.414   112.253   178.225 -0.266294
```

```
  61  H18   ha    E   60  58  57     1.092   124.022  -179.877  0.187790
  62  C12   cc    M   60  58  57     1.392   110.715    -0.028 -0.134682
  63  H16   h4    E   62  60  58     1.091   124.147  -179.957  0.207899
  64  S2    ss    M   62  60  58     1.636   111.753    -0.203  0.050898


LOOP
  C14  C18
   C4   N2
  C20   C8
   N3   C6
   S2   C5

IMPROPER
  C10   O4  C24   O5
  C24  C16  C10  C15
  C10  C18  C16   H8
  C16  C14  C18  H10
  C10  C17  C15  H19
  C15  C14  C17   H9
  C18  C17  C14   N4
   C3  C14   N4   H3
   C1   N4   C3   O3
   C3   C2   C1   N1
   C8   C1   N1   N2
  C19  C20   C8   N1
   C8  C22  C19  H11
  C19  C23  C22  H14
  C22  C21  C23  H15
  C23  C20  C21  H13
   C8  C21  C20  H12
   C6   C1   C2   C4
   C7   C2   C4   N2
  C11   S2   C5   S1
   C5  C13  C11  H17
  C12  C11  C13  H18
  C13  H16  C12   S2

DONE
STOP
```

l49.in

```
    0    0    2

This is a remark line
molecule.res
L49   INT  0
CORRECT     OMIT DU   BEG
  0.0000
   1  DUMM  DU    M    0  -1  -2     0.000      .0        .0      .00000
   2  DUMM  DU    M    1   0  -1     1.449      .0        .0      .00000
   3  DUMM  DU    M    2   1   0     1.523   111.21       .0      .00000
   4  O4    o     M    3   2   1     1.540   111.208  -180.000 -0.570804
   5  C24   c     M    4   3   2     1.239    79.726  -123.390  0.713623
   6  C25   c3    3    5   4   3     1.497   120.959   139.070 -0.447592
   7  H20   hc    E    6   5   4     1.117   109.917   117.668  0.113669
   8  H21   hc    E    6   5   4     1.118   110.291    -2.240  0.113669
   9  H22   hc    E    6   5   4     1.118   110.005  -122.325  0.113669
  10  C10   ca    M    5   4   3     1.476   121.579   -40.810 -0.193688
  11  C16   ca    B   10   5   4     1.401   119.183     3.584 -0.043782
  12  C18   ca    S   11  10   5     1.390   121.354   179.982 -0.350467
  13  H10   ha    E   12  11  10     1.103   119.039  -179.728  0.188131
  14  H8    ha    E   11  10   5     1.104   118.923    -0.082  0.148767
  15  C15   ca    M   10   5   4     1.400   122.118  -176.384 -0.043782
  16  H19   ha    E   15  10   5     1.101   120.302    -0.042  0.148767
  17  C17   ca    M   15  10   5     1.388   120.814   179.957 -0.350467
  18  H9    ha    E   17  15  10     1.101   118.957   179.943  0.188131
  19  C14   ca    M   17  15  10     1.419   120.567     0.038  0.458383
  20  N4    n     M   19  17  15     1.403   118.065  -179.541 -0.738022
  21  H3    hn    E   20  19  17     0.998   115.593     1.199  0.334513
  22  C3    c     M   20  19  17     1.387   127.186  -175.070  1.063920
  23  O3    o     E   22  20  19     1.242   124.281    -1.111 -0.594231
  24  C1    cc    M   22  20  19     1.485   114.837   177.603 -0.582248
  25  N1    na    B   24  22  20     1.416   125.494    59.050  0.332355
  26  N2    nc    E   25  24  22     1.347   111.643  -179.330 -0.559353
  27  C8    ca    S   25  24  22     1.428   125.019    10.634  0.205882
  28  C19   ca    B   27  25  24     1.410   120.270    33.748 -0.233447
  29  C22   ca    B   28  27  25     1.393   119.568  -179.205 -0.084361
  30  C23   ca    B   29  28  27     1.394   120.606    -0.792 -0.185457
  31  C21   ca    B   30  29  28     1.395   119.817    -0.041 -0.084361
  32  C20   ca    S   31  30  29     1.392   120.677     0.415 -0.233447
  33  H12   ha    E   32  31  30     1.102   119.793  -179.356  0.153525
  34  H13   ha    E   31  30  29     1.101   120.026  -179.501  0.140312
  35  H15   ha    E   30  29  28     1.100   120.065  -179.826  0.145949
  36  H14   ha    E   29  28  27     1.100   119.282   179.567  0.140312
  37  H11   ha    E   28  27  25     1.102   120.707     0.980  0.153525
  38  C2    cd    M   24  22  20     1.397   128.123  -118.564  0.224417
  39  C6    c3    B   38  24  22     1.479   132.387    -2.106 -0.373951
  40  H1    h1    E   39  38  24     1.133   108.196   -70.268  0.145348
  41  H2    h1    E   39  38  24     1.132   109.764    47.364  0.145348
  42  C4    cd    M   38  24  22     1.455   104.946   178.506  0.284082
  43  C7    c3    M   42  38  24     1.478   121.178  -178.411 -0.121014
  44  H4    hc    E   43  42  38     1.123   109.331   103.946  0.094333
  45  H5    hc    E   43  42  38     1.122   111.220  -137.062  0.094333
  46  C9    c3    M   43  42  38     1.539   109.775   -17.022 -0.041504
  47  H6    h1    E   46  43  42     1.129   107.870   165.810  0.067393
  48  H7    h1    E   46  43  42     1.129   108.007   -77.329  0.067393
  49  N3    n3    M   46  43  42     1.441   114.922    44.233 -0.148159
  50  S1    sy    M   49  46  43     1.635   122.817   117.846  1.061011
  51  O1    o     E   50  49  46     1.411   109.647  -142.344 -0.553347
  52  O2    o     E   50  49  46     1.413   108.950   -13.660 -0.553347
  53  C5    cc    M   50  49  46     1.662   103.497   102.080 -0.126758
```

```
  54  C11   cd    M   53  50  49     1.405   120.209   101.673 -0.021164
  55  H17   ha    E   54  53  50     1.096   123.565    -0.679  0.151493
  56  C13   cd    M   54  53  50     1.412   112.282   178.282 -0.269421
  57  H18   ha    E   56  54  53     1.092   124.086  -179.924  0.188306
  58  C12   cc    M   56  54  53     1.392   110.760    -0.008 -0.133856
  59  H16   h4    E   58  56  54     1.091   124.255  -179.886  0.207293
  60  S2    ss    M   58  56  54     1.637   111.738    -0.245  0.050180


LOOP
  C14  C18
   C4   N2
  C20   C8
   N3   C6
   S2   C5

IMPROPER
  C25  C10  C24   O4
  C24  C16  C10  C15
  C18  C10  C16   H8
  C16  C14  C18  H10
  C17  C10  C15  H19
  C15  C14  C17   H9
  C18  C17  C14   N4
   C3  C14   N4   H3
   C1   N4   C3   O3
   C3   C2   C1   N1
   C8   C1   N1   N2
  C19  C20   C8   N1
   C8  C22  C19  H11
  C19  C23  C22  H14
  C22  C21  C23  H15
  C23  C20  C21  H13
   C8  C21  C20  H12
   C6   C1   C2   C4
   C7   C2   C4   N2
  C11   S2   C5   S1
   C5  C13  C11  H17
  C12  C11  C13  H18
  C13  H16  C12   S2

DONE
STOP
```

l73.in

```
    0    0    2

This is a remark line
molecule.res
L73   INT  0
CORRECT     OMIT DU   BEG
  0.0000
   1  DUMM  DU    M    0  -1  -2     0.000      .0        .0      .00000
   2  DUMM  DU    M    1   0  -1     1.449      .0        .0      .00000
   3  DUMM  DU    M    2   1   0     1.523   111.21       .0      .00000
   4  O4    oh    M    3   2   1     1.540   111.208  -180.000 -0.538794
   5  H21   ho    E    4   3   2     0.969   171.961   -73.706  0.362323
   6  C11   ca    M    4   3   2     1.375    75.896    46.428  0.406067
   7  C10   ca    B    6   4   3     1.407   116.479     6.947 -0.323593
   8  C9    ca    S    7   6   4     1.389   119.018   179.880 -0.157088
   9  H7    ha    E    8   7   6     1.103   120.130   179.814  0.157218
  10  H8    ha    E    7   6   4     1.099   119.565    -0.089  0.189632
  11  C8    ca    M    6   4   3     1.402   122.679  -173.084 -0.323593
  12  H6    ha    E   11   6   4     1.099   120.321     0.070  0.189632
  13  C7    ca    M   11   6   4     1.393   119.207   179.994 -0.157088
  14  H5    ha    E   13  11   6     1.101   119.422  -179.790  0.157218
  15  C6    ca    M   13  11   6     1.396   120.493     0.285  0.122532
  16  C5    c3    M   15  13  11     1.501   120.619   178.209 -0.233725
  17  H3    h1    E   16  15  13     1.131   108.502  -133.313  0.129754
  18  H4    h1    E   16  15  13     1.129   109.568   -14.965  0.129754
  19  N1    n     M   16  15  13     1.438   115.444   106.946 -0.305810
  20  C15   c     S   19  16  15     1.416   124.441    84.085  0.661947
  21  O1    o     E   20  19  16     1.236   123.602     8.646 -0.525598
  22  C4    ca    M   19  16  15     1.407   124.943   -82.734  0.320172
  23  C3    ca    M   22  19  16     1.395   129.506    -9.441 -0.335342
  24  H2    ha    E   23  22  19     1.098   121.386     2.286  0.163858
  25  C2    ca    M   23  22  19     1.399   118.360  -178.045  0.062726
  26  H1    ha    E   25  23  22     1.102   118.466   179.932  0.115565
  27  C1    ca    M   25  23  22     1.394   121.386    -0.170 -0.197570
  28  Br1   br    E   27  25  23     1.871   120.017   179.972 -0.069569
  29  C12   ca    M   27  25  23     1.407   120.582    -0.047  0.015150
  30  H9    ha    E   29  27  25     1.102   120.847  -179.862  0.145622
  31  C13   ca    M   29  27  25     1.378   118.795     0.094 -0.278082
  32  C14   c3    M   31  29  27     1.511   130.088  -179.961  0.145997
  33  C19   c3    3   32  31  29     1.523   111.298    61.500 -0.231491
  34  C18   c3    B   33  32  31     1.531   111.944  -174.451  0.107357
  35  H14   h1    E   34  33  32     1.129   108.361   172.038  0.022169
  36  H15   h1    E   34  33  32     1.130   108.067   -70.239  0.022169
  37  H16   hc    E   33  32  31     1.120   110.136    64.957  0.105862
  38  H17   hc    E   33  32  31     1.122   108.294   -52.889  0.105862
  39  C16   c3    M   32  31  29     1.523   111.396   -63.113 -0.231491
  40  H10   hc    E   39  32  31     1.122   108.263    53.107  0.105862
  41  H11   hc    E   39  32  31     1.120   110.161   -64.728  0.105862
  42  C17   c3    M   39  32  31     1.531   111.878   174.616  0.107357
  43  H12   h1    E   42  39  32     1.129   108.411  -172.796  0.022169
  44  H13   h1    E   42  39  32     1.131   107.933    69.532  0.022169
  45  N2    n3    M   42  39  32     1.440   113.465   -51.404 -0.276794
  46  S1    sy    M   45  42  39     1.629   123.313  -127.518  1.077734
  47  O2    o     E   46  45  42     1.413   109.596   145.767 -0.563739
  48  O3    o     E   46  45  42     1.414   109.081    17.265 -0.563739
  49  C20   cc    M   46  45  42     1.665   103.875   -98.660 -0.135109
  50  C21   cd    M   49  46  45     1.405   120.128  -104.225 -0.038208
```

```
  51  H18   ha    E   50  49  46     1.097   123.534     0.551  0.160403
  52  C22   cd    M   50  49  46     1.413   112.229  -178.418 -0.268884
  53  H19   ha    E   52  50  49     1.092   124.017   179.826  0.182347
  54  C23   cc    M   52  50  49     1.391   110.711    -0.056 -0.117537
  55  H20   h4    E   54  52  50     1.091   124.202   179.983  0.198658
  56  S2    ss    M   54  52  50     1.636   111.740     0.222  0.051698


LOOP
   C6   C9
  C14  C15
  C13   C4
   N2  C18
   S2  C20

IMPROPER
   C8  C10  C11   O4
  C11   C9  C10   H8
   C6  C10   C9   H7
   C7  C11   C8   H6
   C8   C6   C7   H5
   C5   C7   C6   C9
  C15   C5   N1   C4
  C14   N1  C15   O1
   C3  C13   C4   N1
   C4   C2   C3   H2
   C3   C1   C2   H1
  Br1   C2   C1  C12
   C1  C13  C12   H9
  C14   C4  C13  C12
  C21   S2  C20   S1
  C20  C22  C21  H18
  C23  C21  C22  H19
  C22  H20  C23   S2

DONE
STOP
```

l74.in

```
    0    0    2

This is a remark line
molecule.res
L74   INT  0
CORRECT     OMIT DU   BEG
  0.0000
   1  DUMM  DU    M    0  -1  -2     0.000      .0        .0      .00000
   2  DUMM  DU    M    1   0  -1     1.449      .0        .0      .00000
   3  DUMM  DU    M    2   1   0     1.523   111.21       .0      .00000
   4  O4    o     M    3   2   1     1.540   111.208  -180.000 -0.800220
   5  C12   c     M    4   3   2     1.263    54.802   139.007  0.823903
   6  O5    o     E    5   4   3     1.262   124.542     4.742 -0.800220
   7  C11   ca    M    5   4   3     1.524   117.708  -175.153 -0.033293
   8  C10   ca    B    7   5   4     1.399   120.448     1.321 -0.119670
   9  C9    ca    S    8   7   5     1.393   120.514   179.929 -0.256828
  10  H7    ha    E    9   8   7     1.100   120.514  -179.614  0.138949
  11  H8    ha    E    8   7   5     1.102   118.245     0.037  0.133240
  12  C8    ca    M    7   5   4     1.398   120.369  -178.744 -0.119670
  13  H6    ha    E   12   7   5     1.102   118.216    -0.118  0.133240
  14  C7    ca    M   12   7   5     1.393   120.555   179.895 -0.256828
  15  H5    ha    E   14  12   7     1.100   120.054  -179.983  0.138949
  16  C6    ca    M   14  12   7     1.399   120.054     0.329  0.166645
  17  C5    c3    M   16  14  12     1.497   120.398   178.104 -0.176521
  18  H3    h1    E   17  16  14     1.129   109.773   -13.333  0.105088
  19  H4    h1    E   17  16  14     1.130   108.996  -132.271  0.105088
  20  N1    n     M   17  16  14     1.444   115.827   107.968 -0.356106
  21  C16   c     S   20  17  16     1.415   123.919    88.571  0.695632
  22  O1    o     E   21  20  17     1.236   124.358    16.025 -0.565458
  23  C4    ca    M   20  17  16     1.407   124.382   -71.339  0.339931
  24  C3    ca    M   23  20  17     1.396   129.264   -14.973 -0.324029
  25  H2    ha    E   24  23  20     1.100   121.075     4.621  0.177214
  26  C2    ca    M   24  23  20     1.398   118.401  -176.754  0.042144
  27  H1    ha    E   26  24  23     1.103   118.478  -179.693  0.120515
  28  C1    ca    M   26  24  23     1.396   121.375    -0.149 -0.179751
  29  Br1   br    E   28  26  24     1.874   119.974   179.486 -0.094743
  30  C13   ca    M   28  26  24     1.405   120.642    -0.816 -0.006256
  31  H9    ha    E   30  28  26     1.100   120.917  -178.863  0.141171
  32  C14   ca    M   30  28  26     1.379   118.680     0.830 -0.247294
  33  C15   c3    M   32  30  28     1.510   130.073  -179.260  0.152339
  34  C20   c3    3   33  32  30     1.524   111.029    63.036 -0.247016
  35  C19   c3    B   34  33  32     1.530   111.773  -174.846  0.104292
  36  H14   h1    E   35  34  33     1.130   108.197   -69.630  0.035727
  37  H15   h1    E   35  34  33     1.129   108.589   172.363  0.035727
  38  H16   hc    E   34  33  32     1.121   110.067    64.762  0.096355
  39  H17   hc    E   34  33  32     1.122   108.382   -53.147  0.096355
  40  C17   c3    M   33  32  30     1.521   111.706   -61.716 -0.247016
  41  H10   hc    E   40  33  32     1.121   108.399    52.921  0.096355
  42  H11   hc    E   40  33  32     1.120   110.115   -64.934  0.096355
  43  C18   c3    M   40  33  32     1.531   111.858   174.744  0.104292
  44  H12   h1    E   43  40  33     1.128   108.977  -173.416  0.035727
  45  H13   h1    E   43  40  33     1.131   107.971    68.643  0.035727
  46  N2    n3    M   43  40  33     1.443   113.246   -51.766 -0.231227
  47  S1    sy    M   46  43  40     1.613   123.746  -124.237  1.009024
  48  O2    o     E   47  46  43     1.416   110.522   128.988 -0.577032
  49  O3    o     E   47  46  43     1.410   111.456     0.746 -0.577032
  50  C21   ca    M   47  46  43     1.721   103.566  -117.625  0.102645
  51  C22   ca    M   50  47  46     1.404   115.813  -113.531 -0.154434
  52  H18   ha    E   51  50  47     1.107   119.113     1.136  0.172414
  53  C23   ca    M   51  50  47     1.392   121.790  -178.742 -0.152722
  54  H19   ha    E   53  51  50     1.100   119.995  -179.836  0.146674
  55  C24   ca    M   53  51  50     1.394   119.626    -0.165 -0.100248
  56  H20   ha    E   55  53  51     1.101   120.358  -179.716  0.139939
```

```
  57  C26   ca    M   55  53  51     1.393   119.689     0.055 -0.063743
  58  H21   ha    E   57  55  53     1.102   119.357  -179.758  0.125575
  59  C25   ca    M   57  55  53     1.398   120.283    -0.077 -0.102483
  60  Br2   br    M   59  57  55     1.877   116.853  -179.550 -0.057391


LOOP
   C6   C9
  C15  C16
  C14   C4
   N2  C19
  C25  C21

IMPROPER
  C11   O4  C12   O5
  C12   C8  C11  C10
  C11   C9  C10   H8
   C6  C10   C9   H7
   C7  C11   C8   H6
   C8   C6   C7   H5
   C5   C7   C6   C9
  C16   C5   N1   C4
  C15   N1  C16   O1
   C3  C14   C4   N1
   C4   C2   C3   H2
   C3   C1   C2   H1
  Br1   C2   C1  C13
   C1  C14  C13   H9
  C15   C4  C14  C13
  C22  C25  C21   S1
  C21  C23  C22  H18
  C22  C24  C23  H19
  C23  C26  C24  H20
  C24  C25  C26  H21
  Br2  C21  C25  C26

DONE
STOP
```

l75.in

```
    0    0    2

This is a remark line
molecule.res
L75   INT  0
CORRECT     OMIT DU   BEG
  0.0000
   1  DUMM  DU    M    0  -1  -2     0.000      .0        .0      .00000
   2  DUMM  DU    M    1   0  -1     1.449      .0        .0      .00000
   3  DUMM  DU    M    2   1   0     1.523   111.21       .0      .00000
   4  N2    nb    M    3   2   1     1.540   111.208  -180.000 -0.673896
   5  C10   ca    B    4   3   2     1.348    18.268    50.281  0.458087
   6  C9    ca    S    5   4   3     1.406   123.571  -179.254 -0.601506
   7  H7    ha    E    6   5   4     1.100   121.041  -179.902  0.203687
   8  H8    h4    E    5   4   3     1.105   115.751     0.836  0.048164
   9  C8    ca    M    4   3   2     1.346   135.512    49.214  0.458087
  10  H6    h4    E    9   4   3     1.105   115.792  -179.581  0.048164
  11  C7    ca    M    9   4   3     1.408   123.596     0.324 -0.601506
  12  H5    ha    E   11   9   4     1.097   120.360  -179.755  0.203687
  13  C6    ca    M   11   9   4     1.398   118.405     0.213  0.576115
  14  C5    c3    M   13  11   9     1.503   120.947   178.090 -0.368068
  15  H3    h1    E   14  13  11     1.131   108.148  -129.368  0.157884
  16  H4    h1    E   14  13  11     1.128   109.476   -11.204  0.157884
  17  N1    n     M   14  13  11     1.435   115.283   110.878 -0.245423
  18  C14   c     S   17  14  13     1.417   124.672    85.087  0.653578
  19  O1    o     E   18  17  14     1.235   123.510     6.566 -0.525006
  20  C4    ca    M   17  14  13     1.408   124.984   -84.199  0.254571
  21  C3    ca    M   20  17  14     1.393   129.528    -7.341 -0.302210
  22  H2    ha    E   21  20  17     1.099   121.442     1.976  0.167313
  23  C2    ca    M   21  20  17     1.399   118.356  -178.339  0.040656
  24  H1    ha    E   23  21  20     1.103   118.475   179.973  0.119812
  25  C1    ca    M   23  21  20     1.394   121.358    -0.150 -0.176231
  26  Br1   br    E   25  23  21     1.871   120.004   179.980 -0.066201
  27  C11   ca    M   25  23  21     1.407   120.636    -0.087 -0.006664
  28  H9    ha    E   27  25  23     1.102   120.905  -179.794  0.148994
  29  C12   ca    M   27  25  23     1.379   118.742     0.090 -0.238069
  30  C13   c3    M   29  27  25     1.510   130.037  -179.919  0.120357
  31  C18   c3    3   30  29  27     1.522   111.353    61.505 -0.222474
  32  C17   c3    B   31  30  29     1.530   112.001  -174.386  0.115176
  33  H14   h1    E   32  31  30     1.129   108.470   172.071  0.018653
  34  H15   h1    E   32  31  30     1.131   108.092   -70.278  0.018653
  35  H16   hc    E   31  30  29     1.121   110.159    64.930  0.105056
  36  H17   hc    E   31  30  29     1.122   108.256   -52.854  0.105056
  37  C15   c3    M   30  29  27     1.523   111.397   -63.153 -0.222474
  38  H10   hc    E   37  30  29     1.122   108.246    53.049  0.105056
  39  H11   hc    E   37  30  29     1.121   110.129   -64.689  0.105056
  40  C16   c3    M   37  30  29     1.531   111.907   174.617  0.115176
  41  H12   h1    E   40  37  30     1.129   108.426  -172.782  0.018653
  42  H13   h1    E   40  37  30     1.131   107.917    69.544  0.018653
  43  N3    n3    M   40  37  30     1.440   113.444   -51.381 -0.281461
  44  S1    sy    M   43  40  37     1.630   123.310  -127.247  1.076744
  45  O2    o     E   44  43  40     1.412   109.509   145.622 -0.562522
  46  O3    o     E   44  43  40     1.413   109.042    17.181 -0.562522
  47  C19   cc    M   44  43  40     1.664   103.837   -98.801 -0.127492
  48  C20   cd    M   47  44  43     1.404   120.148  -103.994 -0.042109
  49  H18   ha    E   48  47  44     1.097   123.551     0.673  0.161004
  50  C21   cd    M   48  47  44     1.413   112.248  -178.370 -0.270361
  51  H19   ha    E   50  48  47     1.092   124.035   179.835  0.183524
  52  C22   cc    M   50  48  47     1.391   110.728     0.048 -0.117028
  53  H20   h4    E   52  50  48     1.091   124.249   179.881  0.199416
  54  S2    ss    M   52  50  48     1.636   111.736     0.215  0.050304
```

```
LOOP
   C6   C9
  C13  C14
  C12   C4
   N3  C17
   S2  C19

IMPROPER
   C9   H8  C10   N2
   C6  C10   C9   H7
   C7   H6   C8   N2
   C6   C8   C7   H5
   C5   C7   C6   C9
  C14   C5   N1   C4
  C13   N1  C14   O1
   C3  C12   C4   N1
   C4   C2   C3   H2
   C3   C1   C2   H1
  Br1   C2   C1  C11
   C1  C12  C11   H9
  C13   C4  C12  C11
  C20   S2  C19   S1
  C19  C21  C20  H18
  C22  C20  C21  H19
  C21  H20  C22   S2

DONE
STOP
```

l76.in

```
    0    0    2

This is a remark line
molecule.res
L76   INT  0
CORRECT     OMIT DU   BEG
  0.0000
   1  DUMM  DU    M    0  -1  -2     0.000      .0        .0      .00000
   2  DUMM  DU    M    1   0  -1     1.449      .0        .0      .00000
   3  DUMM  DU    M    2   1   0     1.523   111.21       .0      .00000
   4  O4    o     M    3   2   1     1.540   111.208  -180.000 -0.796966
   5  C12   c     M    4   3   2     1.263   103.927  -121.966  0.814982
   6  O5    o     E    5   4   3     1.263   124.561   179.439 -0.796966
   7  C11   ca    M    5   4   3     1.524   117.743    -0.588 -0.015052
   8  C10   ca    B    7   5   4     1.399   120.517    -0.574 -0.134099
   9  C9    ca    S    8   7   5     1.393   120.526   179.826 -0.233246
  10  H7    ha    E    9   8   7     1.099   120.194  -179.499  0.133167
  11  H8    ha    E    8   7   5     1.102   118.225    -0.015  0.135257
  12  C8    ca    M    7   5   4     1.399   120.324   179.295 -0.134099
  13  H6    ha    E   12   7   5     1.103   118.193    -0.075  0.135257
  14  C7    ca    M   12   7   5     1.393   120.545   179.952 -0.233246
  15  H5    ha    E   14  12   7     1.099   119.959   179.593  0.133167
  16  C6    ca    M   14  12   7     1.400   120.101     0.265  0.141479
  17  C5    c3    M   16  14  12     1.496   119.913   177.576 -0.138825
  18  H3    h1    E   17  16  14     1.130   109.427    -7.080  0.088247
  19  H4    h1    E   17  16  14     1.131   109.087  -125.420  0.088247
  20  N1    n     M   17  16  14     1.443   115.926   114.221 -0.305192
  21  C16   c     S   20  17  16     1.410   124.717   114.791  0.629829
  22  O1    o     E   21  20  17     1.237   124.499     6.366 -0.558054
  23  C4    ca    M   20  17  16     1.406   125.296   -57.093  0.302980
  24  C3    ca    M   23  20  17     1.397   129.418    -4.987 -0.258302
  25  H2    ha    E   24  23  20     1.101   120.752     3.844  0.160243
  26  C2    ca    M   24  23  20     1.397   118.327  -178.078  0.012978
  27  H1    ha    E   26  24  23     1.103   118.395  -179.552  0.126839
  28  C1    ca    M   26  24  23     1.396   121.393     0.014 -0.154671
  29  Br1   br    E   28  26  24     1.874   119.979   179.364 -0.100904
  30  C13   ca    M   28  26  24     1.404   120.673    -0.936 -0.011921
  31  H9    ha    E   30  28  26     1.101   120.942  -178.960  0.137066
  32  C14   ca    M   30  28  26     1.378   118.746     0.838 -0.310461
  33  C15   c3    M   32  30  28     1.509   130.233  -179.277  0.215563
  34  C20   c3    3   33  32  30     1.522   111.241    62.576 -0.176118
  35  C19   c3    B   34  33  32     1.529   111.839  -175.244  0.028964
  36  H14   h1    E   35  34  33     1.132   108.037   -69.763  0.045663
  37  H15   h1    E   35  34  33     1.129   108.572   172.378  0.045663
  38  H16   hc    E   34  33  32     1.122   110.039    64.351  0.084883
  39  H17   hc    E   34  33  32     1.121   108.426   -53.514  0.084883
  40  C17   c3    M   33  32  30     1.523   111.603   -61.913 -0.176118
  41  H10   hc    E   40  33  32     1.121   108.352    53.491  0.084883
  42  H11   hc    E   40  33  32     1.120   110.052   -64.420  0.084883
  43  C18   c3    M   40  33  32     1.529   111.802   175.201  0.028964
  44  H12   h1    E   43  40  33     1.129   108.585  -172.798  0.045663
  45  H13   h1    E   43  40  33     1.131   108.009    69.365  0.045663
  46  N2    n3    M   43  40  33     1.442   113.534   -51.508 -0.254868
  47  S1    sy    M   46  43  40     1.619   123.063  -130.809  1.040139
  48  O2    o     E   47  46  43     1.414   110.314   151.742 -0.572710
  49  O3    o     E   47  46  43     1.416   110.088    22.139 -0.572710
  50  C21   ca    M   47  46  43     1.695   103.585   -93.111  0.012144
  51  C22   ca    M   50  47  46     1.399   120.630   -88.514 -0.151643
  52  H18   ha    E   51  50  47     1.105   119.189     0.097  0.149549
  53  C23   ca    M   51  50  47     1.393   120.902  -179.208 -0.113333
  54  H19   ha    E   53  51  50     1.100   119.995  -179.818  0.132013
  55  C24   ca    M   53  51  50     1.395   119.782     0.075 -0.120218
  56  H20   ha    E   55  53  51     1.101   119.966  -179.655  0.133879
  57  C26   ca    M   55  53  51     1.394   119.996     0.307 -0.113333
  58  H21   ha    E   57  55  53     1.100   120.173   179.706  0.132013
  59  C25   ca    M   57  55  53     1.393   119.861    -0.289 -0.151643
  60  H22   ha    E   59  57  55     1.105   119.976   179.206  0.149549


LOOP
   C6   C9
```

```
  C15  C16
  C14   C4
   N2  C19
  C25  C21

IMPROPER
  C11   O4  C12   O5
  C12   C8  C11  C10
  C11   C9  C10   H8
   C6  C10   C9   H7
   C7  C11   C8   H6
   C8   C6   C7   H5
   C5   C7   C6   C9
  C16   C5   N1   C4
  C15   N1  C16   O1
   C3  C14   C4   N1
   C4   C2   C3   H2
   C3   C1   C2   H1
  Br1   C2   C1  C13
   C1  C14  C13   H9
  C15   C4  C14  C13
  C22  C25  C21   S1
  C21  C23  C22  H18
  C22  C24  C23  H19
  C23  C26  C24  H20
  C24  C25  C26  H21
  C21  C26  C25  H22

DONE
STOP
```

l77.in

```
    0    0    2

This is a remark line
molecule.res
L77   INT  0
CORRECT     OMIT DU   BEG
  0.0000
   1  DUMM  DU    M    0  -1  -2     0.000      .0        .0      .00000
   2  DUMM  DU    M    1   0  -1     1.449      .0        .0      .00000
   3  DUMM  DU    M    2   1   0     1.523   111.21       .0      .00000
   4  O4    o     M    3   2   1     1.540   111.208  -180.000 -0.797520
   5  C12   c     M    4   3   2     1.263     2.443    31.328  0.816926
   6  O5    o     E    5   4   3     1.262   124.488   -14.977 -0.797520
   7  C11   ca    M    5   4   3     1.524   117.700   164.985 -0.024965
   8  C10   ca    B    7   5   4     1.400   120.392  -178.599 -0.124469
   9  C9    ca    S    8   7   5     1.393   120.476   179.967 -0.251724
  10  H7    ha    E    9   8   7     1.100   120.450  -179.610  0.137861
  11  H8    ha    E    8   7   5     1.102   118.240     0.006  0.133492
  12  C8    ca    M    7   5   4     1.399   120.415     1.318 -0.124469
  13  H6    ha    E   12   7   5     1.102   118.126    -0.138  0.133492
  14  C7    ca    M   12   7   5     1.393   120.555   179.865 -0.251724
  15  H5    ha    E   14  12   7     1.099   120.024   179.977  0.137861
  16  C6    ca    M   14  12   7     1.399   120.051     0.321  0.164397
  17  C5    c3    M   16  14  12     1.497   120.387   178.217 -0.180144
  18  H3    h1    E   17  16  14     1.128   109.724   -13.085  0.106423
  19  H4    h1    E   17  16  14     1.130   109.018  -132.040  0.106423
  20  N1    n     M   17  16  14     1.445   115.773   108.152 -0.348016
  21  C16   c     S   20  17  16     1.415   123.886    89.590  0.668942
  22  O1    o     E   21  20  17     1.236   124.417    15.986 -0.557351
  23  C4    ca    M   20  17  16     1.406   124.382   -70.393  0.323866
  24  C3    ca    M   23  20  17     1.397   129.310   -14.921 -0.308936
  25  H2    ha    E   24  23  20     1.099   120.970     4.606  0.174961
  26  C2    ca    M   24  23  20     1.398   118.449  -176.798  0.028290
  27  H1    ha    E   26  24  23     1.102   118.485  -179.701  0.122880
  28  C1    ca    M   26  24  23     1.396   121.313    -0.155 -0.167063
  29  Br1   br    E   28  26  24     1.874   119.938   179.479 -0.096327
  30  C13   ca    M   28  26  24     1.405   120.689    -0.829 -0.016034
  31  H9    ha    E   30  28  26     1.101   120.912  -178.925  0.138708
  32  C14   ca    M   30  28  26     1.378   118.734     0.854 -0.243407
  33  C15   c3    M   32  30  28     1.509   130.157  -179.255  0.201366
  34  C20   c3    3   33  32  30     1.523   111.134    62.950 -0.286543
  35  C19   c3    B   34  33  32     1.529   111.853  -174.995  0.151111
  36  H14   h1    E   35  34  33     1.131   108.174   -69.584  0.031280
  37  H15   h1    E   35  34  33     1.128   108.526   172.418  0.031280
  38  H16   hc    E   34  33  32     1.121   110.013    64.585  0.106261
  39  H17   hc    E   34  33  32     1.121   108.384   -53.212  0.106261
  40  C17   c3    M   33  32  30     1.522   111.706   -61.759 -0.286543
  41  H10   hc    E   40  33  32     1.122   108.384    53.023  0.106261
  42  H11   hc    E   40  33  32     1.120   110.058   -64.822  0.106261
  43  C18   c3    M   40  33  32     1.530   111.883   174.886  0.151111
  44  H12   h1    E   43  40  33     1.128   108.893  -173.179  0.031280
  45  H13   h1    E   43  40  33     1.131   108.084    68.817  0.031280
  46  N2    n3    M   43  40  33     1.444   113.364   -51.651 -0.322391
  47  S1    sy    M   46  43  40     1.611   123.731  -126.048  1.108242
  48  O2    o     E   47  46  43     1.416   110.644   137.005 -0.586679
  49  O3    o     E   47  46  43     1.410   111.299     8.005 -0.586679
  50  C21   ca    M   47  46  43     1.722   103.634  -109.584 -0.063023
  51  C22   ca    M   50  47  46     1.402   116.628  -113.742 -0.126358
  52  H18   ha    E   51  50  47     1.107   119.059     1.256  0.182796
  53  C23   ca    M   51  50  47     1.391   121.562  -178.581 -0.188720
  54  H19   ha    E   53  51  50     1.100   120.037  -179.793  0.161828
  55  C24   ca    M   53  51  50     1.392   119.969    -0.007 -0.061958
  56  H20   ha    E   55  53  51     1.101   120.343  -179.782  0.139817
  57  C26   ca    M   55  53  51     1.399   119.643    -0.007  0.043179
  58  Cl1   cl    E   57  55  53     1.698   118.689   179.944 -0.096525
  59  C25   ca    M   57  55  53     1.405   120.101    -0.238  0.083447
  60  Cl2   cl    M   59  57  55     1.694   119.289  -179.350 -0.072494


LOOP
```

```
   C6   C9
  C15  C16
  C14   C4
   N2  C19
  C25  C21

IMPROPER
  C11   O4  C12   O5
  C12   C8  C11  C10
  C11   C9  C10   H8
   C6  C10   C9   H7
   C7  C11   C8   H6
   C8   C6   C7   H5
   C5   C7   C6   C9
  C16   C5   N1   C4
  C15   N1  C16   O1
   C3  C14   C4   N1
   C4   C2   C3   H2
   C3   C1   C2   H1
  Br1   C2   C1  C13
   C1  C14  C13   H9
  C15   C4  C14  C13
  C22  C25  C21   S1
  C21  C23  C22  H18
  C22  C24  C23  H19
  C23  C26  C24  H20
  C24  C25  C26  Cl1
  C21  C26  C25  Cl2

DONE
STOP
```

l78.in

```
    0    0    2

This is a remark line
molecule.res
L78   INT  0
CORRECT     OMIT DU   BEG
  0.0000
   1  DUMM  DU    M    0  -1  -2     0.000      .0        .0      .00000
   2  DUMM  DU    M    1   0  -1     1.449      .0        .0      .00000
   3  DUMM  DU    M    2   1   0     1.523   111.21       .0      .00000
   4  O4    o     M    3   2   1     1.540   111.208  -180.000 -0.796464
   5  C12   c     M    4   3   2     1.262    69.794   126.522  0.814151
   6  O5    o     E    5   4   3     1.263   124.534    -0.347 -0.796464
   7  C11   ca    M    5   4   3     1.524   117.772   179.665 -0.017802
   8  C10   ca    B    7   5   4     1.398   120.484    -0.407 -0.130609
   9  C9    ca    S    8   7   5     1.393   120.552   179.812 -0.239223
  10  H7    ha    E    9   8   7     1.099   120.279  -179.483  0.134780
  11  H8    ha    E    8   7   5     1.103   118.246    -0.040  0.134257
  12  C8    ca    M    7   5   4     1.399   120.328   179.437 -0.130609
  13  H6    ha    E   12   7   5     1.102   118.199    -0.012  0.134257
  14  C7    ca    M   12   7   5     1.393   120.509   179.954 -0.239223
  15  H5    ha    E   14  12   7     1.099   119.972   179.681  0.134780
  16  C6    ca    M   14  12   7     1.400   120.119     0.271  0.154657
  17  C5    c3    M   16  14  12     1.497   119.931   177.593 -0.173046
  18  H3    h1    E   17  16  14     1.130   109.498    -7.486  0.098448
  19  H4    h1    E   17  16  14     1.130   109.064  -125.961  0.098448
  20  N1    n     M   17  16  14     1.443   115.892   113.759 -0.287096
  21  C16   c     S   20  17  16     1.411   124.726   111.577  0.631126
  22  O1    o     E   21  20  17     1.236   124.502     7.502 -0.563613
  23  C4    ca    M   20  17  16     1.407   125.169   -58.674  0.268079
  24  C3    ca    M   23  20  17     1.396   129.341    -6.220 -0.246818
  25  H2    ha    E   24  23  20     1.102   120.814     4.016  0.158296
  26  C2    ca    M   24  23  20     1.398   118.309  -177.856  0.006033
  27  H1    ha    E   26  24  23     1.103   118.407  -179.576  0.128038
  28  C1    ca    M   26  24  23     1.395   121.385    -0.060 -0.152652
  29  Br1   br    E   28  26  24     1.874   119.994   179.392 -0.099292
  30  C13   ca    M   28  26  24     1.405   120.701    -0.889 -0.021039
  31  H9    ha    E   30  28  26     1.100   120.954  -178.920  0.138061
  32  C14   ca    M   30  28  26     1.379   118.681     0.844 -0.269317
  33  C15   c3    M   32  30  28     1.509   130.196  -179.226  0.238964
  34  C20   c3    3   33  32  30     1.523   111.233    62.598 -0.277849
  35  C19   c3    B   34  33  32     1.530   111.827  -175.163  0.154900
  36  H14   h1    E   35  34  33     1.131   108.119   -69.802  0.021057
  37  H15   h1    E   35  34  33     1.128   108.522   172.277  0.021057
  38  H16   hc    E   34  33  32     1.120   110.060    64.390  0.104373
  39  H17   hc    E   34  33  32     1.122   108.395   -53.472  0.104373
  40  C17   c3    M   33  32  30     1.522   111.655   -61.965 -0.277849
  41  H10   hc    E   40  33  32     1.122   108.359    53.453  0.104373
  42  H11   hc    E   40  33  32     1.120   110.111   -64.439  0.104373
  43  C18   c3    M   40  33  32     1.530   111.871   175.250  0.154900
  44  H12   h1    E   43  40  33     1.128   108.892  -173.251  0.021057
  45  H13   h1    E   43  40  33     1.131   108.056    68.849  0.021057
  46  N2    n3    M   43  40  33     1.444   113.405   -51.681 -0.303192
  47  S1    sy    M   46  43  40     1.612   123.590  -127.130  1.030776
  48  O2    o     E   47  46  43     1.405   111.002   132.029 -0.562439
  49  O3    o     E   47  46  43     1.411   111.704     5.063 -0.562439
  50  C21   ca    M   47  46  43     1.745   102.248  -110.676  0.155837
  51  C22   ca    M   50  47  46     1.408   124.446  -105.739 -0.075519
  52  Cl1   cl    E   51  50  47     1.698   122.958     9.366 -0.049597
  53  C23   ca    M   51  50  47     1.402   122.137  -172.329 -0.076676
  54  H18   ha    E   53  51  50     1.102   119.911   178.315  0.129457
  55  C24   ca    M   53  51  50     1.390   120.175    -2.356 -0.136592
  56  H19   ha    E   55  53  51     1.101   120.322   179.562  0.157791
  57  C26   ca    M   55  53  51     1.392   119.413    -1.507 -0.076676
  58  H20   ha    E   57  55  53     1.101   120.073  -177.690  0.129457
  59  C25   ca    M   57  55  53     1.397   119.744     1.688 -0.075519
  60  Cl2   cl    M   59  57  55     1.702   115.577  -176.577 -0.049597
```

```
LOOP
   C6   C9
  C15  C16
  C14   C4
   N2  C19
  C25  C21

IMPROPER
  C11   O4  C12   O5
  C12   C8  C11  C10
  C11   C9  C10   H8
   C6  C10   C9   H7
   C7  C11   C8   H6
   C8   C6   C7   H5
   C5   C7   C6   C9
  C16   C5   N1   C4
  C15   N1  C16   O1
   C3  C14   C4   N1
   C4   C2   C3   H2
   C3   C1   C2   H1
  Br1   C2   C1  C13
   C1  C14  C13   H9
  C15   C4  C14  C13
  C22  C25  C21   S1
  C21  C23  C22  Cl1
  C22  C24  C23  H18
  C23  C26  C24  H19
  C24  C25  C26  H20
  C21  C26  C25  Cl2

DONE
STOP
```

l79.in

```
    0    0    2

This is a remark line
molecule.res
L79   INT  0
CORRECT     OMIT DU   BEG
  0.0000
   1  DUMM  DU    M    0  -1  -2     0.000      .0        .0      .00000
   2  DUMM  DU    M    1   0  -1     1.449      .0        .0      .00000
   3  DUMM  DU    M    2   1   0     1.523   111.21       .0      .00000
   4  O4    o     M    3   2   1     1.540   111.208  -180.000 -0.797288
   5  C24   c     M    4   3   2     1.264   106.448   -54.973  0.817271
   6  O5    o     E    5   4   3     1.262   124.540    -1.735 -0.797288
   7  C8    ca    M    5   4   3     1.523   117.512   178.641  0.001774
   8  C11   ca    B    7   5   4     1.398   120.906   173.433 -0.147768
   9  C10   ca    B    8   7   5     1.395   120.352  -179.373 -0.145826
  10  C9    ca    S    9   8   7     1.394   120.217     0.174 -0.258107
  11  H6    ha    E   10   9   8     1.098   120.286  -179.884  0.144675
  12  H7    ha    E    9   8   7     1.099   120.046  -179.938  0.116162
  13  H20   ha    E    8   7   5     1.103   118.258     0.785  0.135060
  14  C7    ca    M    7   5   4     1.398   119.807    -6.095 -0.248344
  15  H5    ha    E   14   7   5     1.104   117.931    -1.196  0.137458
  16  C6    ca    M   14   7   5     1.398   120.560   179.101  0.197066
  17  C5    c3    M   16  14   7     1.498   119.750   178.820 -0.216834
  18  H3    h1    E   17  16  14     1.130   109.202  -129.463  0.122348
  19  H4    h1    E   17  16  14     1.129   109.228   -10.925  0.122348
  20  N1    n     M   17  16  14     1.443   115.649   110.208 -0.342495
  21  C15   c     S   20  17  16     1.408   124.867   111.450  0.692478
  22  O1    o     E   21  20  17     1.239   124.502     6.273 -0.574413
  23  C4    ca    M   20  17  16     1.409   125.120   -60.043  0.324498
  24  C3    ca    M   23  20  17     1.396   129.416    -5.212 -0.316957
  25  H2    ha    E   24  23  20     1.104   121.194     3.418  0.210365
  26  C2    ca    M   24  23  20     1.398   118.305  -178.243  0.037055
  27  H1    ha    E   26  24  23     1.102   118.312  -179.542  0.131701
  28  C1    ca    M   26  24  23     1.396   121.300    -0.027 -0.161022
  29  Br1   br    E   28  26  24     1.875   120.019   179.555 -0.104505
  30  C12   ca    M   28  26  24     1.404   120.753    -0.680 -0.033471
  31  H8    ha    E   30  28  26     1.101   120.977  -179.201  0.140048
  32  C13   ca    M   30  28  26     1.378   118.664     0.569 -0.242390
  33  C14   c3    M   32  30  28     1.510   130.122  -179.363  0.113633
  34  C19   c3    3   33  32  30     1.522   111.349    62.128 -0.211314
  35  C18   c3    B   34  33  32     1.530   111.821  -175.734  0.077234
  36  H13   h1    E   35  34  33     1.129   108.433   171.900  0.031175
  37  H14   h1    E   35  34  33     1.131   107.984   -70.316  0.031175
  38  H15   hc    E   34  33  32     1.121   110.064    63.834  0.096270
  39  H16   hc    E   34  33  32     1.122   108.331   -54.063  0.096270
  40  C16   c3    M   33  32  30     1.521   111.614   -62.253 -0.211314
  41  H9    hc    E   40  33  32     1.122   108.387    54.200  0.096270
  42  H10   hc    E   40  33  32     1.120   110.164   -63.744  0.096270
  43  C17   c3    M   40  33  32     1.530   111.755   175.855  0.077234
  44  H11   h1    E   43  40  33     1.129   108.504  -172.749  0.031175
  45  H12   h1    E   43  40  33     1.131   107.818    69.531  0.031175
  46  N2    n3    M   43  40  33     1.442   113.690   -51.370 -0.222658
  47  S1    sy    M   46  43  40     1.620   123.074  -133.174  1.006203
  48  O2    o     E   47  46  43     1.414   110.500   146.713 -0.556451
  49  O3    o     E   47  46  43     1.415   109.701    17.889 -0.556451
  50  C20   cc    M   47  46  43     1.672   103.742   -97.868 -0.118275
  51  C21   cd    M   50  47  46     1.403   120.090  -111.318 -0.023336
  52  H17   ha    E   51  50  47     1.097   123.535     0.870  0.155548
  53  C22   cd    M   51  50  47     1.414   112.239  -178.704 -0.290429
  54  H18   ha    E   53  51  50     1.091   124.019  -179.969  0.182324
  55  C23   cc    M   53  51  50     1.392   110.740    -0.058 -0.110069
  56  H19   h4    E   55  53  51     1.089   124.313  -179.742  0.192558
  57  S2    ss    M   55  53  51     1.638   111.626     0.123  0.042185


LOOP
   C6   C9
```

```
   C14  C15
   C13   C4
    N2  C18
    S2  C20

IMPROPER
    C8   O4  C24   O5
   C24   C7   C8  C11
    C8  C10  C11  H20
   C11   C9  C10   H7
    C6  C10   C9   H6
    C6   C8   C7   H5
    C5   C7   C6   C9
   C15   C5   N1   C4
   C14   N1  C15   O1
    C3  C13   C4   N1
    C4   C2   C3   H2
    C3   C1   C2   H1
   Br1   C2   C1  C12
    C1  C13  C12   H8
   C14   C4  C13  C12
   C21   S2  C20   S1
   C20  C22  C21  H17
   C23  C21  C22  H18
   C22  H19  C23   S2

DONE
STOP
```

l81.in

```
    0    0    2

This is a remark line
molecule.res
L81   INT  0
CORRECT     OMIT DU   BEG
  0.0000
   1  DUMM  DU    M    0  -1  -2     0.000      .0        .0      .00000
   2  DUMM  DU    M    1   0  -1     1.449      .0        .0      .00000
   3  DUMM  DU    M    2   1   0     1.523   111.21       .0      .00000
   4  O4    o     M    3   2   1     1.540   111.208  -180.000 -0.797082
   5  C12   c     M    4   3   2     1.263   163.040   -83.454  0.816769
   6  O5    o     E    5   4   3     1.262   124.529    25.786 -0.797082
   7  C11   ca    M    5   4   3     1.524   117.695  -154.267 -0.020262
   8  C10   ca    B    7   5   4     1.398   120.511   179.660 -0.129410
   9  C9    ca    S    8   7   5     1.393   120.526   179.909 -0.237931
  10  H7    ha    E    9   8   7     1.099   120.156  -179.495  0.134024
  11  H23   ha    E    8   7   5     1.102   118.225     0.126  0.134966
  12  C8    ca    M    7   5   4     1.399   120.314    -0.457 -0.129410
  13  H6    ha    E   12   7   5     1.102   118.166    -0.089  0.134966
  14  C7    ca    M   12   7   5     1.393   120.514   179.816 -0.237931
  15  H5    ha    E   14  12   7     1.100   119.932   179.646  0.134024
  16  C6    ca    M   14  12   7     1.400   120.120     0.331  0.144401
  17  C5    c3    M   16  14  12     1.497   119.929   177.741 -0.141939
  18  H3    h1    E   17  16  14     1.130   109.398    -7.249  0.089728
  19  H4    h1    E   17  16  14     1.130   109.078  -125.623  0.089728
  20  N1    n     M   17  16  14     1.443   115.856   114.020 -0.289983
  21  C16   c     S   20  17  16     1.410   124.723   113.760  0.626854
  22  O1    o     E   21  20  17     1.238   124.504     7.349 -0.558491
  23  C4    ca    M   20  17  16     1.407   125.253   -57.127  0.278957
  24  C3    ca    M   23  20  17     1.395   129.368    -5.873 -0.250804
  25  H2    ha    E   24  23  20     1.102   120.822     4.001  0.160056
  26  C2    ca    M   24  23  20     1.398   118.365  -177.948  0.007580
  27  H1    ha    E   26  24  23     1.102   118.467  -179.480  0.128895
  28  C1    ca    M   26  24  23     1.396   121.316    -0.033 -0.155490
  29  Br1   br    E   28  26  24     1.874   119.987   179.408 -0.099197
  30  C13   ca    M   28  26  24     1.405   120.686    -0.898 -0.018187
  31  H8    ha    E   30  28  26     1.102   120.917  -178.924  0.136982
  32  C14   ca    M   30  28  26     1.378   118.722     0.833 -0.267011
  33  C15   c3    M   32  30  28     1.510   130.196  -179.235  0.200467
  34  C20   c3    3   33  32  30     1.523   111.220    62.683 -0.225213
  35  C19   c3    B   34  33  32     1.529   111.775  -175.626  0.068130
  36  H13   h1    E   35  34  33     1.131   108.084   -70.101  0.044451
  37  H14   h1    E   35  34  33     1.128   108.492   172.033  0.044451
  38  H15   hc    E   34  33  32     1.121   110.099    63.952  0.094406
  39  H16   hc    E   34  33  32     1.122   108.360   -53.911  0.094406
  40  C17   c3    M   33  32  30     1.521   111.708   -61.769 -0.225213
  41  H9    hc    E   40  33  32     1.121   108.371    53.703  0.094406
  42  H10   hc    E   40  33  32     1.121   110.119   -64.151  0.094406
  43  C18   c3    M   40  33  32     1.530   111.897   175.490  0.068130
  44  H11   h1    E   43  40  33     1.129   108.564  -172.659  0.044451
  45  H12   h1    E   43  40  33     1.132   108.166    69.591  0.044451
  46  N2    n3    M   43  40  33     1.443   113.634   -51.194 -0.281406
  47  S1    sy    M   46  43  40     1.617   123.297  -130.391  1.132722
  48  O2    o     E   47  46  43     1.414   110.577   135.555 -0.585633
  49  O3    o     E   47  46  43     1.414   110.448     7.297 -0.585633
  50  C21   ca    M   47  46  43     1.710   103.531  -110.049 -0.262289
  51  C22   ca    M   50  47  46     1.404   116.188  -112.667 -0.064772
  52  H17   ha    E   51  50  47     1.107   119.134     0.352  0.172759
  53  C23   ca    M   51  50  47     1.390   121.595  -179.520 -0.249451
  54  H18   ha    E   53  51  50     1.101   120.253   179.896  0.165641
  55  C24   ca    M   53  51  50     1.392   119.680    -0.349 -0.024261
  56  H19   ha    E   55  53  51     1.101   120.234  -179.493  0.138045
  57  C26   ca    M   55  53  51     1.396   119.472     0.320 -0.101354
  58  Cl1   cl    E   57  55  53     1.704   117.824  -179.442 -0.100877
  59  C25   ca    M   57  55  53     1.408   121.277     0.206  0.390625
  60  C27   c3    M   59  57  55     1.483   119.180  -179.836 -0.543710
  61  H20   hc    E   60  59  57     1.118   112.358  -161.285  0.156715
  62  H21   hc    E   60  59  57     1.121   109.491    77.803  0.156715
  63  H22   hc    E   60  59  57     1.120   110.583   -41.400  0.156715
```

```
LOOP
   C6   C9
  C15  C16
  C14   C4
   N2  C19
  C25  C21

IMPROPER
  C11   O4  C12   O5
  C12   C8  C11  C10
  C11   C9  C10  H23
   C6  C10   C9   H7
   C7  C11   C8   H6
   C8   C6   C7   H5
   C5   C7   C6   C9
  C16   C5   N1   C4
  C15   N1  C16   O1
   C3  C14   C4   N1
   C4   C2   C3   H2
   C3   C1   C2   H1
  Br1   C2   C1  C13
   C1  C14  C13   H8
  C15   C4  C14  C13
  C22  C25  C21   S1
  C21  C23  C22  H17
  C22  C24  C23  H18
  C23  C26  C24  H19
  C24  C25  C26  Cl1
  C27  C21  C25  C26

DONE
STOP
```

l82.in

```
    0    0    2

This is a remark line
molecule.res
L82   INT  0
CORRECT     OMIT DU   BEG
  0.0000
   1  DUMM  DU    M    0  -1  -2     0.000      .0        .0      .00000
   2  DUMM  DU    M    1   0  -1     1.449      .0        .0      .00000
   3  DUMM  DU    M    2   1   0     1.523   111.21       .0      .00000
   4  O4    o     M    3   2   1     1.540   111.208  -180.000 -0.796957
   5  C12   c     M    4   3   2     1.263   161.946   -79.551  0.816072
   6  O5    o     E    5   4   3     1.263   124.535    21.174 -0.796957
   7  C11   ca    M    5   4   3     1.523   117.709  -158.779 -0.019097
   8  C10   ca    B    7   5   4     1.399   120.467   179.791 -0.130173
   9  C9    ca    S    8   7   5     1.393   120.502   179.909 -0.240192
  10  H7    ha    E    9   8   7     1.099   120.265  -179.487  0.135702
  11  H8    ha    E    8   7   5     1.102   118.225    -0.003  0.134561
  12  C8    ca    M    7   5   4     1.398   120.377    -0.381 -0.130173
  13  H6    ha    E   12   7   5     1.103   118.160    -0.082  0.134561
  14  C7    ca    M   12   7   5     1.393   120.584   179.842 -0.240192
  15  H5    ha    E   14  12   7     1.099   119.962   179.676  0.135702
  16  C6    ca    M   14  12   7     1.399   120.084     0.375  0.149917
  17  C5    c3    M   16  14  12     1.496   120.005   177.818 -0.169789
  18  H3    h1    E   17  16  14     1.130   109.477    -7.970  0.100393
  19  H4    h1    E   17  16  14     1.131   109.065  -126.421  0.100393
  20  N1    n     M   17  16  14     1.443   115.892   113.313 -0.289747
  21  C16   c     S   20  17  16     1.410   124.700   109.581  0.617040
  22  O1    o     E   21  20  17     1.237   124.573     8.625 -0.559794
  23  C4    ca    M   20  17  16     1.407   125.022   -59.233  0.281298
  24  C3    ca    M   23  20  17     1.396   129.339    -7.409 -0.256877
  25  H2    ha    E   24  23  20     1.101   120.847     4.169  0.161562
  26  C2    ca    M   24  23  20     1.398   118.298  -177.757  0.002249
  27  H1    ha    E   26  24  23     1.103   118.385  -179.566  0.130270
  28  C1    ca    M   26  24  23     1.395   121.383     0.034 -0.149346
  29  Br1   br    E   28  26  24     1.874   119.999   179.409 -0.099858
  30  C13   ca    M   28  26  24     1.405   120.699    -1.034 -0.020369
  31  H9    ha    E   30  28  26     1.100   120.911  -178.905  0.135972
  32  C14   ca    M   30  28  26     1.379   118.684     0.933 -0.271742
  33  C15   c3    M   32  30  28     1.509   130.172  -179.274  0.238148
  34  C20   c3    3   33  32  30     1.523   111.259    62.778 -0.233374
  35  C19   c3    B   34  33  32     1.529   111.859  -175.429  0.078103
  36  H14   h1    E   35  34  33     1.131   108.066   -69.472  0.049673
  37  H15   h1    E   35  34  33     1.128   108.538   172.557  0.049673
  38  H16   hc    E   34  33  32     1.121   110.059    64.144  0.094046
  39  H17   hc    E   34  33  32     1.122   108.376   -53.601  0.094046
  40  C17   c3    M   33  32  30     1.522   111.663   -61.863 -0.233374
  41  H10   hc    E   40  33  32     1.122   108.368    53.410  0.094046
  42  H11   hc    E   40  33  32     1.120   110.147   -64.490  0.094046
  43  C18   c3    M   40  33  32     1.530   111.868   175.168  0.078103
  44  H12   h1    E   43  40  33     1.129   108.922  -172.741  0.049673
  45  H13   h1    E   43  40  33     1.131   108.269    69.537  0.049673
  46  N2    n3    M   43  40  33     1.444   113.555   -51.271 -0.310327
  47  S1    sy    M   46  43  40     1.609   123.148  -130.161  1.141106
  48  O2    o     E   47  46  43     1.415   110.944   153.151 -0.577205
  49  O3    o     E   47  46  43     1.411   111.150    22.881 -0.577205
  50  C21   ca    M   47  46  43     1.720   103.485   -92.788 -0.281184
  51  C22   ca    M   50  47  46     1.400   117.519  -115.357 -0.035217
  52  H18   ha    E   51  50  47     1.108   118.866     1.367  0.173437
  53  C23   ca    M   51  50  47     1.390   121.654  -178.407 -0.261670
  54  H19   ha    E   53  51  50     1.101   120.100  -179.849  0.172463
  55  C24   ca    M   53  51  50     1.393   120.030    -0.011 -0.011134
  56  H20   ha    E   55  53  51     1.102   120.147  -179.834  0.142823
  57  C26   ca    M   55  53  51     1.396   120.047    -0.056 -0.117529
  58  Cl1   cl    E   57  55  53     1.698   118.965   179.964 -0.071855
  59  C25   ca    M   57  55  53     1.415   119.704    -0.129  0.471404
  60  F1    f     M   59  57  55     1.352   120.056  -179.411 -0.224818
```

```
LOOP
   C6   C9
  C15  C16
  C14   C4
   N2  C19
  C25  C21

IMPROPER
  C11   O4  C12   O5
  C12   C8  C11  C10
  C11   C9  C10   H8
   C6  C10   C9   H7
   C7  C11   C8   H6
   C8   C6   C7   H5
   C5   C7   C6   C9
  C16   C5   N1   C4
  C15   N1  C16   O1
   C3  C14   C4   N1
   C4   C2   C3   H2
   C3   C1   C2   H1
  Br1   C2   C1  C13
   C1  C14  C13   H9
  C15   C4  C14  C13
  C22  C25  C21   S1
  C21  C23  C22  H18
  C22  C24  C23  H19
  C23  C26  C24  H20
  C24  C25  C26  Cl1
  C21  C26  C25   F1

DONE
STOP
```

l83.in

```
    0    0    2

This is a remark line
molecule.res
L83   INT  0
CORRECT     OMIT DU   BEG
  0.0000
   1  DUMM  DU    M    0  -1  -2     0.000      .0        .0       .00000
   2  DUMM  DU    M    1   0  -1     1.449      .0        .0       .00000
   3  DUMM  DU    M    2   1   0     1.523   111.21       .0       .00000
   4  O4    o     M    3   2   1     1.540   111.208  -180.000 -0.795678
   5  C12   c     M    4   3   2     1.262    68.969   126.753  0.813836
   6  O5    o     E    5   4   3     1.263   124.544    -2.600 -0.795678
   7  C11   ca    M    5   4   3     1.524   117.749   177.436 -0.019760
   8  C10   ca    B    7   5   4     1.398   120.537    -0.642 -0.127684
   9  C9    ca    S    8   7   5     1.393   120.532   179.875 -0.241862
  10  H7    ha    E    9   8   7     1.099   120.185  -179.484  0.135819
  11  H8    ha    E    8   7   5     1.102   118.229     0.076  0.134230
  12  C8    ca    M    7   5   4     1.399   120.295   179.265 -0.127684
  13  H6    ha    E   12   7   5     1.102   118.180    -0.130  0.134230
  14  C7    ca    M   12   7   5     1.392   120.523   179.856 -0.241862
  15  H5    ha    E   14  12   7     1.099   119.952   179.595  0.135819
  16  C6    ca    M   14  12   7     1.400   120.097     0.342  0.151484
  17  C5    c3    M   16  14  12     1.498   119.872   177.631 -0.188173
  18  H3    h1    E   17  16  14     1.130   109.415    -7.107  0.105173
  19  H4    h1    E   17  16  14     1.130   109.032  -125.439  0.105173
  20  N1    n     M   17  16  14     1.443   115.899   114.201 -0.263441
  21  C16   c     S   20  17  16     1.409   124.691   115.096  0.641713
  22  O1    o     E   21  20  17     1.237   124.575     6.329 -0.575541
  23  C4    ca    M   20  17  16     1.406   125.304   -56.674  0.241438
  24  C3    ca    M   23  20  17     1.396   129.439    -5.041 -0.237996
  25  H2    ha    E   24  23  20     1.102   120.807     3.808  0.159937
  26  C2    ca    M   24  23  20     1.398   118.369  -178.100 -0.008851
  27  H1    ha    E   26  24  23     1.103   118.440  -179.528  0.132437
  28  C1    ca    M   26  24  23     1.395   121.367     0.016 -0.142205
  29  Br1   br    E   28  26  24     1.874   120.009   179.391 -0.099915
  30  C13   ca    M   28  26  24     1.404   120.692    -0.918 -0.037642
  31  H9    ha    E   30  28  26     1.101   120.957  -178.950  0.139155
  32  C14   ca    M   30  28  26     1.379   118.695     0.827 -0.215953
  33  C15   c3    M   32  30  28     1.510   130.168  -179.323  0.170530
  34  C20   c3    3   33  32  30     1.522   111.274    62.597 -0.226073
  35  C19   c3    B   34  33  32     1.530   111.869  -175.113  0.111541
  36  H14   h1    E   35  34  33     1.132   108.153   -69.717  0.039497
  37  H15   h1    E   35  34  33     1.128   108.532   172.279  0.039497
  38  H16   hc    E   34  33  32     1.121   110.053    64.504  0.089383
  39  H17   hc    E   34  33  32     1.121   108.418   -53.311  0.089383
  40  C17   c3    M   33  32  30     1.521   111.609   -62.047 -0.226073
  41  H10   hc    E   40  33  32     1.122   108.368    53.446  0.089383
  42  H11   hc    E   40  33  32     1.120   110.115   -64.428  0.089383
  43  C18   c3    M   40  33  32     1.530   111.802   175.248  0.111541
  44  H12   h1    E   43  40  33     1.129   108.908  -173.402  0.039497
  45  H13   h1    E   43  40  33     1.131   107.998    68.696  0.039497
  46  N2    n3    M   43  40  33     1.444   113.419   -51.771 -0.292592
  47  S1    sy    M   46  43  40     1.610   123.603  -126.374  1.077533
  48  O2    o     E   47  46  43     1.414   110.783   136.587 -0.575950
  49  O3    o     E   47  46  43     1.411   111.249     7.181 -0.575950
  50  C21   ca    M   47  46  43     1.723   103.624  -109.940 -0.040563
  51  C22   ca    M   50  47  46     1.403   116.754  -115.802 -0.095881
  52  H18   ha    E   51  50  47     1.108   119.211     1.225  0.174036
  53  C23   ca    M   51  50  47     1.395   121.044  -178.524  0.024076
  54  Cl1   cl    E   53  51  50     1.698   119.891   179.870 -0.105331
  55  C24   ca    M   53  51  50     1.400   120.365    -0.240 -0.070979
  56  H19   ha    E   55  53  51     1.100   120.491  -179.696  0.145744
  57  C26   ca    M   55  53  51     1.390   119.297     0.164 -0.143331
  58  H20   ha    E   57  55  53     1.102   119.843  -179.876  0.153328
  59  C25   ca    M   57  55  53     1.400   120.064    -0.013  0.042212
  60  Cl2   cl    M   59  57  55     1.697   117.722  -179.985 -0.083859
```

```
LOOP
   C6   C9
  C15  C16
  C14   C4
   N2  C19
  C25  C21

IMPROPER
  C11   O4  C12   O5
  C12   C8  C11  C10
  C11   C9  C10   H8
   C6  C10   C9   H7
   C7  C11   C8   H6
   C8   C6   C7   H5
   C5   C7   C6   C9
  C16   C5   N1   C4
  C15   N1  C16   O1
   C3  C14   C4   N1
   C4   C2   C3   H2
   C3   C1   C2   H1
  Br1   C2   C1  C13
   C1  C14  C13   H9
  C15   C4  C14  C13
  C22  C25  C21   S1
  C21  C23  C22  H18
  C22  C24  C23  Cl1
  C23  C26  C24  H19
  C24  C25  C26  H20
  C21  C26  C25  Cl2

DONE
STOP
```

l84.in

```
    0    0    2

This is a remark line
molecule.res
L84   INT  0
CORRECT     OMIT DU   BEG
  0.0000
   1  DUMM  DU    M    0  -1  -2     0.000      .0        .0      .00000
   2  DUMM  DU    M    1   0  -1     1.449      .0        .0      .00000
   3  DUMM  DU    M    2   1   0     1.523   111.21       .0      .00000
   4  O4    o     M    3   2   1     1.540   111.208  -180.000 -0.798448
   5  C12   c     M    4   3   2     1.263   160.570   -61.727  0.819558
   6  O5    o     E    5   4   3     1.262   124.540     4.468 -0.798448
   7  C11   ca    M    5   4   3     1.524   117.682  -175.528 -0.021666
   8  C10   ca    B    7   5   4     1.399   120.494   179.557 -0.129936
   9  C9    ca    S    8   7   5     1.393   120.513   179.830 -0.240960
  10  H7    ha    E    9   8   7     1.100   120.226  -179.499  0.135367
  11  H8    ha    E    8   7   5     1.102   118.201    -0.059  0.134961
  12  C8    ca    M    7   5   4     1.399   120.328    -0.652 -0.129936
  13  H6    ha    E   12   7   5     1.102   118.194     0.018  0.134961
  14  C7    ca    M   12   7   5     1.393   120.512   179.921 -0.240960
  15  H5    ha    E   14  12   7     1.100   119.953   179.606  0.135367
  16  C6    ca    M   14  12   7     1.400   120.125     0.373  0.153014
  17  C5    c3    M   16  14  12     1.498   119.908   177.605 -0.170332
  18  H3    h1    E   17  16  14     1.130   109.432    -7.427  0.098180
  19  H4    h1    E   17  16  14     1.131   108.997  -125.830  0.098180
  20  N1    n     M   17  16  14     1.443   115.872   113.911 -0.282257
  21  C16   c     S   20  17  16     1.410   124.739   113.025  0.638983
  22  O1    o     E   21  20  17     1.238   124.467     6.889 -0.564889
  23  C4    ca    M   20  17  16     1.406   125.239   -57.960  0.268198
  24  C3    ca    M   23  20  17     1.396   129.348    -5.680 -0.252591
  25  H2    ha    E   24  23  20     1.101   120.798     3.926  0.159956
  26  C2    ca    M   24  23  20     1.398   118.308  -177.952  0.010600
  27  H1    ha    E   26  24  23     1.103   118.408  -179.554  0.127490
  28  C1    ca    M   26  24  23     1.396   121.335     0.010 -0.156328
  29  Br1   br    E   28  26  24     1.874   119.944   179.404 -0.100344
  30  C13   ca    M   28  26  24     1.404   120.712    -0.948 -0.027912
  31  H9    ha    E   30  28  26     1.100   120.927  -178.960  0.139853
  32  C14   ca    M   30  28  26     1.378   118.706     0.856 -0.240896
  33  C15   c3    M   32  30  28     1.510   130.172  -179.339  0.167442
  34  C20   c3    3   33  32  30     1.522   111.225    62.706 -0.202813
  35  C19   c3    B   34  33  32     1.529   111.859  -175.463  0.071130
  36  H14   h1    E   35  34  33     1.131   108.050   -69.715  0.046718
  37  H15   h1    E   35  34  33     1.129   108.493   172.307  0.046718
  38  H16   hc    E   34  33  32     1.121   110.083    64.109  0.087841
  39  H17   hc    E   34  33  32     1.122   108.360   -53.712  0.087841
  40  C17   c3    M   33  32  30     1.522   111.686   -61.864 -0.202813
  41  H10   hc    E   40  33  32     1.122   108.310    53.519  0.087841
  42  H11   hc    E   40  33  32     1.120   110.104   -64.330  0.087841
  43  C18   c3    M   40  33  32     1.529   111.870   175.243  0.071130
  44  H12   h1    E   43  40  33     1.129   108.958  -172.830  0.046718
  45  H13   h1    E   43  40  33     1.133   108.232    69.540  0.046718
  46  N2    n3    M   43  40  33     1.443   113.619   -51.299 -0.283085
  47  S1    sy    M   46  43  40     1.612   123.094  -131.060  1.093442
  48  O2    o     E   47  46  43     1.415   110.843   152.020 -0.573481
  49  O3    o     E   47  46  43     1.411   110.871    22.239 -0.573481
  50  C21   ca    M   47  46  43     1.712   103.492   -93.599 -0.215197
  51  C22   ca    M   50  47  46     1.403   117.813  -117.386 -0.046086
  52  H18   ha    E   51  50  47     1.107   118.674     1.301  0.166806
  53  C23   ca    M   51  50  47     1.390   121.860  -178.546 -0.238584
  54  H19   ha    E   53  51  50     1.100   120.027  -179.972  0.155743
  55  C24   ca    M   53  51  50     1.396   119.874    -0.109 -0.027562
  56  H20   ha    E   55  53  51     1.101   120.079  -179.752  0.140186
  57  C26   ca    M   55  53  51     1.389   120.164    -0.009 -0.322186
  58  H21   ha    E   57  55  53     1.100   121.143  -179.713  0.187719
  59  C25   ca    M   57  55  53     1.409   119.493    -0.003  0.418190
```

```
  60  F1    f     M   59  57  55     1.353   118.424  -179.684 -0.223504


LOOP
   C6   C9
  C15  C16
  C14   C4
   N2  C19
  C25  C21

IMPROPER
  C11   O4  C12   O5
  C12   C8  C11  C10
  C11   C9  C10   H8
   C6  C10   C9   H7
   C7  C11   C8   H6
   C8   C6   C7   H5
   C5   C7   C6   C9
  C16   C5   N1   C4
  C15   N1  C16   O1
   C3  C14   C4   N1
   C4   C2   C3   H2
   C3   C1   C2   H1
  Br1   C2   C1  C13
   C1  C14  C13   H9
  C15   C4  C14  C13
  C22  C25  C21   S1
  C21  C23  C22  H18
  C22  C24  C23  H19
  C23  C26  C24  H20
  C24  C25  C26  H21
  C21  C26  C25   F1

DONE
STOP
```

l85.in

```
    0    0    2

This is a remark line
molecule.res
L85   INT  0
CORRECT     OMIT DU   BEG
  0.0000
   1  DUMM  DU    M    0  -1  -2     0.000      .0        .0      .00000
   2  DUMM  DU    M    1   0  -1     1.449      .0        .0      .00000
   3  DUMM  DU    M    2   1   0     1.523   111.21       .0      .00000
   4  O4    o     M    3   2   1     1.540   111.208  -180.000 -0.791776
   5  C12   c     M    4   3   2     1.279   100.362  -119.112  0.794833
   6  O5    o     E    5   4   3     1.280   124.731   178.134 -0.791776
   7  C11   ca    M    5   4   3     1.528   117.677    -1.885 -0.018391
   8  C10   ca    B    7   5   4     1.399   120.544    -0.840 -0.121668
   9  C9    ca    S    8   7   5     1.393   120.560   179.894 -0.248709
  10  H7    ha    E    9   8   7     1.099   120.178  -179.570  0.135478
  11  H8    ha    E    8   7   5     1.103   118.212     0.026  0.134386
  12  C8    ca    M    7   5   4     1.399   120.328   179.054 -0.121668
  13  H6    ha    E   12   7   5     1.103   118.190    -0.135  0.134386
  14  C7    ca    M   12   7   5     1.393   120.530   179.869 -0.248709
  15  H5    ha    E   14  12   7     1.100   119.941   179.737  0.135478
  16  C6    ca    M   14  12   7     1.400   120.119     0.311  0.162866
  17  C5    c3    M   16  14  12     1.497   119.798   177.438 -0.176503
  18  H3    h1    E   17  16  14     1.129   109.423    -6.063  0.095620
  19  H4    h1    E   17  16  14     1.130   108.933  -124.232  0.095620
  20  N1    n     M   17  16  14     1.443   115.818   115.619 -0.228379
  21  C16   c     S   20  17  16     1.409   124.637   124.610  0.621609
  22  O1    o     E   21  20  17     1.239   124.439     1.363 -0.569689
  23  C4    ca    M   20  17  16     1.406   125.727   -54.174  0.211630
  24  C3    ca    M   23  20  17     1.396   129.494    -0.060 -0.219787
  25  H2    ha    E   24  23  20     1.103   120.753     3.002  0.156402
  26  C2    ca    M   24  23  20     1.397   118.356  -178.762 -0.006676
  27  H1    ha    E   26  24  23     1.103   118.399  -179.453  0.130825
  28  C1    ca    M   26  24  23     1.395   121.401     0.078 -0.143216
  29  Br1   br    E   28  26  24     1.874   120.039   179.424 -0.101715
  30  C13   ca    M   28  26  24     1.405   120.656    -0.803 -0.037880
  31  H9    ha    E   30  28  26     1.101   120.953  -179.145  0.138863
  32  C14   ca    M   30  28  26     1.379   118.695     0.724 -0.221963
  33  C15   c3    M   32  30  28     1.510   130.085  -179.378  0.175807
  34  C20   c3    3   33  32  30     1.522   111.234    63.002 -0.218573
  35  C19   c3    B   34  33  32     1.529   111.759  -175.341  0.094428
  36  H14   h1    E   35  34  33     1.131   108.073   -69.955  0.033926
  37  H15   h1    E   35  34  33     1.128   108.605   172.149  0.033926
  38  H16   hc    E   34  33  32     1.120   110.051    64.206  0.090362
  39  H17   hc    E   34  33  32     1.122   108.370   -53.682  0.090362
  40  C17   c3    M   33  32  30     1.522   111.654   -61.556 -0.218573
  41  H10   hc    E   40  33  32     1.122   108.343    53.530  0.090362
  42  H11   hc    E   40  33  32     1.120   110.084   -64.363  0.090362
  43  C18   c3    M   40  33  32     1.530   111.861   175.310  0.094428
  44  H12   h1    E   43  40  33     1.128   108.705  -172.898  0.033926
  45  H13   h1    E   43  40  33     1.132   107.948    69.258  0.033926
  46  N2    n3    M   43  40  33     1.443   113.508   -51.398 -0.284408
  47  S1    sy    M   46  43  40     1.620   123.399  -129.625  1.096063
  48  O2    o     E   47  46  43     1.416   110.356   135.936 -0.584113
  49  O3    o     E   47  46  43     1.415   110.195     7.545 -0.584113
  50  C21   ca    M   47  46  43     1.699   103.488  -109.513 -0.150515
  51  C22   ca    M   50  47  46     1.404   117.493   -98.997 -0.125778
  52  H18   ha    E   51  50  47     1.106   118.921     0.541  0.172201
  53  C23   ca    M   51  50  47     1.389   121.574  -178.669 -0.190585
  54  H19   ha    E   53  51  50     1.099   120.283  -179.938  0.143372
  55  C24   ca    M   53  51  50     1.397   119.495    -0.068 -0.052897
  56  H20   ha    E   55  53  51     1.101   120.055  -179.725  0.132134
  57  C26   ca    M   55  53  51     1.391   119.863     0.195 -0.294953
  58  H21   ha    E   57  55  53     1.101   119.803  -179.927  0.163483
```

```
  59  C25   ca    M   57  55  53     1.402   120.675    -0.289  0.277938
  60  C27   c3    M   59  57  55     1.484   118.775   179.349 -0.408863
  61  H22   hc    E   60  59  57     1.119   110.962   132.080  0.122292
  62  H23   hc    E   60  59  57     1.117   111.220    11.481  0.122292
  63  H24   hc    E   60  59  57     1.121   109.754  -108.962  0.122292


LOOP
   C6   C9
  C15  C16
  C14   C4
   N2  C19
  C25  C21

IMPROPER
  C11   O4  C12   O5
  C12   C8  C11  C10
  C11   C9  C10   H8
   C6  C10   C9   H7
   C7  C11   C8   H6
   C8   C6   C7   H5
   C5   C7   C6   C9
  C16   C5   N1   C4
  C15   N1  C16   O1
   C3  C14   C4   N1
   C4   C2   C3   H2
   C3   C1   C2   H1
  Br1   C2   C1  C13
   C1  C14  C13   H9
  C15   C4  C14  C13
  C22  C25  C21   S1
  C21  C23  C22  H18
  C22  C24  C23  H19
  C23  C26  C24  H20
  C24  C25  C26  H21
  C27  C21  C25  C26

DONE
STOP
```

l88.in

```
    0    0    2

This is a remark line
molecule.res
L88   INT  0
CORRECT     OMIT DU   BEG
  0.0000
   1  DUMM  DU    M    0  -1  -2     0.000      .0        .0      .00000
   2  DUMM  DU    M    1   0  -1     1.449      .0        .0      .00000
   3  DUMM  DU    M    2   1   0     1.523   111.21       .0      .00000
   4  O4    o     M    3   2   1     1.540   111.208  -180.000 -0.799232
   5  C12   c     M    4   3   2     1.263   121.028  -142.622  0.821294
   6  O5    o     E    5   4   3     1.263   124.478  -172.800 -0.799232
   7  C11   ca    M    5   4   3     1.523   117.764     7.217 -0.029287
   8  C10   ca    B    7   5   4     1.400   120.407     1.457 -0.125101
   9  C9    ca    S    8   7   5     1.392   120.518   179.935 -0.240400
  10  H7    ha    E    9   8   7     1.101   120.554  -179.785  0.129130
  11  H8    ha    E    8   7   5     1.102   118.151     0.016  0.133798
  12  C8    ca    M    7   5   4     1.399   120.451  -178.554 -0.125101
  13  H6    ha    E   12   7   5     1.103   118.124    -0.127  0.133798
  14  C7    ca    M   12   7   5     1.392   120.565   179.900 -0.240400
  15  H5    ha    E   14  12   7     1.100   120.049  -179.954  0.129130
  16  C6    ca    M   14  12   7     1.398   120.047     0.301  0.144469
  17  C5    c3    M   16  14  12     1.497   120.493   178.187 -0.110594
  18  H3    h1    E   17  16  14     1.128   109.882   -15.126  0.088819
  19  H4    h1    E   17  16  14     1.130   108.977  -134.128  0.088819
  20  N1    n     M   17  16  14     1.445   115.752   106.316 -0.406356
  21  C16   c     S   20  17  16     1.415   123.756    84.665  0.650712
  22  O1    o     E   21  20  17     1.236   124.448    16.832 -0.534652
  23  C4    ca    M   20  17  16     1.408   124.284   -74.366  0.400394
  24  C3    ca    M   23  20  17     1.396   129.305   -15.862 -0.354119
  25  H2    ha    E   24  23  20     1.100   121.070     4.626  0.189807
  26  C2    ca    M   24  23  20     1.398   118.458  -176.674  0.044414
  27  H1    ha    E   26  24  23     1.102   118.506  -179.767  0.119897
  28  C1    ca    M   26  24  23     1.395   121.298    -0.089 -0.169083
  29  Br1   br    E   28  26  24     1.875   119.973   179.460 -0.097120
  30  C13   ca    M   28  26  24     1.405   120.677    -0.836  0.012528
  31  H9    ha    E   30  28  26     1.101   120.872  -178.932  0.134585
  32  C14   ca    M   30  28  26     1.378   118.721     0.817 -0.349929
  33  C15   c3    M   32  30  28     1.509   130.109  -179.103  0.285647
  34  C20   c3    3   33  32  30     1.522   111.132    63.050 -0.288853
  35  C19   c3    B   34  33  32     1.528   111.829  -175.934  0.076194
  36  H14   h1    E   35  34  33     1.131   108.070   -69.705  0.040710
  37  H15   h1    E   35  34  33     1.129   108.470   172.460  0.040710
  38  H16   hc    E   34  33  32     1.121   110.092    63.670  0.117647
  39  H17   hc    E   34  33  32     1.122   108.388   -54.174  0.117647
  40  C17   c3    M   33  32  30     1.522   111.866   -61.465 -0.288853
  41  H10   hc    E   40  33  32     1.122   108.435    53.953  0.117647
  42  H11   hc    E   40  33  32     1.120   110.188   -64.053  0.117647
  43  C18   c3    M   40  33  32     1.530   111.728   175.657  0.076194
  44  H12   h1    E   43  40  33     1.129   108.756  -172.875  0.040710
  45  H13   h1    E   43  40  33     1.131   107.986    69.268  0.040710
  46  N2    n3    M   43  40  33     1.444   113.544   -51.498 -0.262925
  47  S1    sy    M   46  43  40     1.611   122.924  -132.284  1.002326
  48  O2    o     E   47  46  43     1.413   110.423   158.472 -0.543813
  49  O3    o     E   47  46  43     1.408   111.407    27.204 -0.543813
  50  C21   ca    M   47  46  43     1.728   103.072   -88.040  0.027304
  51  C22   ca    M   50  47  46     1.403   114.574   -75.596 -0.170738
  52  H18   ha    E   51  50  47     1.106   118.880    -0.047  0.157591
  53  C23   ca    M   51  50  47     1.392   121.965  -179.279 -0.078328
  54  H19   ha    E   53  51  50     1.101   120.028  -179.679  0.145211
```

```
  55  C24   ca    M   53  51  50     1.392   119.462     0.079 -0.137409
  56  H20   ha    E   55  53  51     1.101   120.480  -179.705  0.147478
  57  C26   ca    M   55  53  51     1.395   119.678     0.340 -0.101668
  58  H21   ha    E   57  55  53     1.102   119.457   179.786  0.136240
  59  C25   ca    M   57  55  53     1.398   120.472    -0.489 -0.074764
  60  C27   c3    M   59  57  55     1.538   116.579   179.428  0.626500
  61  F2    f     E   60  59  57     1.378   113.890     7.903 -0.221311
  62  F3    f     E   60  59  57     1.369   114.583  -110.733 -0.221311
  63  F1    f     M   60  59  57     1.372   114.744   126.229 -0.221311


LOOP
   C6   C9
  C15  C16
  C14   C4
   N2  C19
  C25  C21

IMPROPER
  C11   O4  C12   O5
  C12   C8  C11  C10
  C11   C9  C10   H8
   C6  C10   C9   H7
   C7  C11   C8   H6
   C8   C6   C7   H5
   C5   C7   C6   C9
  C16   C5   N1   C4
  C15   N1  C16   O1
   C3  C14   C4   N1
   C4   C2   C3   H2
   C3   C1   C2   H1
  Br1   C2   C1  C13
   C1  C14  C13   H9
  C15   C4  C14  C13
  C22  C25  C21   S1
  C21  C23  C22  H18
  C22  C24  C23  H19
  C23  C26  C24  H20
  C24  C25  C26  H21
  C27  C21  C25  C26

DONE
STOP
```

l89.in

```
    0    0    2

This is a remark line
molecule.res
L89   INT  0
CORRECT     OMIT DU   BEG
  0.0000
   1  DUMM  DU    M    0  -1  -2     0.000      .0        .0      .00000
   2  DUMM  DU    M    1   0  -1     1.449      .0        .0      .00000
   3  DUMM  DU    M    2   1   0     1.523   111.21       .0      .00000
   4  O4    o     M    3   2   1     1.540   111.208  -180.000 -0.795893
   5  C12   c     M    4   3   2     1.264   161.033   -83.429  0.814305
   6  O5    o     E    5   4   3     1.262   124.555    25.448 -0.795893
   7  C11   ca    M    5   4   3     1.524   117.674  -154.479 -0.017369
   8  C10   ca    B    7   5   4     1.398   120.518   179.471 -0.131670
   9  C9    ca    S    8   7   5     1.394   120.550   179.913 -0.236309
  10  H7    ha    E    9   8   7     1.099   120.186  -179.530  0.133983
  11  H8    ha    E    8   7   5     1.103   118.229    -0.031  0.135534
  12  C8    ca    M    7   5   4     1.399   120.323    -0.747 -0.131670
  13  H6    ha    E   12   7   5     1.103   118.145    -0.103  0.135534
  14  C7    ca    M   12   7   5     1.392   120.546   179.864 -0.236309
  15  H5    ha    E   14  12   7     1.100   119.948   179.687  0.133983
  16  C6    ca    M   14  12   7     1.400   120.074     0.363  0.140773
  17  C5    c3    M   16  14  12     1.497   119.848   177.662 -0.127555
  18  H3    h1    E   17  16  14     1.130   109.490    -7.025  0.085958
  19  H4    h1    E   17  16  14     1.130   109.044  -125.444  0.085958
  20  N1    n     M   17  16  14     1.444   115.875   114.255 -0.307466
  21  C16   c     S   20  17  16     1.409   124.713   115.195  0.627556
  22  O1    o     E   21  20  17     1.237   124.549     6.580 -0.558440
  23  C4    ca    M   20  17  16     1.407   125.313   -56.402  0.301137
  24  C3    ca    M   23  20  17     1.396   129.382    -5.266 -0.256903
  25  H2    ha    E   24  23  20     1.102   120.771     3.896  0.162565
  26  C2    ca    M   24  23  20     1.398   118.351  -177.990 -0.000902
  27  H1    ha    E   26  24  23     1.102   118.429  -179.481  0.131343
  28  C1    ca    M   26  24  23     1.396   121.335    -0.056 -0.142585
  29  Br1   br    E   28  26  24     1.874   119.986   179.413 -0.101184
  30  C13   ca    M   28  26  24     1.405   120.715    -0.850 -0.016543
  31  H9    ha    E   30  28  26     1.101   120.914  -179.036  0.135969
  32  C14   ca    M   30  28  26     1.379   118.661     0.813 -0.306811
  33  C15   c3    M   32  30  28     1.509   130.125  -179.303  0.219521
  34  C20   c3    3   33  32  30     1.523   111.290    62.683 -0.180005
  35  C19   c3    B   34  33  32     1.529   111.866  -175.315  0.033691
  36  H14   h1    E   35  34  33     1.131   108.060   -69.613  0.046005
  37  H15   h1    E   35  34  33     1.129   108.607   172.390  0.046005
  38  H16   hc    E   34  33  32     1.121   110.088    64.191  0.085703
  39  H17   hc    E   34  33  32     1.122   108.328   -53.572  0.085703
  40  C17   c3    M   33  32  30     1.522   111.664   -61.967 -0.180005
  41  H10   hc    E   40  33  32     1.122   108.335    53.487  0.085703
  42  H11   hc    E   40  33  32     1.120   110.123   -64.334  0.085703
  43  C18   c3    M   40  33  32     1.530   111.883   175.216  0.033691
  44  H12   h1    E   43  40  33     1.128   108.642  -172.763  0.046005
  45  H13   h1    E   43  40  33     1.132   107.980    69.225  0.046005
  46  N2    n3    M   43  40  33     1.443   113.503   -51.424 -0.267677
  47  S1    sy    M   46  43  40     1.616   123.015  -130.164  1.064033
  48  O2    o     E   47  46  43     1.413   110.467   153.150 -0.574200
  49  O3    o     E   47  46  43     1.415   110.325    23.046 -0.574200
  50  C21   ca    M   47  46  43     1.699   103.612   -91.886  0.016942
```

```
  51  C22   ca    M   50  47  46     1.400   120.621   -88.200 -0.197991
  52  H18   ha    E   51  50  47     1.105   119.259     0.228  0.169997
  53  C23   ca    M   51  50  47     1.391   121.108  -178.911 -0.060148
  54  H19   ha    E   53  51  50     1.101   120.176  -179.858  0.132331
  55  C24   ca    M   53  51  50     1.399   119.279     0.048  0.044065
  56  Cl1   cl    E   55  53  51     1.699   119.744  -179.911 -0.112157
  57  C26   ca    M   55  53  51     1.399   120.568     0.234 -0.060148
  58  H20   ha    E   57  55  53     1.100   120.560   179.759  0.132331
  59  C25   ca    M   57  55  53     1.392   119.233    -0.247 -0.197991
  60  H21   ha    E   59  57  55     1.105   119.616   179.089  0.169997


LOOP
   C6   C9
  C15  C16
  C14   C4
   N2  C19
  C25  C21

IMPROPER
  C11   O4  C12   O5
  C12   C8  C11  C10
  C11   C9  C10   H8
   C6  C10   C9   H7
   C7  C11   C8   H6
   C8   C6   C7   H5
   C5   C7   C6   C9
  C16   C5   N1   C4
  C15   N1  C16   O1
   C3  C14   C4   N1
   C4   C2   C3   H2
   C3   C1   C2   H1
  Br1   C2   C1  C13
   C1  C14  C13   H9
  C15   C4  C14  C13
  C22  C25  C21   S1
  C21  C23  C22  H18
  C22  C24  C23  H19
  C23  C26  C24  Cl1
  C24  C25  C26  H20
  C21  C26  C25  H21

DONE
STOP
```

l91.in

```
    0    0    2

This is a remark line
molecule.res
L91   INT  0
CORRECT     OMIT DU   BEG
  0.0000
   1  DUMM  DU    M    0  -1  -2     0.000      .0        .0      .00000
   2  DUMM  DU    M    1   0  -1     1.449      .0        .0      .00000
   3  DUMM  DU    M    2   1   0     1.523   111.21       .0      .00000
   4  C15   ca    M    3   2   1     1.540   111.208  -180.000 -0.055443
   5  C17   ca    S    4   3   2     1.390   120.584     2.587 -0.325294
   6  H9    ha    E    5   4   3     1.101   119.127   -68.037  0.184544
   7  H19   ha    E    4   3   2     1.101    57.954  -105.034  0.128155
   8  C10   ca    M    4   3   2     1.394    91.110   129.732 -0.213194
   9  H20   ha    E    8   4   3     1.099   120.279    53.015  0.139501
  10  C16   ca    M    8   4   3     1.393   119.371  -127.093 -0.055443
  11  H8    ha    E   10   8   4     1.102   119.935  -179.915  0.128155
  12  C18   ca    M   10   8   4     1.393   121.027     0.052 -0.325294
  13  H10   ha    E   12  10   8     1.103   119.174  -179.840  0.184544
  14  C14   ca    M   12  10   8     1.410   119.985    -0.030  0.423974
  15  N4    n     M   14  12  10     1.407   123.311   179.493 -0.744996
  16  H3    hn    E   15  14  12     0.997   115.493  -178.504  0.339663
  17  C3    c     M   15  14  12     1.384   127.215     5.618  1.050324
  18  O3    o     E   17  15  14     1.243   124.507    -1.662 -0.608713
  19  C1    cc    M   17  15  14     1.487   114.911   177.234 -0.542297
  20  N1    na    B   19  17  15     1.416   125.537    59.171  0.313629
  21  N2    nc    E   20  19  17     1.346   111.695  -179.499 -0.560386
  22  C8    ca    S   20  19  17     1.428   125.027    10.067  0.214580
  23  C19   ca    B   22  20  19     1.410   120.347    33.901 -0.239182
  24  C22   ca    B   23  22  20     1.391   119.585  -179.226 -0.078881
  25  C23   ca    B   24  23  22     1.394   120.654    -0.847 -0.187716
  26  C21   ca    B   25  24  23     1.394   119.804     0.043 -0.078881
  27  C20   ca    S   26  25  24     1.393   120.657     0.332 -0.239182
  28  H12   ha    E   27  26  25     1.102   119.777  -179.391  0.155653
  29  H13   ha    E   26  25  24     1.100   120.024  -179.495  0.136561
  30  H15   ha    E   25  24  23     1.100   120.117  -179.798  0.143803
  31  H14   ha    E   24  23  22     1.102   119.266   179.535  0.136561
  32  H11   ha    E   23  22  20     1.103   120.693     0.762  0.155653
  33  C2    cd    M   19  17  15     1.398   128.116  -118.750  0.186481
  34  C6    c3    B   33  19  17     1.478   132.310    -1.977 -0.323944
  35  H1    h1    E   34  33  19     1.133   108.211   -69.592  0.135846
  36  H2    h1    E   34  33  19     1.131   109.706    48.058  0.135846
  37  C4    cd    M   33  19  17     1.456   104.930   178.682  0.291507
  38  C7    c3    M   37  33  19     1.478   121.111  -178.588 -0.120806
  39  H4    hc    E   38  37  33     1.123   109.325   103.587  0.092930
  40  H5    hc    E   38  37  33     1.122   111.312  -137.402  0.092930
  41  C9    c3    M   38  37  33     1.539   109.773   -17.329 -0.037010
  42  H6    h1    E   41  38  37     1.129   107.932   166.076  0.064923
  43  H7    h1    E   41  38  37     1.129   107.988   -76.980  0.064923
  44  N3    n3    M   41  38  37     1.441   114.869    44.494 -0.160451
  45  S1    sy    M   44  41  38     1.636   122.722   118.450  1.064558
  46  O1    o     E   45  44  41     1.411   109.710  -142.600 -0.554420
  47  O2    o     E   45  44  41     1.412   108.987   -13.851 -0.554420
  48  C5    cc    M   45  44  41     1.663   103.512   101.840 -0.121272
  49  C11   cd    M   48  45  44     1.405   120.200   101.604 -0.038387
```

```
  50  H17   ha    E   49  48  45     1.097   123.566    -0.700  0.155434
  51  C13   cd    M   49  48  45     1.412   112.272   178.328 -0.257692
  52  H18   ha    E   51  49  48     1.092   124.031  -179.885  0.182642
  53  C12   cc    M   51  49  48     1.392   110.741    -0.070 -0.125629
  54  H16   h4    E   53  51  49     1.091   124.218  -179.948  0.201768
  55  S2    ss    M   53  51  49     1.637   111.717    -0.146  0.043843


LOOP
  C14  C17
   C4   N2
  C20   C8
   N3   C6
   S2   C5

IMPROPER
  C17  C10  C15  H19
  C14  C15  C17   H9
  C15  C16  C10  H20
  C18  C10  C16   H8
  C14  C16  C18  H10
  C18  C17  C14   N4
   C3  C14   N4   H3
   C1   N4   C3   O3
   C3   C2   C1   N1
   C8   C1   N1   N2
  C19  C20   C8   N1
   C8  C22  C19  H11
  C19  C23  C22  H14
  C22  C21  C23  H15
  C23  C20  C21  H13
   C8  C21  C20  H12
   C6   C1   C2   C4
   C7   C2   C4   N2
  C11   S2   C5   S1
   C5  C13  C11  H17
  C12  C11  C13  H18
  C13  H16  C12   S2

DONE
STOP
```

l93.in

```
    0    0    2

This is a remark line
molecule.res
L93   INT  0
CORRECT     OMIT DU   BEG
  0.0000
   1  DUMM  DU    M    0  -1  -2     0.000      .0        .0      .00000
   2  DUMM  DU    M    1   0  -1     1.449      .0        .0      .00000
   3  DUMM  DU    M    2   1   0     1.523   111.21       .0      .00000
   4  C16   ca    M    3   2   1     1.540   111.208  -180.000 -0.072306
   5  C18   ca    S    4   3   2     1.390   114.162     4.749 -0.285042
   6  H10   ha    E    5   4   3     1.101   119.169   -41.216  0.168681
   7  H8    ha    E    4   3   2     1.100    37.059  -102.632  0.130681
   8  C11   ca    M    4   3   2     1.395   112.512   147.038 -0.203556
   9  H17   ha    E    8   4   3     1.099   120.217    40.732  0.138010
  10  C17   ca    M    8   4   3     1.392   119.434  -139.355 -0.072306
  11  H9    ha    E   10   8   4     1.100   119.957  -179.930  0.130681
  12  C19   ca    M   10   8   4     1.393   120.982     0.067 -0.285042
  13  H11   ha    E   12  10   8     1.102   119.302  -179.724  0.168681
  14  C15   ca    M   12  10   8     1.409   119.947    -0.253  0.361321
  15  N3    n     M   14  12  10     1.409   123.330   178.387 -0.611585
  16  H3    hn    E   15  14  12     0.999   115.598  -167.193  0.305662
  17  C4    c     M   15  14  12     1.391   124.800    21.834  0.830571
  18  O3    o     E   17  15  14     1.250   123.675    -3.884 -0.554918
  19  N1    n     M   17  15  14     1.429   118.412   176.169 -0.189578
  20  C2    cc    B   19  17  15     1.418   129.648    16.782 -0.129649
  21  C3    cd    S   20  19  17     1.402   108.622  -173.263 -0.342725
  22  H21   ha    E   21  20  19     1.087   125.894  -179.024  0.201247
  23  C9    ca    S   20  19  17     1.453   125.784    10.227  0.283763
  24  C20   ca    B   23  20  19     1.402   121.191    53.184 -0.214263
  25  C23   ca    B   24  23  20     1.394   120.085   176.944 -0.125092
  26  C24   ca    B   25  24  23     1.395   120.220    -0.107 -0.149951
  27  C22   ca    B   26  25  24     1.395   119.886    -0.075 -0.125092
  28  C21   ca    S   27  26  25     1.393   120.206     0.125 -0.214263
  29  H13   ha    E   28  27  26     1.101   120.290  -179.585  0.147932
  30  H14   ha    E   27  26  25     1.101   119.982  -179.685  0.138106
  31  H16   ha    E   26  25  24     1.100   120.038  -179.817  0.138982
  32  H15   ha    E   25  24  23     1.100   119.753  -179.907  0.138106
  33  H12   ha    E   24  23  20     1.101   119.913    -2.539  0.147932
  34  C1    cc    M   19  17  15     1.406   122.644  -155.337  0.124104
  35  C7    c3    B   34  19  17     1.494   127.000    -7.117 -0.260074
  36  H1    h1    E   35  34  19     1.132   108.104   -69.532  0.122365
  37  H2    h1    E   35  34  19     1.133   109.969    47.852  0.122365
  38  C5    cd    M   34  19  17     1.401   109.125   173.990  0.045006
  39  C8    c3    M   38  34  19     1.468   121.751  -179.963 -0.044569
  40  H4    hc    E   39  38  34     1.124   109.700   106.050  0.063078
  41  H5    hc    E   39  38  34     1.122   110.856  -135.246  0.063078
  42  C10   c3    M   39  38  34     1.540   110.377   -15.180 -0.038411
  43  H6    h1    E   42  39  38     1.129   108.258   163.622  0.055114
  44  H7    h1    E   42  39  38     1.129   107.968   -79.337  0.055114
  45  N2    n3    M   42  39  38     1.442   114.702    41.964 -0.150592
  46  S1    sy    M   45  42  39     1.634   122.746   119.447  1.083719
  47  O1    o     E   46  45  42     1.410   109.997  -140.244 -0.561762
  48  O2    o     E   46  45  42     1.414   109.133   -11.364 -0.561762
  49  C6    cc    M   46  45  42     1.665   103.446   104.187 -0.127363
  50  C12   cd    M   49  46  45     1.404   120.262    97.698 -0.051498
  51  H18   ha    E   50  49  46     1.096   123.584    -0.723  0.162830
  52  C14   cd    M   50  49  46     1.413   112.225   178.363 -0.240068
```

```
  53  H20    ha    E   52  50  49     1.092   123.991  -179.943  0.177666
  54  C13    cc    M   52  50  49     1.391   110.789     0.000 -0.144987
  55  H19    h4    E   54  52  50     1.090   124.308  -179.923  0.202964
  56  S2     ss    M   54  52  50     1.638   111.704    -0.241  0.048696


LOOP
  C15  C18
   C5   C3
  C21   C9
   N2   C7
   S2   C6

IMPROPER
  C18  C11  C16   H8
  C15  C16  C18  H10
  C16  C17  C11  H17
  C19  C11  C17   H9
  C15  C17  C19  H11
  C19  C18  C15   N3
   C4  C15   N3   H3
   N3   N1   C4   O3
   C4   C2   N1   C1
   C9   C3   C2   N1
   C2   C5   C3  H21
  C20  C21   C9   C2
   C9  C23  C20  H12
  C20  C24  C23  H15
  C23  C22  C24  H16
  C24  C21  C22  H14
   C9  C22  C21  H13
   C7   C5   C1   N1
   C8   C1   C5   C3
  C12   S2   C6   S1
   C6  C14  C12  H18
  C13  C12  C14  H20
  C14  H19  C13   S2

DONE
STOP
```

l95.in

```
    0    0    2

This is a remark line
molecule.res
L95   INT  0
CORRECT     OMIT DU   BEG
  0.0000
   1  DUMM  DU    M    0  -1  -2     0.000      .0        .0      .00000
   2  DUMM  DU    M    1   0  -1     1.449      .0        .0      .00000
   3  DUMM  DU    M    2   1   0     1.523   111.21       .0      .00000
   4  O4    o     M    3   2   1     1.540   111.208  -180.000 -0.588633
   5  C18   c     M    4   3   2     1.236    80.214  -121.601  0.608135
   6  C26   c3    3    5   4   3     1.491   122.882    67.295 -0.404154
   7  H20   hc    E    6   5   4     1.118   109.999  -134.878  0.099096
   8  H21   hc    E    6   5   4     1.118   109.278   105.892  0.099096
   9  H22   hc    E    6   5   4     1.117   110.877   -13.933  0.099096
  10  C7    c3    M    5   4   3     1.536   121.605  -114.078  0.398600
  11  N5    n3    B   10   5   4     1.452   115.082   -18.456 -1.078483
  12  H18   hn    E   11  10   5     1.003   110.589    82.546  0.395569
  13  H19   hn    E   11  10   5     1.003   109.727   -37.047  0.395569
  14  H17   h1    E   10   5   4     1.134   106.154  -135.188 -0.005111
  15  C11   ca    M   10   5   4     1.507   108.201   107.985  0.039876
  16  C17   ca    B   15  10   5     1.397   120.601   -58.788 -0.157056
  17  C20   ca    S   16  15  10     1.391   121.121  -179.550 -0.266068
  18  H11   ha    E   17  16  15     1.102   119.020  -179.680  0.187381
  19  H9    ha    E   16  15  10     1.103   119.608     0.924  0.134061
  20  C16   ca    M   15  10   5     1.397   120.286   122.244 -0.157056
  21  H8    ha    E   20  15  10     1.102   119.942    -0.037  0.134061
  22  C19   ca    M   20  15  10     1.388   120.681   179.678 -0.266068
  23  H10   ha    E   22  20  15     1.101   118.994   179.919  0.187381
  24  C15   ca    M   22  20  15     1.418   120.447    -0.451  0.303723
  25  N4    n     M   24  22  20     1.405   118.100  -179.441 -0.631511
  26  H3    hn    E   25  24  22     0.998   115.443     2.090  0.302859
  27  C3    c     M   25  24  22     1.385   127.211  -174.463  0.990989
  28  O3    o     E   27  25  24     1.243   124.207    -1.335 -0.595940
  29  C1    cc    M   27  25  24     1.485   115.137   177.202 -0.527261
  30  N1    na    B   29  27  25     1.417   125.790    54.410  0.306437
  31  N2    nc    E   30  29  27     1.346   111.676  -179.241 -0.567024
  32  C9    ca    S   30  29  27     1.428   124.999    10.804  0.231494
  33  C21   ca    B   32  30  29     1.410   120.263    34.105 -0.243137
  34  C24   ca    B   33  32  30     1.392   119.591  -179.228 -0.081737
  35  C25   ca    B   34  33  32     1.395   120.577    -0.834 -0.187658
  36  C23   ca    B   35  34  33     1.394   119.814    -0.032 -0.081737
  37  C22   ca    S   36  35  34     1.393   120.653     0.416 -0.243137
  38  H13   ha    E   37  36  35     1.102   119.774  -179.365  0.157817
  39  H14   ha    E   36  35  34     1.100   120.026  -179.483  0.139297
  40  H16   ha    E   35  34  33     1.100   120.068  -179.808  0.146818
  41  H15   ha    E   34  33  32     1.101   119.364   179.515  0.139297
  42  H12   ha    E   33  32  30     1.103   120.714     0.859  0.157817
  43  C2    cd    M   29  27  25     1.398   127.887  -123.160  0.181850
  44  C6    c3    B   43  29  27     1.479   132.283    -2.239 -0.348922
  45  H1    h1    E   44  43  29     1.133   108.142   -70.003  0.144350
  46  H2    h1    E   44  43  29     1.131   109.735    47.569  0.144350
  47  C4    cd    M   43  29  27     1.455   104.984   178.349  0.303288
  48  C8    c3    M   47  43  29     1.477   121.106  -178.417 -0.119527
  49  H4    hc    E   48  47  43     1.124   109.297   103.830  0.091837
  50  H5    hc    E   48  47  43     1.122   111.250  -137.315  0.091837
  51  C10   c3    M   48  47  43     1.538   109.836   -17.164 -0.027986
  52  H6    h1    E   51  48  47     1.129   107.883   165.882  0.064293
  53  H7    h1    E   51  48  47     1.129   107.971   -77.189  0.064293
```

```
  54  N3    n3    M   51  48  47     1.440   114.901    44.335 -0.164549
  55  S1    sy    M   54  51  48     1.636   122.787   118.449  1.069585
  56  O1    o     E   55  54  51     1.411   109.649  -143.154 -0.554531
  57  O2    o     E   55  54  51     1.412   108.926   -14.501 -0.554531
  58  C5    cc    M   55  54  51     1.662   103.571   101.264 -0.126870
  59  C12   cd    M   58  55  54     1.405   120.232   100.927 -0.038440
  60  H23   ha    E   59  58  55     1.097   123.588    -0.710  0.155824
  61  C14   cd    M   59  58  55     1.413   112.275   178.329 -0.249804
  62  H25   ha    E   61  59  58     1.092   124.068  -179.939  0.182686
  63  C13   cc    M   61  59  58     1.392   110.729    -0.015 -0.141245
  64  H24   h4    E   63  61  59     1.091   124.272  -179.907  0.207238
  65  S2    ss    M   63  61  59     1.637   111.725    -0.267  0.052273


LOOP
  C15  C20
   C4   N2
  C22   C9
   N3   C6
   S2   C5

IMPROPER
  C26   C7  C18   O4
   C7  C17  C11  C16
  C11  C20  C17   H9
  C17  C15  C20  H11
  C11  C19  C16   H8
  C16  C15  C19  H10
  C20  C19  C15   N4
   C3  C15   N4   H3
   C1   N4   C3   O3
   C3   C2   C1   N1
   C9   C1   N1   N2
  C21  C22   C9   N1
   C9  C24  C21  H12
  C21  C25  C24  H15
  C24  C23  C25  H16
  C25  C22  C23  H14
   C9  C23  C22  H13
   C6   C1   C2   C4
   C8   C2   C4   N2
  C12   S2   C5   S1
   C5  C14  C12  H23
  C13  C12  C14  H25
  C14  H24  C13   S2

DONE
STOP
```

l96.in

```
    0    0    2

This is a remark line
molecule.res
L96   INT  0
CORRECT     OMIT DU   BEG
  0.0000
   1  DUMM  DU    M    0  -1  -2     0.000      .0        .0      .00000
   2  DUMM  DU    M    1   0  -1     1.449      .0        .0      .00000
   3  DUMM  DU    M    2   1   0     1.523   111.21       .0      .00000
   4  C25   c3    M    3   2   1     1.540   111.208  -180.000 -0.238249
   5  H20   h1    E    4   3   2     1.121    56.497   170.769  0.092280
   6  H21   h1    E    4   3   2     1.124   155.322  -131.197  0.092280
   7  H22   h1    E    4   3   2     1.125    64.907   -51.937  0.092280
   8  N5    n     M    4   3   2     1.432    94.606    58.348 -0.039247
   9  C26   c3    3    8   4   3     1.437   116.482  -146.631 -0.238249
  10  H17   h1    E    9   8   4     1.122   110.383   173.703  0.092280
  11  H18   h1    E    9   8   4     1.123   109.518    53.400  0.092280
  12  H19   h1    E    9   8   4     1.124   110.183   -66.010  0.092280
  13  C24   c     M    8   4   3     1.388   123.092    36.498  0.508905
  14  O4    o     E   13   8   4     1.249   120.120   173.606 -0.583476
  15  C10   ca    M   13   8   4     1.490   119.286    -6.449 -0.054264
  16  C16   ca    B   15  13   8     1.398   118.765   129.650 -0.082369
  17  C18   ca    S   16  15  13     1.391   120.831   177.539 -0.334387
  18  H11   ha    E   17  16  15     1.102   119.004   179.663  0.187107
  19  H9    ha    E   16  15  13     1.103   119.512    -1.712  0.154831
  20  C15   ca    M   15  13   8     1.398   121.669   -53.910 -0.082369
  21  H8    ha    E   20  15  13     1.102   119.976     2.599  0.154831
  22  C17   ca    M   20  15  13     1.387   120.502  -176.895 -0.334387
  23  H10   ha    E   22  20  15     1.102   118.986   179.415  0.187107
  24  C14   ca    M   22  20  15     1.419   120.461    -0.255  0.437896
  25  N4    n     M   24  22  20     1.403   117.992  -179.497 -0.723404
  26  H3    hn    E   25  24  22     0.999   115.657     0.229  0.368738
  27  C3    c     M   25  24  22     1.387   127.344  -177.355  0.948672
  28  O3    o     E   27  25  24     1.243   124.315    -2.336 -0.547500
  29  C1    cc    M   27  25  24     1.487   113.712   176.208 -0.545292
  30  N1    na    B   29  27  25     1.410   125.955   121.274  0.375601
  31  N2    nc    E   30  29  27     1.345   111.698  -179.249 -0.565532
  32  C8    ca    S   30  29  27     1.431   126.184    -3.963  0.138964
  33  C19   ca    B   32  30  29     1.408   120.296    54.326 -0.211962
  34  C22   ca    B   33  32  30     1.393   119.469   179.572 -0.083644
  35  C23   ca    B   34  33  32     1.394   120.655    -0.777 -0.169887
  36  C21   ca    B   35  34  33     1.395   119.833     0.129 -0.083644
  37  C20   ca    S   36  35  34     1.393   120.552     0.204 -0.211962
  38  H13   ha    E   37  36  35     1.101   119.635  -179.411  0.160804
  39  H14   ha    E   36  35  34     1.100   120.029  -179.675  0.129419
  40  H16   ha    E   35  34  33     1.100   120.068  -179.747  0.137962
  41  H15   ha    E   34  33  32     1.100   119.277   179.542  0.129419
  42  H12   ha    E   33  32  30     1.104   120.707    -1.461  0.160804
  43  C2    cd    M   29  27  25     1.401   127.719   -59.249  0.137912
  44  C6    c3    B   43  29  27     1.479   132.735    -2.365 -0.155598
  45  H1    h1    E   44  43  29     1.132   108.471   -73.906  0.100906
```

```
  46  H2    h1    E   44  43  29     1.131   110.213    44.144  0.100906
  47  C4    cd    M   43  29  27     1.451   105.004   179.758  0.282686
  48  C7    c3    M   47  43  29     1.479   121.576  -178.202 -0.108808
  49  H4    hc    E   48  47  43     1.123   109.181   106.516  0.090415
  50  H5    hc    E   48  47  43     1.122   111.052  -134.787  0.090415
  51  C9    c3    M   48  47  43     1.538   110.309   -14.502 -0.014598
  52  H6    h1    E   51  48  47     1.129   107.893   162.536  0.071306
  53  H7    h1    E   51  48  47     1.129   108.051   -80.596  0.071306
  54  N3    n3    M   51  48  47     1.441   115.106    40.962 -0.300301
  55  S1    sy    M   54  51  48     1.635   123.195   115.426  1.117157
  56  O1    o     E   55  54  51     1.413   109.206  -143.408 -0.559718
  57  O2    o     E   55  54  51     1.412   109.136   -14.763 -0.559718
  58  C5    cc    M   55  54  51     1.662   103.525   101.185 -0.144766
  59  C11   cd    M   58  55  54     1.405   120.245   100.921 -0.040543
  60  H23   ha    E   59  58  55     1.096   123.651    -0.593  0.146237
  61  C13   cd    M   59  58  55     1.413   112.264   178.332 -0.239435
  62  H24   ha    E   61  59  58     1.092   124.055  -179.934  0.180882
  63  C12   cc    M   61  59  58     1.393   110.713    -0.003 -0.148682
  64  H25   h4    E   63  61  59     1.091   124.179  -179.893  0.208670
  65  S2    ss    M   63  61  59     1.636   111.722    -0.269  0.068452


LOOP
  C14  C18
   C4   N2
  C20   C8
   N3   C6
   S2   C5

IMPROPER
  C24  C25   N5  C26
  C10   N5  C24   O4
  C24  C16  C10  C15
  C10  C18  C16   H9
  C16  C14  C18  H11
  C10  C17  C15   H8
  C15  C14  C17  H10
  C18  C17  C14   N4
   C3  C14   N4   H3
   C1   N4   C3   O3
   C3   C2   C1   N1
   C8   C1   N1   N2
  C19  C20   C8   N1
   C8  C22  C19  H12
  C19  C23  C22  H15
  C22  C21  C23  H16
  C23  C20  C21  H14
   C8  C21  C20  H13
   C6   C1   C2   C4
   C7   C2   C4   N2
  C11   S2   C5   S1
   C5  C13  C11  H23
  C12  C11  C13  H24
  C13  H25  C12   S2

DONE
STOP
```

l98.in

```
    0    0    2

This is a remark line
molecule.res
L98   INT  0
CORRECT     OMIT DU   BEG
  0.0000
   1  DUMM  DU    M    0  -1  -2     0.000      .0        .0      .00000
   2  DUMM  DU    M    1   0  -1     1.449      .0        .0      .00000
   3  DUMM  DU    M    2   1   0     1.523   111.21       .0      .00000
   4  C25   c3    M    3   2   1     1.540   111.208  -180.000  0.108216
   5  H20   h1    E    4   3   2     1.124   143.968  -146.598  0.030404
   6  H21   h1    E    4   3   2     1.124    79.545   -39.421  0.030404
   7  H23   h1    E    4   3   2     1.123   100.879    68.179  0.030404
   8  N5    n     M    4   3   2     1.427    37.511   177.296 -0.573203
   9  H22   hn    E    8   4   3     0.991   117.754    96.555  0.301401
  10  C24   c     M    8   4   3     1.382   122.756   -91.623  0.703632
  11  O4    o     E   10   8   4     1.249   121.081     2.345 -0.574604
  12  C10   ca    M   10   8   4     1.489   117.403  -177.402 -0.238289
  13  C16   ca    B   12  10   8     1.398   118.312   144.533 -0.004439
  14  C18   ca    S   13  12  10     1.391   120.988   179.681 -0.345248
  15  H11   ha    E   14  13  12     1.103   119.031   179.676  0.186366
  16  H9    ha    E   13  12  10     1.103   119.192     0.203  0.127671
  17  C15   ca    M   12  10   8     1.398   122.373   -36.951 -0.004439
  18  H8    ha    E   17  12  10     1.101   120.667    -0.490  0.127671
  19  C17   ca    M   17  12  10     1.388   120.552  -179.203 -0.345248
  20  H10   ha    E   19  17  12     1.101   119.042   179.551  0.186366
  21  C14   ca    M   19  17  12     1.419   120.471    -0.189  0.442147
  22  N4    n     M   21  19  17     1.403   117.979  -179.386 -0.720961
  23  H3    hn    E   22  21  19     1.000   115.704     0.936  0.365730
  24  C3    c     M   22  21  19     1.388   127.327  -176.913  0.956246
  25  O3    o     E   24  22  21     1.241   124.294    -1.896 -0.542749
  26  C1    cc    M   24  22  21     1.487   113.640   176.618 -0.566904
  27  N1    na    B   26  24  22     1.411   125.980   121.072  0.389538
  28  N2    nc    E   27  26  24     1.344   111.717  -179.256 -0.568332
  29  C8    ca    S   27  26  24     1.431   126.095    -3.940  0.133967
  30  C19   ca    B   29  27  26     1.408   120.325    54.271 -0.211343
  31  C22   ca    B   30  29  27     1.393   119.487   179.514 -0.083560
  32  C23   ca    B   31  30  29     1.394   120.643    -0.739 -0.170491
  33  C21   ca    B   32  31  30     1.394   119.852     0.058 -0.083560
  34  C20   ca    S   33  32  31     1.392   120.600     0.265 -0.211343
  35  H13   ha    E   34  33  32     1.101   119.762  -179.440  0.159749
  36  H14   ha    E   33  32  31     1.101   120.064  -179.715  0.130496
```

```
  37  H16   ha    E   32  31  30     1.100   120.066  -179.820  0.138673
  38  H15   ha    E   31  30  29     1.101   119.272   179.421  0.130496
  39  H12   ha    E   30  29  27     1.104   120.711    -1.476  0.159749
  40  C2    cd    M   26  24  22     1.401   127.721   -59.481  0.150021
  41  C6    c3    B   40  26  24     1.479   132.745    -2.388 -0.150483
  42  H1    h1    E   41  40  26     1.133   108.431   -73.921  0.099836
  43  H2    h1    E   41  40  26     1.131   110.194    44.090  0.099836
  44  C4    cd    M   40  26  24     1.450   105.020   179.734  0.284160
  45  C7    c3    M   44  40  26     1.478   121.629  -178.203 -0.122746
  46  H4    hc    E   45  44  40     1.124   109.202   106.520  0.094388
  47  H5    hc    E   45  44  40     1.122   111.181  -134.790  0.094388
  48  C9    c3    M   45  44  40     1.539   110.326   -14.441 -0.011456
  49  H6    h1    E   48  45  44     1.129   107.942   162.447  0.068470
  50  H7    h1    E   48  45  44     1.129   108.022   -80.652  0.068470
  51  N3    n3    M   48  45  44     1.441   115.093    40.832 -0.296708
  52  S1    sy    M   51  48  45     1.637   123.138   115.588  1.115779
  53  O1    o     E   52  51  48     1.413   109.195  -143.948 -0.559552
  54  O2    o     E   52  51  48     1.412   109.136   -15.288 -0.559552
  55  C5    cc    M   52  51  48     1.661   103.534   100.622 -0.144189
  56  C11   cd    M   55  52  51     1.405   120.265   100.796 -0.037904
  57  H17   ha    E   56  55  52     1.097   123.588    -0.596  0.143392
  58  C13   cd    M   56  55  52     1.412   112.312   178.386 -0.239148
  59  H19   ha    E   58  56  55     1.092   124.077  -179.892  0.181142
  60  C12   cc    M   58  56  55     1.392   110.702    -0.108 -0.152842
  61  H18   h4    E   60  58  56     1.092   124.235  -179.880  0.210822
  62  S2    ss    M   60  58  56     1.636   111.765    -0.162  0.069263


LOOP
  C14  C18
   C4   N2
  C20   C8
   N3   C6
   S2   C5

IMPROPER
  C25  C24   N5  H22
  C10   N5  C24   O4
  C24  C16  C10  C15
  C10  C18  C16   H9
  C16  C14  C18  H11
  C10  C17  C15   H8
  C15  C14  C17  H10
  C18  C17  C14   N4
   C3  C14   N4   H3
   C1   N4   C3   O3
   C3   C2   C1   N1
   C8   C1   N1   N2
  C19  C20   C8   N1
   C8  C22  C19  H12
  C19  C23  C22  H15
  C22  C21  C23  H16
  C23  C20  C21  H14
   C8  C21  C20  H13
   C6   C1   C2   C4
   C7   C2   C4   N2
  C11   S2   C5   S1
   C5  C13  C11  H17
  C12  C11  C13  H19
  C13  H18  C12   S2

DONE
STOP
```

l99.in

```
    0    0    2

This is a remark line
molecule.res
L99   INT  0
CORRECT     OMIT DU   BEG
  0.0000
   1  DUMM  DU    M    0  -1  -2     0.000      .0        .0      .00000
   2  DUMM  DU    M    1   0  -1     1.449      .0        .0      .00000
   3  DUMM  DU    M    2   1   0     1.523   111.21       .0      .00000
   4  C15   ca    M    3   2   1     1.540   111.208  -180.000 -0.293956
   5  C17   ca    S    4   3   2     1.386   126.587     2.112 -0.252606
   6  H10   ha    E    5   4   3     1.101   118.671   -68.057  0.197983
   7  H8    ha    E    4   3   2     1.099    55.713  -103.439  0.189508
   8  C10   ca    M    4   3   2     1.404    88.245   128.316  0.392814
   9  O4    oh    S    8   4   3     1.377   116.568    48.090 -0.570061
  10  H17   ho    E    9   8   4     0.969   107.783   179.033  0.382061
  11  C16   ca    M    8   4   3     1.399   120.445  -131.878 -0.293956
  12  H9    ha    E   11   8   4     1.099   120.333  -179.830  0.189508
  13  C18   ca    M   11   8   4     1.392   120.061     0.085 -0.252606
  14  H11   ha    E   13  11   8     1.103   118.762  -179.844  0.197983
  15  C14   ca    M   13  11   8     1.410   120.388    -0.094  0.319879
  16  N4    n     M   15  13  11     1.408   123.187   179.295 -0.688556
  17  H3    hn    E   16  15  13     0.998   115.333  -177.171  0.329126
  18  C3    c     M   16  15  13     1.383   127.121     7.905  0.994220
  19  O3    o     E   18  16  15     1.245   124.386    -2.345 -0.619087
  20  C1    cc    M   18  16  15     1.486   115.048   176.602 -0.484337
  21  N1    na    B   20  18  16     1.416   125.653    57.181  0.275576
  22  N2    nc    E   21  20  18     1.347   111.739  -179.424 -0.558983
  23  C8    ca    S   21  20  18     1.428   125.016    10.341  0.248215
  24  C19   ca    B   23  21  20     1.410   120.289    33.954 -0.256978
  25  C22   ca    B   24  23  21     1.393   119.537  -179.209 -0.075663
  26  C23   ca    B   25  24  23     1.394   120.642    -0.853 -0.184684
  27  C21   ca    B   26  25  24     1.394   119.828     0.028 -0.075663
  28  C20   ca    S   27  26  25     1.393   120.623     0.374 -0.256978
  29  H13   ha    E   28  27  26     1.102   119.694  -179.407  0.162698
  30  H14   ha    E   27  26  25     1.100   120.049  -179.507  0.135087
```

```
  31  H16    ha    E   26  25  24     1.100   120.088  -179.791  0.143405
  32  H15    ha    E   25  24  23     1.101   119.284   179.544  0.135087
  33  H12    ha    E   24  23  21     1.102   120.713     0.765  0.162698
  34  C2     cd    M   20  18  16     1.398   128.070  -120.610  0.151204
  35  C6     c3    B   34  20  18     1.479   132.260    -2.059 -0.287773
  36  H1     h1    E   35  34  20     1.133   108.162   -69.563  0.132525
  37  H2     h1    E   35  34  20     1.131   109.720    48.066  0.132525
  38  C4     cd    M   34  20  18     1.455   105.007   178.592  0.303020
  39  C7     c3    M   38  34  20     1.478   121.158  -178.576 -0.129247
  40  H4     hc    E   39  38  34     1.123   109.329   103.539  0.093696
  41  H5     hc    E   39  38  34     1.122   111.306  -137.453  0.093696
  42  C9     c3    M   39  38  34     1.538   109.726   -17.344 -0.014815
  43  H6     h1    E   42  39  38     1.129   107.908   166.129  0.060737
  44  H7     h1    E   42  39  38     1.129   107.967   -76.984  0.060737
  45  N3     n3    M   42  39  38     1.441   114.916    44.543 -0.190296
  46  S1     sy    M   45  42  39     1.635   122.753   118.478  1.076752
  47  O1     o     E   46  45  42     1.411   109.754  -142.820 -0.555629
  48  O2     o     E   46  45  42     1.413   109.015   -14.089 -0.555629
  49  C5     cc    M   46  45  42     1.664   103.519   101.598 -0.124920
  50  C11    cd    M   49  46  45     1.404   120.187   101.547 -0.042493
  51  H18    ha    E   50  49  46     1.097   123.577    -0.697  0.155853
  52  C13    cd    M   50  49  46     1.413   112.260   178.299 -0.247605
  53  H20    ha    E   52  50  49     1.092   124.035  -179.857  0.180291
  54  C12    cc    M   52  50  49     1.391   110.728    -0.042 -0.141019
  55  H19    h4    E   54  52  50     1.091   124.253  -179.928  0.205818
  56  S2     ss    M   54  52  50     1.637   111.748    -0.210  0.050838


LOOP
  C14  C17
   C4   N2
  C20   C8
   N3   C6
   S2   C5

IMPROPER
  C17  C10  C15   H8
  C14  C15  C17  H10
  C16  C15  C10   O4
  C18  C10  C16   H9
  C16  C14  C18  H11
  C18  C17  C14   N4
   C3  C14   N4   H3
   C1   N4   C3   O3
   C3   C2   C1   N1
   C8   C1   N1   N2
  C19  C20   C8   N1
   C8  C22  C19  H12
  C19  C23  C22  H15
  C22  C21  C23  H16
  C23  C20  C21  H14
   C8  C21  C20  H13
   C6   C1   C2   C4
   C7   C2   C4   N2
  C11   S2   C5   S1
   C5  C13  C11  H18
  C12  C11  C13  H20
  C13  H19  C12   S2

DONE
STOP
```

lm1.in

```
    0    0    2

This is a remark line
molecule.res
LM1   INT  0
CORRECT     OMIT DU   BEG
  0.0000
   1  DUMM   DU    M    0  -1  -2     0.000      .0        .0      .00000
   2  DUMM   DU    M    1   0  -1     1.449      .0        .0      .00000
   3  DUMM   DU    M    2   1   0     1.523   111.21       .0      .00000
   4  C27    c3    M    3   2   1     1.540   111.208  -180.000 -0.048073
   5  H22    h1    E    4   3   2     1.117    90.513   -21.373  0.092585
   6  H23    h1    E    4   3   2     1.118   154.141  -170.557  0.092585
   7  H24    h1    E    4   3   2     1.116    70.591    89.728  0.092585
   8  O5     os    M    4   3   2     1.429    54.280  -135.838 -0.438165
   9  C12    c     M    8   4   3     1.372   116.363    14.834  0.885605
  10  O4     o     E    9   8   4     1.235   117.678     0.046 -0.601795
  11  C11    ca    M    9   8   4     1.470   114.362  -179.902 -0.264740
  12  C10    ca    B   11   9   8     1.402   118.477  -179.703 -0.030798
  13  C9     ca    S   12  11   9     1.392   119.823   179.930 -0.237297
  14  H7     ha    E   13  12  11     1.103   120.386  -179.971  0.153613
  15  H8     ha    E   12  11   9     1.103   119.457     0.017  0.128664
  16  C8     ca    M   11   9   8     1.399   121.398     0.322 -0.030798
  17  H6     ha    E   16  11   9     1.102   119.759     0.034  0.128664
  18  C7     ca    M   16  11   9     1.394   119.784   179.950 -0.237297
  19  H5     ha    E   18  16  11     1.101   119.669  -179.748  0.153613
  20  C6     ca    M   18  16  11     1.397   120.198     0.199  0.241841
  21  C5     c3    M   20  18  16     1.505   120.390   178.138 -0.305484
  22  H3     h1    E   21  20  18     1.129   109.485   -12.195  0.152193
  23  H4     h1    E   21  20  18     1.132   108.129  -130.389  0.152193
  24  N1     n     M   21  20  18     1.435   115.262   109.904 -0.258003
  25  C16    c     S   24  21  20     1.416   124.687    85.279  0.625191
  26  O1     o     E   25  24  21     1.236   123.525     6.361 -0.523703
  27  C4     ca    M   24  21  20     1.408   124.987   -84.112  0.287169
  28  C3     ca    M   27  24  21     1.394   129.536    -7.274 -0.330813
  29  H2     ha    E   28  27  24     1.099   121.455     2.036  0.170460
  30  C2     ca    M   28  27  24     1.399   118.340  -178.359  0.047511
  31  H1     ha    E   30  28  27     1.102   118.476   179.983  0.119658
  32  C1     ca    M   30  28  27     1.395   121.352    -0.079 -0.176002
```

```
  33  Br1   br    E   32  30  28     1.871   119.988   179.972 -0.067984
  34  C13   ca    M   32  30  28     1.406   120.619    -0.094 -0.006785
  35  H9    ha    E   34  32  30     1.101   120.822  -179.855  0.146701
  36  C14   ca    M   34  32  30     1.379   118.782     0.086 -0.236981
  37  C15   c3    M   36  34  32     1.511   130.036  -179.957  0.138711
  38  C20   c3    3   37  36  34     1.523   111.387    61.525 -0.210198
  39  C19   c3    B   38  37  36     1.531   111.993  -174.435  0.132012
  40  H14   h1    E   39  38  37     1.131   108.165   -69.824  0.024226
  41  H15   h1    E   39  38  37     1.128   108.420   172.289  0.024226
  42  H16   hc    E   38  37  36     1.120   110.140    64.998  0.095202
  43  H17   hc    E   38  37  36     1.122   108.245   -52.803  0.095202
  44  C17   c3    M   37  36  34     1.523   111.350   -63.231 -0.210198
  45  H10   hc    E   44  37  36     1.123   108.258    52.911  0.095202
  46  H11   hc    E   44  37  36     1.121   110.165   -64.862  0.095202
  47  C18   c3    M   44  37  36     1.530   111.943   174.567  0.132012
  48  H12   h1    E   47  44  37     1.128   108.881  -172.773  0.024226
  49  H13   h1    E   47  44  37     1.130   108.373    69.593  0.024226
  50  N2    n3    M   47  44  37     1.442   113.405   -51.325 -0.325319
  51  S1    sy    M   50  47  44     1.620   123.149  -126.615  1.127383
  52  O2    o     E   51  50  47     1.415   110.017   151.162 -0.571822
  53  O3    o     E   51  50  47     1.409   110.313    21.884 -0.571822
  54  C21   ca    M   51  50  47     1.706   103.596   -94.293 -0.233182
  55  C22   ca    M   54  51  50     1.405   117.690  -119.933 -0.036358
  56  H18   ha    E   55  54  51     1.107   118.646     2.015  0.164027
  57  C23   ca    M   55  54  51     1.389   121.849  -177.758 -0.240047
  58  H19   ha    E   57  55  54     1.100   120.054   179.849  0.159069
  59  C24   ca    M   57  55  54     1.397   119.894    -0.075 -0.016442
  60  H20   ha    E   59  57  55     1.102   120.063   179.861  0.142516
  61  C26   ca    M   59  57  55     1.389   120.174    -0.066 -0.323791
  62  H21   ha    E   61  59  57     1.099   121.155  -179.961  0.189499
  63  C25   ca    M   61  59  57     1.409   119.464     0.001  0.418092
  64  F1    f     M   63  61  59     1.353   118.473  -179.645 -0.217967


LOOP
   C6   C9
  C15  C16
  C14   C4
   N2  C19
  C25  C21

IMPROPER
  C11   O4  C12   O5
  C12  C10  C11   C8
  C11   C9  C10   H8
  C10   C6   C9   H7
  C11   C7   C8   H6
   C8   C6   C7   H5
   C5   C9   C6   C7
  C16   C5   N1   C4
  C15   N1  C16   O1
   C3  C14   C4   N1
   C4   C2   C3   H2
   C3   C1   C2   H1
  Br1   C2   C1  C13
   C1  C14  C13   H9
  C15   C4  C14  C13
  C22  C25  C21   S1
  C21  C23  C22  H18
  C22  C24  C23  H19
  C23  C26  C24  H20
  C24  C25  C26  H21
  C21  C26  C25   F1

DONE
STOP
```
